# Evaluating the impact of medication review and deprescribing on prescribing appropriateness and clinical outcomes in older people residing in long-term care facilities: a systematic review and meta-analysis

**DOI:** 10.1093/ageing/afag084

**Published:** 2026-04-12

**Authors:** Massimo Carollo, Irene Cristini, Salvatore Crisafulli, Andrea Fontana, Anna Forti, Aurora Lanaro, Francesco Maccarrone, Marta Zerio, Luca Piccoli, Elisabetta Poluzzi, Graziano Onder, Gianluca Trifirò

**Affiliations:** Department of Diagnostics and Public Health, University of Verona, Verona, 37134, Veneto, Italy; Department of Diagnostics and Public Health, University of Verona, Verona, 37134, Veneto, Italy; Department of Diagnostics and Public Health, University of Verona, Verona, 37134, Veneto, Italy; IRCCS Ospedale Casa Sollievo della Sofferenza, Unit of Biostatistics, San Giovanni Rotondo, 71013 Apulia, Italy; Department of Diagnostics and Public Health, University of Verona, Verona, 37134, Veneto, Italy; Department of Diagnostics and Public Health, University of Verona, Verona, 37134, Veneto, Italy; Department of Diagnostics and Public Health, University of Verona, Verona, 37134, Veneto, Italy; Department of Diagnostics and Public Health, University of Verona, Verona, 37134, Veneto, Italy; Department of Diagnostics and Public Health, University of Verona, Verona, 37134, Veneto, Italy; Department of Medical and Surgical Sciences, University of Bologna, Bologna, 40138, Emilia-Romagna, Italy; Department of Geriatrics, Orthopedics and Rheumatology, Università Cattolica del Sacro Cuore, Rome, 00168, Lazio, Italy; Department of Diagnostics and Public Health, University of Verona, Verona, 37134, Veneto, Italy

**Keywords:** medication review, deprescribing, patient-centred care, polypharmacy, long-term care facilities, systematic review, older people

## Abstract

**Background:**

Polypharmacy is a major concern among older adults in long-term care facilities (LTCFs), as it increases the risk of potentially inappropriate medications (PIMs) and related adverse outcomes. Medication review and deprescribing interventions may help optimise therapy and reduce harm.

**Design:**

Systematic review and meta-analysis.

**Methods:**

This study was conducted according to PRISMA guidelines (PROSPERO: CRD42023486056). PubMed, Embase and Scopus were searched up to 27 August 2024, for experimental studies evaluating the impact of medication review/deprescribing interventions in older LTCF residents with polypharmacy. Outcomes included medication appropriateness indexes, falls, hospitalisations and mortality. We calculated risk ratios for dichotomous data and mean differences for continuous data [with 95% confidence intervals (CIs)]. The quality of the studies was assessed using RoB 2 for the randomised controlled trials (RCTs) and the ROBINS-I for non-randomised studies.

**Results:**

From 3548 records, 38 studies (22 RCTs, 16 quasi-experimental) were included. Pooled analyses demonstrated significant reductions in the number of drugs per patient [within 12 months: −0.89 (95% CI −1.46, −0.32); at ≥12 months: −1.60 (95% CI −2.68, −0.52)] and in PIMs [at 6 months: −0.48 (95% CI −0.74, −0.22); at ≥12 months: −0.26 (95% CI −0.40, −0.13)]. No significant effects were observed on falls, hospitalisations or mortality. Studies showed wide methodological heterogeneity and had moderate to high risk of bias (23 moderate, 14 high, 1 low).

**Conclusions:**

Comprehensive medication review interventions improved prescribing appropriateness in older LTCF residents with polypharmacy but did not significantly affect clinical outcomes (i.e. falls, hospitalisations and mortality). Further high-quality studies using standardised approaches are needed.

## Key points

- Medication review and deprescribing safely reduced polypharmacy and PIM prevalence in older adults living in LTCFs.
- No significant impact was found on clinical outcomes such as falls, hospitalisations and mortality.
- High heterogeneity in study designs, intervention components and methodological quality was found.
- Future research should adopt robust, transparent and iterative approaches using multidimensional tools.

## Introduction

Over the last decades, life expectancy has significantly increased, leading to an increased number of older adults affected by multimorbidity and exposed to polypharmacy (i.e. the daily intake of ≥5 different drugs) [1]. The prevalence of polypharmacy in long-term care facilities (LTCFs), i.e. residential settings that provide continuous medical and nursing assistance to individuals who are unable to live independently, varies widely according to facility characteristics, geographical location and the definitions adopted, with reported rates as high as 91% [2]. A major concern related to polypharmacy is the use of potentially inappropriate medications (PIMs), defined as drugs whose risks outweigh benefits or that are no longer clinically indicated [3–5]. A systematic review of 21 observational studies reported a prevalence of PIM use in LTCFs ranging from 18.5% to 82.6%, depending on the geographical location and the assessment tool employed (e.g. American Geriatric Society Beers criteria, Screening Tool of Older Persons’ Prescriptions (STOPP) criteria, etc.) [6]. Another study meta-analysed 26 observational studies and found a weighted point prevalence of PIM use in nursing homes of 43.2% [7]. PIMs contribute to drug-related problems (DRPs) that are events or circumstances in which drug therapy interferes with intended clinical outcomes [8]. DRPs, including adverse drug reactions (ADRs), drug–drug interactions (DDIs), inappropriate dosing and poor adherence, are associated with higher rates of hospitalisation and mortality [9–12]. Nevertheless, the economic burden associated with PIMs is also substantial; for instance, costs related to preventable ADRs may range from a minimum of €174 (mean cost of an emergency department visit) to a maximum of €8515 (mean cost per hospital admission), frequently associated with prolonged lengths of hospital stay [11]. Hence, preventing PIM use is critical to reduce medication-related harm. In this regard, structured medication review and deprescribing interventions have proven effective in optimising therapeutic regimens [13–16]. Comprehensive medication review refers to a structured, systematic and patient-centred evaluation of all prescribed and non-prescribed medications, aimed at identifying DRPs and optimising pharmacological therapies. This evaluation can lead to deprescribing, that is the evidence-based withdrawal or dose reduction of unnecessary medications under clinical supervision. This approach is variably referred to in the literature as clinical medication review, structured medication review, medication therapy management or pharmacist-led medication review [17–20]. LTCFs offer favourable conditions for medication reviews and deprescribing, as physicians can regularly revise treatment plans and closely monitor clinical outcomes [17, 21].

In 2019, Kua *et al*. published a systematic review and meta-analysis of 41 randomised controlled trials (RCTs) in nursing home residents, showing that medication review and deprescribing reduced PIMs, falls and mortality [22]. However, the analysis only included RCTs published up to September 2017, mostly targeting specific drugs or conditions, limiting comparability and generalizability. To address this gap, we conducted a systematic review and meta-analysis to evaluate the impact of comprehensive medication review and deprescribing interventions on prescribing appropriateness and clinical outcomes among LTCF residents receiving polypharmacy, including more recent evidence from both RCTs and quasi-experimental studies.

## Methods

### Search strategy and selection criteria

This systematic review was carried out according to the Preferred Reporting Items for Systematic Reviews and Meta-Analyses (PRISMA) [23] and the study protocol was registered on PROSPERO (CRD42023486056). All clinical studies evaluating the efficacy/effectiveness of medication review and deprescribing interventions among LTCF residents receiving polypharmacy were searched in the bibliographic databases PubMed (including PubMed Central and Medline), Embase and Scopus, from their inception until 27 August 2024. The search terms were related to medication review and deprescribing, LTCF settings and older patients. The detailed search strategy developed for each database is provided in Appendix Table A1.

Only experimental studies (including both RCTs and quasi-experimental studies) written in English and reporting the impact of medication review and deprescribing interventions in the LTCF setting (e.g. nursing homes/facilities, care homes/facilities, assisted living facilities, residential aged care, senior living residencies, residential care homes, retirement homes and homes for the aged) were included. Additionally, eligible studies had to assess at least one of the following clinical outcomes: incidence of ADRs, falls, emergency department (ED) visits, hospitalisations and mortality, as well as quality of life. Narrative or systematic reviews and meta-analyses, case reports, book chapters, editorials and conference abstracts were excluded, but they were screened to identify other potentially relevant studies. Studies that were restricted to deprescribing specific drug classes were not included. After removing duplicates, four review authors (AFor, AL, MZ and LP) individually screened titles and abstracts to identify and exclude clearly irrelevant articles. The selected full texts were then independently reviewed by two authors (MC and IC) to determine whether they met the inclusion criteria. Any disagreements among evaluators were resolved through discussion or, if consensus was not reached, through the intervention of a senior expert (GT).

### Data extraction

Data from each included article were independently extracted by two authors (MC and IC). Any discrepancies were resolved through discussion or the intervention of a third senior expert (GT). For each article, data on the following items were retrieved: author(s), year of publication, country/ies in which the study was conducted, number of enrolled participants, mean (± standard deviation, SD) or median (along with the interquartile range, IQR) age of the patients enrolled (or alternative measures if mean/median was not reported), enrolment criteria, healthcare professionals involved in the intervention (e.g. geriatricians, clinical pharmacologists, pharmacists and nurses), tools used for performing medication review, length of follow-up, study outcomes and results.

### Risk of bias assessment

The quality of the included studies was independently assessed by two authors (MC and IC), using version 2 of the Cochrane Risk of Bias Assessment Tool (RoB 2) for the RCTs and [24] the Risk of Bias in Non-randomised Studies - of Interventions (ROBINS-I) for non-randomised studies [25]. Plots were generated using the *robvis* visualisation tool [26].

### Statistical analysis

A meta-analysis was performed to assess the effect of the interventions on medication appropriateness indices (i.e. number of prescribed drugs and PIMs) and adverse clinical outcomes, including risk of falls, hospitalisation and mortality. For studies reporting the number or proportion of events, the risk ratio (RR) was calculated by comparing patients who underwent any medication review (intervention group) with those who did not (control group). If the RR was not directly reported or could not be calculated, study authors were contacted to provide the missing data. If this was not possible, the RR was estimated indirectly from the published hazard ratio (HR) using established conversion formulae, and its variance was calculated using the delta method (Appendix Statistical Methods). The RR was considered the reference risk measure because it was the most frequently reported parameter. The natural logarithm of the RR and its estimated variance were used to perform the meta-analysis, including subgroup analyses based on follow-up duration. When multiple measures were reported, only those being adjusted for confounders were selected.

For studies that reported outcomes as mean and SD (i.e. number of prescribed drugs, PIMs, falls and hospitalisations per patient), the mean difference was calculated by subtracting the mean in the control group from that in the intervention group. For each group, the standard error (SE) was calculated from the sample group size along with the SD, and the SE of the mean difference was obtained by combining the two SEs, assuming group independence. When study outcomes were only reported as medians and IQRs or ranges, conversion formulae were used to obtain approximate means and SDs [27].

Cochran’s Q test and the I^2^ measure were used to assess heterogeneity across study estimates. If moderate heterogeneity was detected (Q *P*-value <.10 or I^2^ > 40%) [28], a random-effects model was used; otherwise, a fixed-effects model was used. For outcomes reported as means, a univariate meta-analysis of mean differences was performed.

Meta-regression was performed to assess the contribution of study-level covariates (i.e. age at enrolment, follow-up, study type and geographical region) in reducing the between-study variance (τ^2^). Regarding geographical region, the country in which each study was conducted was classified into one of the following continents: America, Europe, Asia and Oceania. The magnitude of this reduction was quantified in terms of R^2^. In addition, for each study-level covariate considered, the omnibus Wald-type test was performed to statistically assess whether the covariate explained the study-level estimates.

Publication bias was assessed using funnel plots, and asymmetry was tested using Begg and Mazumdar’s rank correlation; these tests and meta-regression were not performed when fewer than 10 studies were included [29, 30]. Statistical analyses and graphs, including forest plots of epidemiological estimates with 95% confidence intervals (CIs), were performed using the ‘metafor’ package in R (version 4.4.1). A *P*-value <.05 was considered statistically significant.

## Results

### Study selection

**Figure 1:**
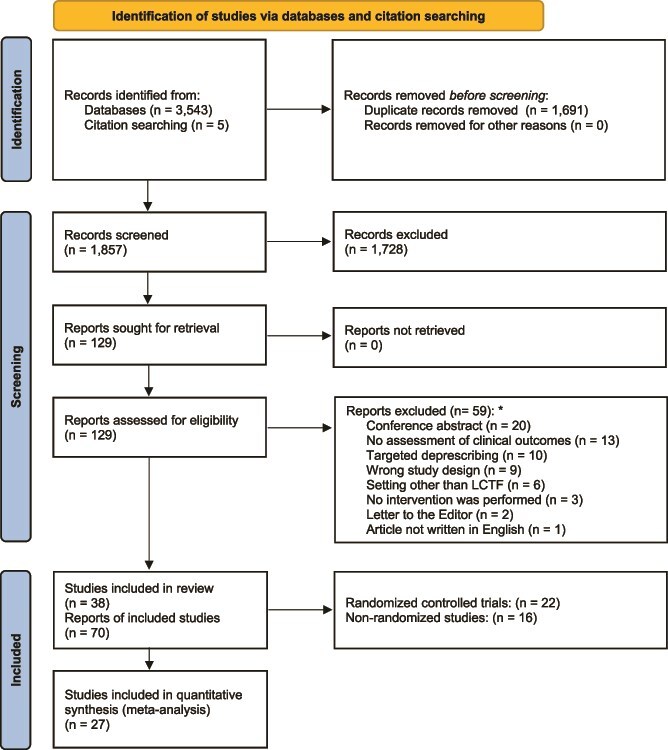
PRISMA flowchart showing the process of study selection. ^*^ In one case, there were two exclusion criteria (i.e. targeted deprescribing and the absence of clinical outcomes).

The flowchart summarising the process of study selection is shown in Figure 1. Our search strategy identified a total of 3543 records, and 5 further records were identified through citation searching. After removing duplicate records (*n* = 1691), 1857 (52.3%) titles and abstracts were screened, and 129 (3.6%) full-text articles were retained for further evaluation. Among these, 70 (54.3%) met the inclusion criteria and were included in the systematic review (Figure 1), for a total of 38 single studies (Appendix Table A2). Reasons for the exclusion of the remaining full-text articles are shown in Appendix Table A3.

### Study characteristics

The characteristics of the included studies are summarised in Appendix Table A4. Overall, 22 (57.9%) RCTs and 16 (42.1%) quasi-experimental studies were included in the systematic review. Most of them were conducted in Europe (*n* = 21; 55.3%), while the others were conducted in Oceania (*n* = 8; 21.1%), Asia (*n* = 5; 13.2%) and America (*n* = 4; 10.5%). More than half of these studies (*n* = 28, 73.7%) were published in the decade 2014–2024. The number of patients enrolled in the intervention group of each study ranged from 22 [31] to 4272 [32–34], and their mean/median age ranged from 78.9 [35] to 89.4 [36] years. The duration of the follow-up ranged from 1 [35, 37, 38] to 24 [39–41] months.

### Medication review and deprescribing interventions

In 8 (21.1%) [35, 42–55] out of the 38 studies included, medication review was conducted based only on the clinical expertise of the healthcare professionals involved in the care of the patients. In the remaining 31 studies (81.6%), different tools were systematically used, including national treatment guidelines, anticholinergic scales, deprescribing algorithms, criteria/checklists [e.g. the STOPP, Screening Tool to Alert to Right Treatment (START), American Geriatrics Society Beers criteria®], interaction checkers, pharmacological reference resources (i.e. British National Formulary and Summary of Product Characteristics) and/or multi-approach deprescribing tools (Appendix Tables A4 and A5). In all studies, either the facility physician or the general practitioner was involved in the intervention, while clinical pharmacists/pharmacologists and nurses were involved in 32 and 35 studies, respectively; in 5 studies, geriatricians were also involved (Appendix Table A4). In 16 (42.1%) [31–34, 37, 38, 42–46, 49–53, 56–78] studies, educational interventions were delivered to facility nurses, pharmacists and/or physicians.

### Effect of medication review and deprescribing interventions on medication appropriateness

**Figure 2:**
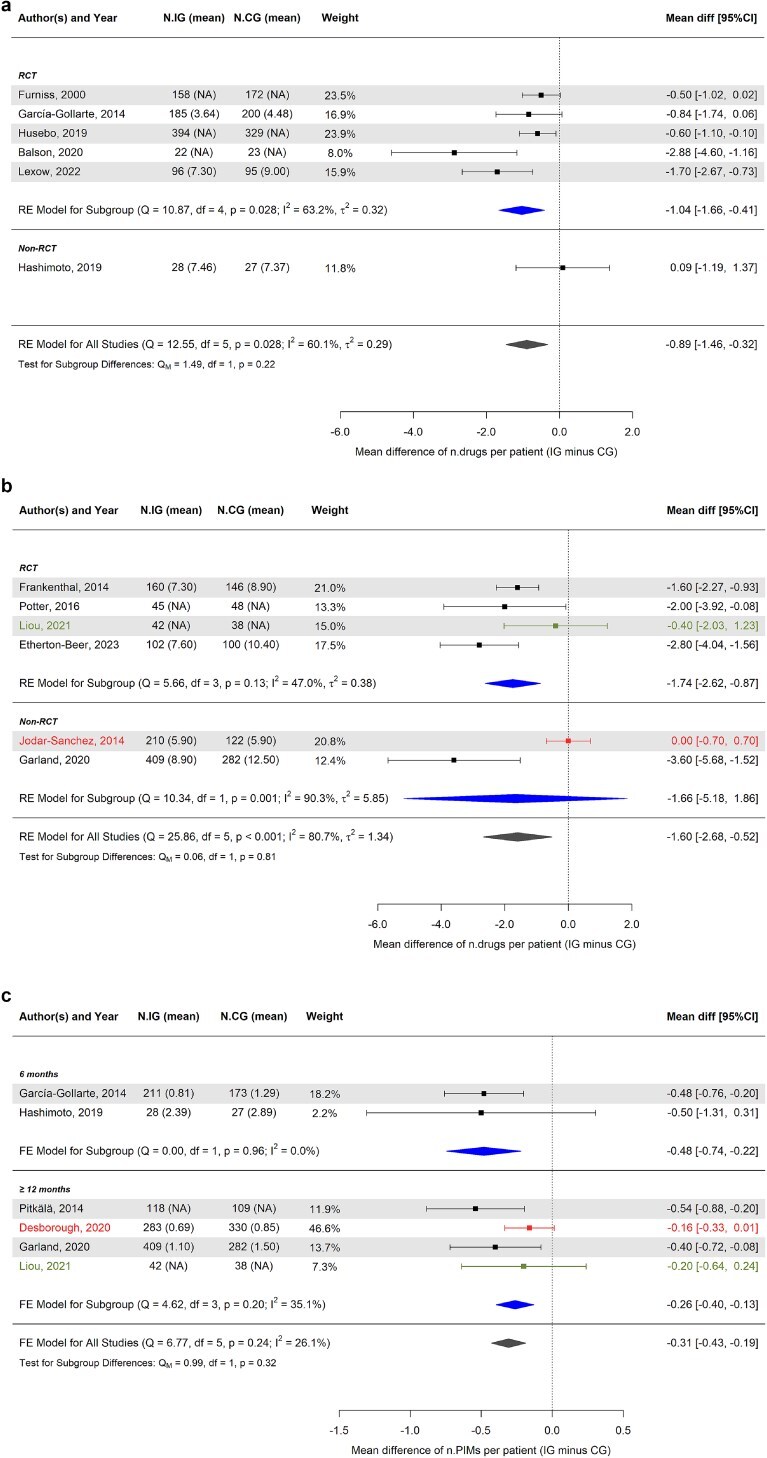
(a) Forest plot of the estimated mean differences (intervention minus control group) of the number of prescribed drugs per patient within 12 months. (b) Forest plot of the estimated mean differences (intervention minus control group) of the number of prescribed drugs per patient after 12 months. (c) Forest plot of the estimated mean differences (intervention minus control group) of the number of potentially inappropriate medications per patient at 6 or ≥ 12 months. The colours used for the horizontal lines indicate the type of intervention applied within each study: black for technology- or guideline-based interventions, green for training-based interventions and red for no specified intervention tools. Estimates are also stratified according to the study type or follow-up duration (subgroups). The summary polygon below each subgroup (filled in blue) shows the results of a fixed or random-effects model for just the studies within that group. The summary polygon at the bottom of the plot (filled in dark grey) shows the results of the model when all studies are analysed. The test for subgroup difference (QM) reflects the statistical significance of the subgroup covariate when included in a meta-regression model. Abbreviations: N.IG, number of valid patients in the intervention group (IG); N.CG, number of valid patients in the control group (CG); RCT, randomised clinical trials; FE, fixed-effects; RE, random-effects; Q, Cochran’s Q statistic, along with degrees of freedom (df) and its *P*-value; I^2^, inconsistency measure; τ^2^, between-study variance.

With the exception of the studies conducted by Roughead *et al*. [50–53] and Attwood *et al*. [36], all the included studies assessed medication appropriateness by evaluating the difference in the number of medications before and after the intervention, the frequency of prescriptions of PIMs, or DRPs such as therapeutic failure, underdosage, overdosage and/or non-adherence. In detail, 16 (42.1%) [32–34, 39–49, 56–60, 65–69, 76–82] studies assessed the reduction of PIMs at the end of follow-up. Some studies evaluated the medication burden (25, 65.8%) [31, 35, 37–42, 49, 54–56, 61–64, 75–79, 83–98], number of medication changes (3, 7.9%) [96–100] and Drug Burden Index (1, 2.6%) [70–74]. Twenty-seven (71.1%) [31–35, 39–49, 54–73, 76–79, 81, 85–92, 96–100] of the included studies employed statistical methods to evaluate the potential benefits of the intervention (2 studies did not assess medication appropriateness indices and the remaining 25 did not perform statistical analyses between intervention and control groups) and, among these, 17 (63.0%) [31, 39–41, 47–49, 54–64, 70–74, 76–78, 81, 85, 88–92, 99, 100] demonstrated a statistically significant positive impact of medication review and deprescribing interventions on at least 1 medication appropriateness index (Appendix Table A4). The meta-analysis of the number of prescribed drugs per patient showed an estimated mean difference of −0.89 (95% CI −1.46, −0.32) within 12 months (Figure 2a) and −1.60 (95% CI −2.68, −0.52) considering longer follow-up periods (Figure 2b). The meta-analysis of the number of PIMs per patient showed an estimated mean difference of −0.48 (95% CI −0.74, −0.22) at 6 months and −0.26 (95% CI −0.40, −0.13) considering follow-up periods of at least 12 months (Figure 2c).

### Effect of medication review and deprescribing interventions on the risk of falls

**Figure 3:**
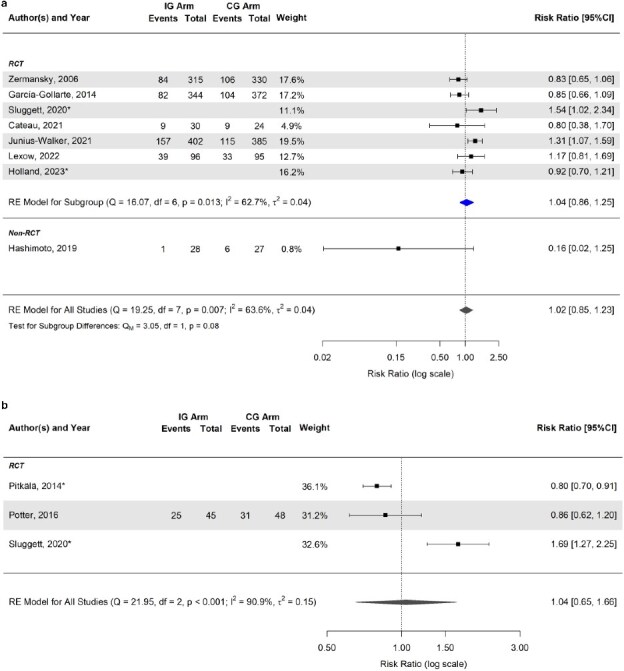
(a) Forest plot of the estimated risk ratio (intervention vs. control group) of falls at 3–6 months. (b) Forest plot of the estimated risk ratio (intervention vs. control group) of falls at 12 months. ^*^Risk ratio was estimated indirectly from the published hazard ratio, using established conversion formulae, and its variance was calculated using the delta method. All included studies involved technology- or guideline-based interventions. Estimates are also stratified according to the study type (subgroup). The summary polygon below each subgroup (filled in blue) shows the results of a fixed or random-effects model for just the studies within that group. The summary polygon at the bottom of the plot (filled in dark grey) shows the results of the model when all studies are analysed. The test for subgroup difference (QM) reflects the statistical significance of the subgroup covariate when included in a meta-regression model. Abbreviations: IG, intervention group; CG, control group; RCT, randomised clinical trials; FE, fixed-effects; RE, random-effects; Q, Cochran’s Q statistic, along with degrees of freedom (df) and its *P*-value; I^2^, inconsistency measure; τ^2^, between-study variance.

Overall, 15 studies (39.5%) [39–41, 47, 48, 56–60, 65–74, 79, 85–92, 96–100] assessed the impact of medication review and deprescribing interventions on the risk of falls (Appendix Table A4). Among these, 13 (86.7%) [39–41, 47, 48, 56–60, 65–74, 79, 85, 88–92, 98–100] could be included in the meta-analyses that showed no statistically significant risk difference between the intervention and control groups both at 3–6 months (RR 1.02; 95% CI 0.85, 1.23) and at 12 months (RR 1.04; 95% CI 0.65, 1.66) of follow-up (Figure 3). Forest plots of the estimated mean differences (intervention minus control group) in falls per patient are reported in Appendix Figures A1 and A2.

### Effect of medication review and deprescribing interventions on hospitalisation risk

**Figure 4:**
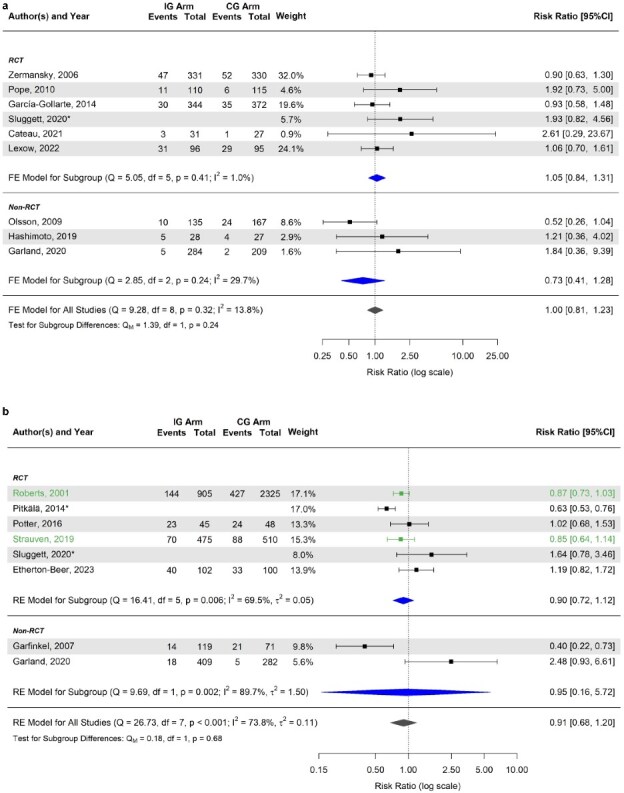
(a) Forest plot of the estimated risk ratio (intervention vs. control group) of hospitalisation at 3–6 months. (b) Forest plot of the estimated risk ratio (intervention vs. control group) of hospitalisation at 12–15 months. ^*^Risk ratio was estimated indirectly from the published hazard ratio, using established conversion formulae, and its variance was calculated using the delta method. The colours used for the horizontal lines indicate the type of intervention applied within each study: black for technology- or guideline-based interventions and green for training-based interventions. Estimates are also stratified according to the study type (subgroup). The summary polygon below each subgroup (filled in blue) shows the results of a fixed or random-effects model for just the studies within that group. The summary polygon at the bottom of the plot (filled in dark grey) shows the results of the model when all studies are analysed. The test for subgroup difference (QM) reflects the statistical significance of the subgroup covariate when included in a meta-regression model. Abbreviations: IG, intervention group; CG, control group; RCT, randomised clinical trials; FE, fixed-effects; RE, random-effects; Q, Cochran’s Q statistic, along with degrees of freedom (df) and its *P*-value; I^2^, inconsistency measure; τ^2^, between-study variance.

Overall, 26 studies (68.4%) [32–34, 39–46, 49, 54, 56–60, 65–75, 78–80, 82–92, 94, 96–100] assessed the impact of medication review and deprescribing interventions on hospital length of stay or hospitalisation risk (Appendix Table A4). Among these, 18 studies (69.2%) [39–41, 43–46, 56–60, 65–74, 78, 79, 83–85, 88–92, 98–100] were included in the meta-analysis, which showed no statistically significant differences between intervention and usual care concerning the hospitalisation risk both at 3–6 months (RR 1.00; 95% CI 0.81, 1.23) (Figure 4a) and at 12–15 months of follow-up (RR 0.91; 95% CI 0.68, 1.20) (Figure 4b). The forest plot of the estimated mean differences (intervention minus control group) in hospitalisations per patient is reported in Appendix Figure A3.

### Effect of medication review and deprescribing interventions on mortality risk

**Figure 5:**
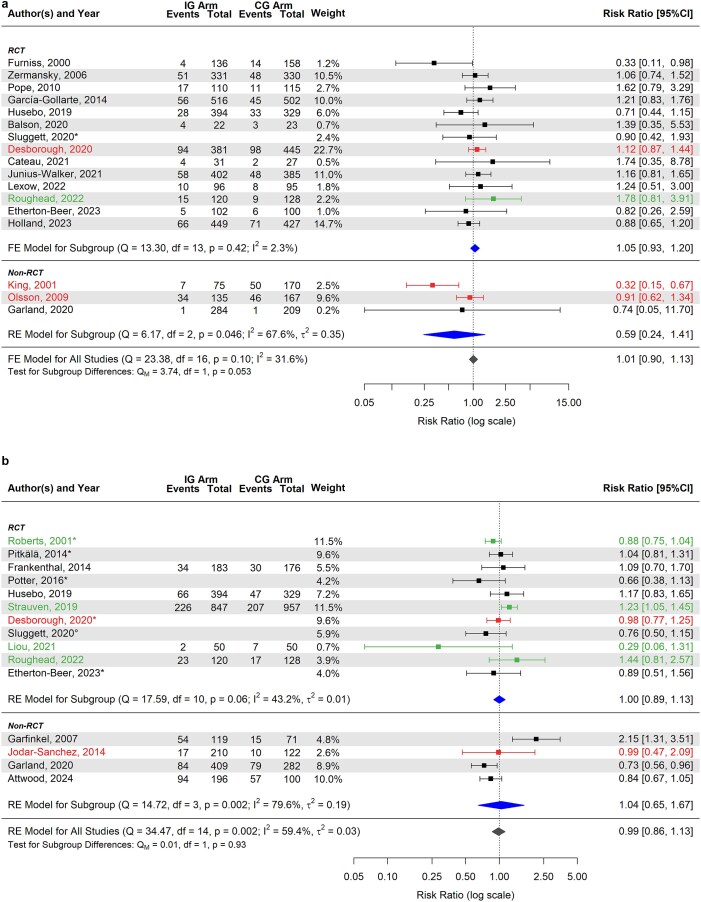
(a) Forest plot of the estimated risk ratio (intervention vs. control group) of death at 1–6 months. (b) Forest plot of the estimated risk ratio (intervention vs. control group) of death at 9–24 months. ^*^Risk ratio was estimated indirectly from the published hazard ratio, using established conversion formulae, and its variance was calculated using the delta method. The colours used for the horizontal lines indicate the type of intervention applied within each study: black for technology- or guideline-based interventions; green for training-based interventions; and red for no specified intervention tools. Estimates are also stratified according to the study type (subgroup). The summary polygon below each subgroup (filled in blue) shows the results of a fixed or random-effects model for just the studies within that group. The summary polygon at the bottom of the plot (filled in dark grey) shows the results of the model when all studies are analysed. The test for subgroup difference (Q_M_) reflects the statistical significance of the subgroup covariate when included in a meta-regression model. Abbreviations: IG, intervention group; CG, control group; RCT, randomised clinical trials; FE, fixed-effects; RE, random-effects; Q, Cochran’s Q statistic, along with degrees of freedom (df) and its *P*-value; I^2^, inconsistency measure; τ^2^, between-study variance.

Overall, 33 studies (86.8%) [31–36, 39–74, 76–78, 81–94, 96–100] assessed the effect of medication review and deprescribing on mortality risk. Among these, 26 (78.8%) [31, 35, 36, 39–74, 78, 83–85, 88–92, 96–100] were included in the meta-analysis, which showed no statistically significant reduction in the risk of death for patients in the intervention group at both 1–6 months (RR 1.01; 95% CI 0.90, 1.13) (Figure 5a) and 9–24 months of follow-up (RR 0.99; 95% CI 0.86, 1.13) (Figure 5b). The forest plot of the risk of death regardless of the timeframe considered (RR 0.98; 95% CI 0.89, 1.09) is reported in Appendix Figure A4.

### Meta-regression for mortality risk

To explore the sources of between-study heterogeneity concerning mortality risk at 9–24 months, a meta-regression analysis was performed using *a priori* selected study-level covariates (Appendix Table A6). Results of this analysis showed that age at enrolment (R^2^ = −43.5%; *P* = .330) and study design (R^2^ = −19.4%; *P* = .930) did not significantly explain between-study heterogeneity. The geographical region was associated with a considerable yet non-statistically significant reduction in between-study variance (τ^2^ reduced to 0.0145), accounting for 56.2% of the heterogeneity (R^2^). The pooled estimate remained similar in all cases, ranging from 0.99 to 1.01.

### Effect of medication review and deprescribing interventions on other clinical outcomes

Twenty-two studies (57.9%) [31, 35, 37–42, 49–53, 55, 57–74, 76, 77, 79, 84, 85, 88–93, 96, 97, 99, 100] reported measures of physical function, cognitive function or other indicators of quality of life, while 6 (15.8%) [42, 80, 82, 88–92, 95] evaluated the incidence of adverse events/ADRs (Appendix Table A4). In this regard, one study found more severe adverse events in the control group compared to the intervention group [91, 92], one study did not find any ADR related to the deprescription [80], two studies found no statistically significant differences between the two groups [42, 88–90] and two studies did not compute any statistical test for this evaluation [82, 95]. Other clinical outcomes are shown in Appendix Table A4.

### Publication bias assessment

No publication bias was detected concerning the risk of death (Kendall’s tau = 0.01, *P* = 1.00) (Appendix Figure A5). Due to the low number of studies included, publication bias was not evaluated for the other meta-analyses.

### Risk of bias assessment

Of the 22 RCTs included in this systematic review, 1 (4.5%) was considered at ‘high’ risk of bias, in 20 (90.9%) the risk of bias was rated as ‘some concerns’ and 1 (4.5%) as ‘low’ risk (Appendix Figure A6). In most of these studies, the potential biases arose from deviations from intended interventions and in the measurement of the outcome (Appendix Figure A7).

Among the 16 quasi-experimental studies included, the risk of bias was considered ‘critical’ for 5 (31.3%), ‘serious’ for 8 and ‘moderate’ for the remaining 3 studies (18.8%). No study was rated as having a ‘low’ risk of bias (Appendix Figure A8). The potential biases were mainly related to confounding, classification of interventions, deviations from intended interventions and missing data (Appendix Figure A9).

## Discussion

Despite the substantial heterogeneity in methodologies and outcome measures across the included studies, medication review interventions consistently reduced both medication burden and PIM prevalence. This aligns with the meta-analysis of Kua *et al*., which documented the efficacy of targeted deprescribing in lowering PIM use among nursing home residents [22]. However, unlike Kua *et al*., our findings did not demonstrate significant benefits on falls or mortality. Notably, their review included studies that met our exclusion criteria (i.e. one study specifically targeting anticholinergic burden, one study with a composite intervention of antipsychotic review combined with social interaction and exercise and one study including also patients not residing in LTCFs), and their data extraction from Roberts *et al*. [42] and Potter *et al*. [85] differed from ours.

Our review focused on multifaceted interventions, which may offer broader benefits than targeted approaches and also carry the risk of overlooking drug-specific issues (e.g. benzodiazepines [101] or proton pump inhibitors [102]). This risk is particularly relevant when interventions rely solely on clinical expertise without the use of structured tools. Comprehensive evaluation requires the systematic assessment of all domains relevant to prescribing appropriateness, including indication for use, dosage, adherence, risk of DDIs and potential ADRs, anticholinergic burden, prescribing cascades and therapeutic duplication [17]. Our meta-regression analysis suggested that geographical region was the main source of heterogeneity in mortality rates, potentially reflecting the methodological variability and the absence of international standards. Moreover, medication review/deprescribing interventions are not yet uniformly implemented across healthcare systems. Although interest in these interventions in LTCFs has increased in recent years, with most included studies published in the last decade (73.7%), the available evidence remains unevenly distributed geographically. In particular, studies were predominantly conducted in Europe and Oceania, which may limit the generalizability of findings to other healthcare systems.

Clearly describing the methodology used for medication review is essential, and transparent documentation is necessary for replication in RCTs and real-world applications. Unguided expertise-driven interventions not only lack repeatability but also risk lacking the multidimensionality required for effective medication review/deprescribing [17, 103–105]. More importantly, a one-time intervention only, without periodic evaluations over time to accommodate the changing clinical conditions of this frail population (e.g. reassessing patient adherence, discussing inappropriate therapies reintroduced over time and reevaluating the appropriateness of new drugs prescribed by specialists), can limit the clinical benefit on all health indicators [17]. Consequently, future studies should adopt standardised and transparent protocols; in this direction, an Italian Scientific Consortium has recently issued a position statement outlining the essential components of multidimensional medication review (i.e. tools, timing and involved professionals) in various healthcare settings, including LTCFs [17].

In most of the studies included in this systematic review, medication review was conducted by clinical pharmacologists or pharmacists. When adequately trained, these healthcare professionals can improve the methodological quality of medication review and deprescribing processes [106, 107]. The role of the clinical pharmacologist/pharmacists may be crucial not only to assess pharmacological therapies but also to train other healthcare professionals involved in the medication review service; on the other hand, clinical pharmacologists/pharmacists may coordinate the development of internal clinical deprescribing protocols. As a pragmatic example, in 2021, Cateau and colleagues conducted an RCT to evaluate the effects of the Quality Circle Deprescribing Module (QC-DeMo) intervention in Swiss nursing homes, aiming to reduce the use of PIMs and improve patient outcomes through a collaborative approach involving physicians, nurses and pharmacists [65–67]. The intervention focused on creating a local deprescribing consensus for specific PIM classes, which was then implemented in the participating nursing homes, and a notable trend towards the reduction of the number of PIMs, hospitalisations and mortality was observed.

The sustainability of medication review interventions beyond research timelines is a crucial issue, as these services are not routinely integrated into standard care in many healthcare systems (e.g. the Italian National Health System). Dedicated multidisciplinary teams providing pharmacological consultations may facilitate the delivery of medication review interventions and contribute to more efficient clinical workflows by redistributing time-consuming medication-related assessments currently undertaken by physicians and nurses [108]. However, the long-term sustainability of such interventions requires adequate economic resources and the allocation of dedicated personnel.

### Strengths and limitations

A major strength of this review is the comprehensive search strategy across three bibliographic databases up to August 2024. To gather more comprehensive information on the real-world state of the art of medication review interventions in LTCFs, both RCTs and quasi-experimental studies were included. The inclusion of the latter can also provide a larger pool of data for meta-analysis without compromising quality (in RCTs, we found a high prevalence of contamination, allocation, performance and detection biases). Additionally, while previous systematic reviews focused on targeted deprescribing, we specifically focused on multidimensional interventions, providing evidence of a more holistic and patient-centred approach closely aligning with clinical practice.

However, some limitations should also be acknowledged. First, most studies had high or moderate risk of bias, warranting cautious interpretation of the findings. Many studies were susceptible to educational and performance biases due to lack of blinding, potentially underestimating intervention effects. To mitigate such biases, future research should prioritize more robust study designs. These include pragmatic RCTs with effective blinding procedures (adjunctive historical control cohorts could also be informative) to minimise contamination within clinical settings. Where randomisation at the individual level is not feasible, alternative designs such as stepped-wedge cluster RCTs or rigorously analysed controlled before-and-after studies are recommended, to better account for confounding factors and temporal trends. Importantly, tools used for intervention delivery should be objective, repeatable and transparently detailed in the publication’s study methods [17, 105]. Second, the substantial between-study heterogeneity found in our meta-analyses underscores the challenges in conducting research in this field and the complexity of generalising clinical outcomes. Finally, we focused our analysis of clinical outcomes on falls, hospitalisation and mortality. Evaluating these relatively rare and negative outcomes in frail older adults requires accounting for multiple factors beyond pharmacotherapy, including progression of comorbidities, caregiver presence and the intensity of nursing care and physiotherapy support in LTCFs [10, 109]. Moreover, patient perspectives and patient-reported outcomes (e.g. symptom burden, functional status) represent an important dimension of medication review and deprescribing [17]. In this study, we did not include qualitative research and, although we descriptively reported some measures where available (e.g. quality of life scales), we did not perform a dedicated analysis. Future reviews could specifically address these aspects to better capture acceptability, perceived benefits and the effects on quality of life.

## Conclusion

This systematic review and meta-analysis showed that medication review and deprescribing improve prescribing appropriateness in LTCFs but did not significantly affect clinical outcomes (i.e. falls, hospitalisations and mortality), likely due to heterogeneity in study designs and intervention components. Further research with standardised, objective and reproducible methodologies is needed to better define their clinical impact.

## Supplementary Material

afag084_Supplemental_File
