# Supplementary material for: Evaluating the impact of medication review and deprescribing on prescribing appropriateness and clinical outcomes in older people residing in long-term care facilities: a systematic review and meta-analysis
Source: Age Ageing. 2026 Apr 12;55(4):afag084. doi: 10.1093/ageing/afag084 (PMC13071406; doi:10.1093/ageing/afag084)
Supplement: afag084_Supplemental_File [file afag084_supplemental_file.docx]

**APPENDIX**

**Clinical Impact of Medication Review and Deprescribing in Older People Residing in Long-term Care Facilities: A Systematic Review and Meta-analysis of Clinical Trials and Observational Studies**

**Authors:**

Massimo Carollo, MD^1#^, Irene Cristini, MSc^1#^, Salvatore Crisafulli, PhD^1^, Andrea Fontana, MSc^2^, Anna Forti, MD^1^, Aurora Lanaro, MSc^1^, Francesco Maccarrone, MSc^1^, Marta Zerio, MSc^1^, Luca Piccoli, MSc^1^, Elisabetta Poluzzi, PhD^3^, Graziano Onder, PhD^4,5^, Gianluca Trifirò, PhD^1^*

# Massimo Carollo and Irene Cristini are equal contributors.

**Affiliations:**

1. Department of Diagnostics and Public Health, University of Verona, Verona, Italy
2. Unit of Biostatistics, IRCCS Casa Sollievo della Sofferenza, Foggia, Italy
3. Department of Medical and Surgical Sciences, University of Bologna, Bologna, Italy
4. Department of Geriatrics, Orthopedics and Rheumatology, Università Cattolica del Sacro Cuore, Rome, Italy
5. Center of Aging, Fondazione Policlinico Universitario Gemelli IRCCS, Rome, Italy

***Corresponding author**

Prof. Gianluca Trifirò

gianluca.trifiro@univr.it

Department of Diagnostics and Public Health, University of Verona, Italy

P.le L.A. Scuro 10, 37124, Verona

+39 045 812 4706

**Table of contents**

[**Table A1**. Search strategy. 3](#_Toc223962008)

[**Table A2**. Included reports in the systematic review. 6](#_Toc223962009)

[**Table A3**. Excluded reports after full-text assessment for eligibility. 12](#_Toc223962010)

[**Table A4**. Characteristics of the studies included in the systematic review. 19](#_Toc223962011)

[**Table A5.** Tools used to conduct medication review interventions. 39](#_Toc223962012)

[**Table A6.** Results from meta-regression and assessment of residual heterogeneity for mortality risk at 9-24 months. 41](#_Toc223962013)

[**Figure A1**. Forest plot of the estimated mean differences (intervention minus control group) of falls per patient at 6 months. 42](#_Toc223962014)

[**Figure A2**. Forest plot of the estimated mean differences (intervention minus control group) of falls per patient at 12 months. 43](#_Toc223962015)

[**Figure A3**. Forest plot of the estimated mean differences (intervention minus control group) of hospitalizations per patient at 6 or 12 months. 44](#_Toc223962016)

[**Figure A4**. Forest plot of the estimated risk ratio (intervention vs. control group) of death evaluated at the last available follow-up. 45](#_Toc223962017)

[**Figure A5**. Funnel plot of risk ratio of death at 9-24 months with estimated Kendall's tau coefficient to test for asymmetry. 46](#_Toc223962018)

[**Figure A6.** Risk of bias of the randomized controlled trials included in the systematic review. 47](#_Toc223962019)

[**Figure A7**. Summary plot of the risk of bias of the randomized controlled trials included in the systematic review. 48](#_Toc223962020)

[**Figure A8.** Risk of bias of the non-randomized studies included in the systematic review. 49](#_Toc223962021)

[**Figure A9**. Summary plot of the risk of bias of the non-randomized studies included in the systematic review. 50](#_Toc223962022)

[**Statistical Methods** 51](#_Toc223962023)

**Table A1**. Search strategy.

| **PubMed** | | |
| --- | --- | --- |
| **Search number** | **Query** | **Results** |
| #1 | "old patients"[Title/Abstract] OR "older"[Title/Abstract] OR "aged"[Title] OR "aged"[MeSH Terms] OR "elder*"[Title/Abstract] OR "geriatr*"[Title/Abstract] OR "geriatrics"[MeSH Terms] | 3,998,332 |
| #2 | "nursing home*"[Title/Abstract] OR "long term care facilit*"[Title/Abstract] OR "long term care facilit*"[Title/Abstract] OR "care home*"[Title/Abstract] OR "elderly care facilit*"[Title/Abstract] OR "assisted living facilit*"[Title/Abstract] OR "residential aged care"[Title/Abstract] OR "senior living residen*"[Title/Abstract] OR "residential care home*"[Title/Abstract] OR "nursing facilit*"[Title/Abstract] OR "retirement home*"[Title/Abstract] OR "Nursing Homes"[MeSH Terms] OR "Residential Facilities"[MeSH Terms] OR "homes for the aged"[MeSH Terms] | 83,771 |
| #3 | #1 AND #2 | 51,680 |
| #4 | ("reduc*"[Title] OR "ceas*"[Title] OR "stop*"[Title] OR "withdraw*"[Title] OR "discontin*"[Title]) AND ("Potentially inappropriate medications"[Title] OR "drug*"[Title] OR "medic*"[Title] OR "polypharmacy"[Title] OR "polytherapy"[Title] OR "Polypharmacotherapy"[Title]) | 15,233 |
| #5 | ("optimiz*"[Title] OR "improv*"[Title] OR "review*"[Title]) AND ("Potentially inappropriate medications"[Title] OR "polypharmacy"[Title] OR "polytherapy"[Title] OR "Polypharmacotherapy"[Title]) | 365 |
| #6 | "deprescribe"[Title/Abstract] OR "deprescribed"[Title/Abstract] OR "deprescriptions"[Title/Abstract] OR "deprescribing"[Title/Abstract] OR "deprescriptions"[MeSH Terms] OR "deprescription*"[Title/Abstract] OR "inappropriate prescribing"[MeSH Terms] OR "potentially inappropriate medication list"[MeSH Terms] OR "Beers criteria"[Title/Abstract] OR "medication review*"[Title/Abstract] OR "medication review"[MeSH Terms] | 9,962 |
| #7 | #4 OR #5 OR #6 | 25,017 |
| #8 | #3 AND #7 | 951 |
| **Scopus** | | |
| **Search number** | **Query** | **Results** |
| #1 | TITLE-ABS-KEY("old patients") OR TITLE-ABS("older") OR TITLE("aged") OR TITLE-ABS("elder*") OR TITLE-ABS-KEY("geriatr*") | 1,427,511 |
| #2 | TITLE-ABS-KEY("nursing home*") OR TITLE-ABS-KEY("long-term care facilit*") OR TITLE-ABS-KEY("long term care facilit*") OR TITLE-ABS-KEY("care home*") OR TITLE-ABS-KEY("elderly care facilit*") OR TITLE-ABS-KEY("assisted living facilit*") OR TITLE-ABS-KEY("residential aged care") OR TITLE-ABS-KEY("senior living residen*") OR TITLE-ABS-KEY("residential care home*") OR TITLE-ABS-KEY("nursing facilit*") OR TITLE-ABS-KEY("retirement home*") OR TITLE-ABS-KEY("Residential Facilit*") OR TITLE-ABS-KEY("homes for the aged") | 109,835 |
| #3 | #1 AND #2 | 45,410 |
| #4 | (TITLE("reduc*") OR TITLE("ceas*") OR TITLE("stop*") OR TITLE("withdraw*") OR TITLE("discontin*")) AND (TITLE("Potentially inappropriate medications") OR TITLE("drug*") OR TITLE("medic*") OR TITLE("polypharmacy") OR TITLE("polytherapy") OR TITLE("Polypharmacotherapy")) | 19,687 |
| #5 | (TITLE("optimiz*") OR TITLE("improv*")) AND (TITLE("Potentially inappropriate medications") OR TITLE("polypharmacy") OR TITLE("polytherapy") OR TITLE("Polypharmacotherapy")) | 128 |
| #6 | TITLE-ABS-KEY("deprescrib*") OR TITLE-ABS-KEY("inappropriate prescribing") OR TITLE-ABS("Beers criteria") OR TITLE-ABS-KEY("medication review*") | 13,291 |
| #7 | #4 OR #5 OR #6 | 32,637 |
| #8 | #3 AND #7 | 896 |
| **Embase** | | |
| **Search number** | **Query** | **Results** |
| #1 | 'old patients':ti,ab,kw OR 'older':ti,ab,kw OR 'aged':ti OR 'aged'/exp OR 'elder*':ti,ab,kw OR 'geriatr*':ti,ab,kw OR 'geriatrics'/exp | 6,672,185 |
| #2 | 'nursing home*':ti,ab,kw OR 'long term care facilit*':ti,ab,kw OR 'long term care facilit*':ti,ab,kw OR 'care home*':ti,ab,kw OR 'elderly care facilit*':ti,ab,kw OR 'assisted living facilit*':ti,ab,kw OR 'residential aged care':ti,ab,kw OR 'senior living residen*':ti,ab,kw OR 'residential care home*':ti,ab,kw OR 'nursing facilit*':ti,ab,kw OR 'retirement home*':ti,ab,kw OR 'nursing home'/exp OR 'residential home'/exp OR 'home for the aged'/exp | 109,002 |
| #3 | #1 AND #2 | 70,520 |
| #4 | ('reduc*':ti OR 'ceas*':ti OR 'stop*':ti OR 'withdraw*':ti OR 'discontin*':ti) AND ('potentially inappropriate medications':ti OR 'drug*':ti OR 'medic*':ti OR 'polypharmacy':ti OR 'polytherapy':ti OR 'polypharmacotherapy':ti) | 21,208 |
| #5 | ('optimiz*':ti OR 'improv*':ti OR 'review*':ti) AND ('potentially inappropriate medications':ti OR 'polypharmacy':ti OR 'polytherapy':ti OR 'polypharmacotherapy':ti) | 521 |
| #6 | 'deprescribe':ti,ab,kw OR 'deprescribed':ti,ab,kw OR 'deprescriptions':ti,ab,kw OR 'deprescribing':ti,ab,kw OR 'deprescription'/exp OR 'deprescription*':ti,ab,kw OR 'prescribing error'/exp OR 'potentially inappropriate medication'/exp OR 'beers criteria':ti,ab,kw OR 'medication review*':ti,ab,kw OR 'drug utilization review'/exp | 31,429 |
| #7 | #4 OR #5 OR #6 | 52,247 |
| #8 | #3 AND #7 | 1,696 |

**Table A2**. Included reports in the systematic review.

| **Study** | **References** |
| --- | --- |
| Attwood, 2024 | Attwood D, Vafidis J, Boorer J, et al. IT-assisted comprehensive geriatric assessment for residents in care homes: quasi-experimental longitudinal study. BMC Geriatr. 2024;24(1):269. Published 2024 Mar 19. doi:10.1186/s12877-024-04824-6 |
| Balson, 2020 | Balsom C, Pittman N, King R, Kelly D. Impact of a pharmacist-administered deprescribing intervention on nursing home residents: a randomized controlled trial. Int J Clin Pharm. 2020;42(4):1153-1167. doi:10.1007/s11096-020-01073-6 |
| Baqir, 2014 | Baqir W, Barrett S, Desai N, Copeland R, Hughes J. A clinico-ethical framework for multidisciplinary review of medication in nursing homes. BMJ Qual Improv Rep. 2014;3(1):u203261.w2538. Published 2014 Dec 10. doi:10.1136/bmjquality.u203261.w2538 |
| Cateau, 2021 | Cateau D, Ballabeni P, Mena S, Bugnon O, Niquille A. Deprescribing in nursing homes: Protocol for nested, randomised controlled hybrid trials of deprescribing interventions. Res Social Adm Pharm. 2021;17(4):786-794. doi:10.1016/j.sapharm.2020.05.026 |
|  | Cateau D, Ballabeni P, Niquille A. Effects of an interprofessional Quality Circle-Deprescribing Module (QC-DeMo) in Swiss nursing homes: a randomised controlled trial. BMC Geriatr. 2021;21(1):289. Published 2021 May 1. doi:10.1186/s12877-021-02220-y |
|  | Cateau D, Ballabeni P, Niquille A. Effects of an interprofessional deprescribing intervention in Swiss nursing homes: the Individual Deprescribing Intervention (IDeI) randomised controlled trial. BMC Geriatr. 2021;21(1):655. Published 2021 Nov 19. doi:10.1186/s12877-021-02465-7 |
| Chan, 2024 | Chan J, Bolitho R, Hay K, Yong F. A pre-post study of pharmacist-led medication reviews within a hospital-based residential aged care support service. Int J Pharm Pract. 2024;32(4):303-310. doi:10.1093/ijpp/riae018 |
| Dalin, 2022 | Dalin DA, Frandsen S, Madsen GK, Vermehren C. Exploration of Symptom Scale as an Outcome for Deprescribing: A Medication Review Study in Nursing Homes. Pharmaceuticals (Basel). 2022;15(5):505. Published 2022 Apr 21. doi:10.3390/ph15050505 |
| Desborough, 2020 | Desborough J, Houghton J, Wood J, et al. Multi-professional clinical medication reviews in care homes for the elderly: study protocol for a randomised controlled trial with cost effectiveness analysis. *Trials*. 2011;12:218. Published 2011 Oct 5. doi:10.1186/1745-6215-12-218 |
|  | Desborough JA, Clark A, Houghton J, et al. Clinical and cost effectiveness of a multi-professional medication reviews in care homes (CAREMED). Int J Pharm Pract. 2020;28(6):626-634. doi:10.1111/ijpp.12656 |
| Etherton-Beer, 2023 | Quek HW, Etherton-Beer C, Page A, et al. Deprescribing for older people living in residential aged care facilities: Pharmacist recommendations, doctor acceptance and implementation. Arch Gerontol Geriatr. 2023;107:104910. doi:10.1016/j.archger.2022.104910 |
|  | Etherton-Beer C, Page A, Naganathan V, et al. Deprescribing to optimise health outcomes for frail older people: a double-blind placebo-controlled randomised controlled trial-outcomes of the Opti-med study. Age Ageing. 2023;52(5):afad081. doi:10.1093/ageing/afad081 |
| Frankenthal, 2014 | Frankenthal D, Lerman Y, Kalendaryev E, Lerman Y. Intervention with the screening tool of older persons potentially inappropriate prescriptions/screening tool to alert doctors to right treatment criteria in elderly residents of a chronic geriatric facility: a randomized clinical trial. J Am Geriatr Soc. 2014;62(9):1658-1665. doi:10.1111/jgs.12993 |
|  | Frankenthal D, Lerman Y, Kalendaryev E, Lerman Y. Potentially inappropriate prescribing among older residents in a geriatric hospital in Israel. *Int J Clin Pharm*. 2013;35(5):677-682. doi:10.1007/s11096-013-9790-z |
|  | Frankenthal D, Israeli A, Caraco Y, et al. Long-Term Outcomes of Medication Intervention Using the Screening Tool of Older Persons Potentially Inappropriate Prescriptions Screening Tool to Alert Doctors to Right Treatment Criteria. J Am Geriatr Soc. 2017;65(2):e33-e38. doi:10.1111/jgs.14570 |
| Furniss, 2000 | Furniss L, Burns A, Craig SK, Scobie S, Cooke J, Faragher B. Effects of a pharmacist's medication review in nursing homes. Randomised controlled trial. Br J Psychiatry. 2000;176:563-567. doi:10.1192/bjp.176.6.563 |
|  | Burns A, Furniss L, Cooke J, Lloyd Craig SK, Scobie S. Pharmacist medication review in nursing homes: a cost analysis. Int J Geriatr Psychopharmacol. 2000;2(3):137e141 |
| García-Gollarte, 2014 | García-Gollarte F, Baleriola-Júlvez J, Ferrero-López I, Cuenllas-Díaz Á, Cruz-Jentoft AJ. An educational intervention on drug use in nursing homes improves health outcomes resource utilization and reduces inappropriate drug prescription. *J Am Med Dir Assoc*. 2014;15(12):885-891. doi:10.1016/j.jamda.2014.04.010 |
| Garfinkel, 2007 | Garfinkel D, Zur-Gil S, Ben-Israel J. The war against polypharmacy: a new cost-effective geriatric-palliative approach for improving drug therapy in disabled elderly people. Isr Med Assoc J. 2007;9(6):430-434. |
| Garland, 2020 | Garland CT, Guénette L, Kröger E, Carmichael PH, Rouleau R, Sirois C. A New Care Model Reduces Polypharmacy and Potentially Inappropriate Medications in Long-Term Care. J Am Med Dir Assoc. 2021;22(1):141-147. doi:10.1016/j.jamda.2020.09.039 |
| Gaubert-Dahan, 2019 | Gaubert-Dahan ML, Sebouai A, Tourid W, Fauvelle F, Aikpa R, Bonnet-Zamponi D. The impact of medication review with version 2 STOPP (Screening Tool of Older Person's Prescriptions) and START (Screening Tool to Alert doctors to Right Treatment) criteria in a French nursing home: a 3-month follow-up study. Ther Adv Drug Saf. 2019;10:2042098619855535. Published 2019 Jun 9. doi:10.1177/2042098619855535 |
| Hashimoto, 2019 | Hashimoto R, Fujii K, Shimoji S, et al. Study of pharmacist intervention in polypharmacy among older patients: Non-randomized, controlled trial. Geriatr Gerontol Int. 2020;20(3):229-237. doi:10.1111/ggi.13850 |
| Holland, 2023 | Bond CM, Holland R, Alldred DP, et al. Protocol for a cluster randomised controlled trial to determine the effectiveness and cost-effectiveness of independent pharmacist prescribing in care homes: the CHIPPS study. Trials. 2020;21(1):103. Published 2020 Jan 21. doi:10.1186/s13063-019-3827-0 |
|  | Bond CM, Holland R, Alldred DP, et al. Protocol for the process evaluation of a cluster randomised controlled trial to determine the effectiveness and cost-effectiveness of independent pharmacist prescribing in care home: the CHIPPS study. Trials. 2020;21(1):439. Published 2020 May 29. doi:10.1186/s13063-020-04264-8 |
|  | Birt L, Dalgarno L, Wright DJ, et al. Process evaluation for the Care Homes Independent Pharmacist Prescriber Study (CHIPPS). BMC Health Serv Res. 2021;21(1):1041. Published 2021 Oct 2. doi:10.1186/s12913-021-07062-3 |
|  | Holland R, Bond C, Alldred DP, et al. Evaluation of effectiveness and safety of pharmacist independent prescribers in care homes: cluster randomised controlled trial [published correction appears in BMJ. 2023 Feb 23;380:p446. doi: 10.1136/bmj.p446]. BMJ. 2023;380:e071883. Published 2023 Feb 14. doi:10.1136/bmj-2022-071883 |
|  | Wright D, Holland R, Alldred DP, et al. The Care Home Independent Pharmacist Prescriber Study (CHIPPS): development and implementation of an RCT to estimate safety, effectiveness and cost-effectiveness. Southampton (UK): National Institute for Health and Care Research; December 2023. |
| Husebo, 2019 | Husebo BS, Flo E, Aarsland D, et al. COSMOS--improving the quality of life in nursing home patients: protocol for an effectiveness-implementation cluster randomized clinical hybrid trial. Implement Sci. 2015;10:131. Published 2015 Sep 15. doi:10.1186/s13012-015-0310-5 |
|  | Aasmul I, Husebo BS, Flo E. Description of an advance care planning intervention in nursing homes: outcomes of the process evaluation. BMC Geriatr. 2018;18(1):26. Published 2018 Jan 25. doi:10.1186/s12877-018-0713-7 |
|  | Husebø BS, Ballard C, Aarsland D, et al. The Effect of a Multicomponent Intervention on Quality of Life in Residents of Nursing Homes: A Randomized Controlled Trial (COSMOS). J Am Med Dir Assoc. 2019;20(3):330-339. doi:10.1016/j.jamda.2018.11.006 |
|  | Gulla C, Flo E, Kjome RLS, Husebo BS. Implementing a novel strategy for interprofessional medication review using collegial mentoring and systematic clinical evaluation in nursing homes (COSMOS). BMC Geriatr. 2019;19(1):130. Published 2019 May 7. doi:10.1186/s12877-019-1139-6 |
| Jodar-Sanchez, 2014 | Jódar-Sánchez F, Martín JJ, López del Amo MP, García L, Araújo-Santos JM, Epstein D. Cost-utility analysis of a pharmacotherapy follow-up for elderly nursing home residents in Spain. J Am Geriatr Soc. 2014;62(7):1272-1280. doi:10.1111/jgs.12890 |
| Junius-Walker, 2021 | Krause O, Wiese B, Doyle IM, et al. Multidisciplinary intervention to improve medication safety in nursing home residents: protocol of a cluster randomised controlled trial (HIOPP-3-iTBX study). BMC Geriatr. 2019;19(1):24. Published 2019 Jan 25. doi:10.1186/s12877-019-1027-0 |
|  | Junius-Walker U, Krause O, Thürmann P, et al. Drug Safety for Nursing-Home Residents-Findings of a Pragmatic, Cluster-Randomized, Controlled Intervention Trialin 44 Nursing Homes. Dtsch Arztebl Int. 2021;118(42):705-712. doi:10.3238/arztebl.m2021.0297 |
| King, 2001 | King MA, Roberts MS. Multidisciplinary case conference reviews: improving outcomes for nursing home residents, carers and health professionals. Pharm World Sci. 2001;23(2):41-45. doi:10.1023/a:1011215008000 |
| Kua, 2020 | Kua CH, Yeo CYY, Char CWT, et al. Nursing home team-care deprescribing study: a stepped-wedge randomised controlled trial protocol. BMJ Open. 2017;7(5):e015293. Published 2017 May 9. doi:10.1136/bmjopen-2016-015293 |
|  | Kua CH, Yeo CYY, Tan PC, et al. Association of Deprescribing With Reduction in Mortality and Hospitalization: A Pragmatic Stepped-Wedge Cluster-Randomized Controlled Trial. J Am Med Dir Assoc. 2021;22(1):82-89.e3. doi:10.1016/j.jamda.2020.03.012 |
| Lapane, 2006 | Cameron K, Feinberg JL, Lapane KL. Fleetwood Project Phase III moves  forward. Consult Pharm 2002;17:180–200 |
|  | Lapane KL, Hughes CM. Pharmacotherapy interventions undertaken by pharmacists in the Fleetwood phase III study: the role of process control. Ann Pharmacother. 2006;40(9):1522-1526. doi:10.1345/aph.1G702 |
|  | Lapane KL, Hughes CM, Christian JB, Daiello LA, Cameron KA, Feinberg J. Evaluation of the fleetwood model of long-term care pharmacy. J Am Med Dir Assoc. 2011;12(5):355-363. doi:10.1016/j.jamda.2010.03.003 |
| Lexow, 2022 | Lexow M, Wernecke K, Sultzer R, Bertsche T, Schiek S. Determine the impact of a structured pharmacist-led medication review - a controlled intervention study to optimise medication safety for residents in long-term care facilities. BMC Geriatr. 2022;22(1):307. Published 2022 Apr 9. doi:10.1186/s12877-022-03025-3 |
| Liou, 2021 | Liou WS, Huang SM, Lee WH, Chang YL, Wu MF. The effects of a pharmacist-led medication review in a nursing home: A randomized controlled trial. Medicine (Baltimore). 2021;100(48):e28023. doi:10.1097/MD.0000000000028023 |
| Mahlknecht, 2019 | Mahlknecht A, Nestler N, Bauer U, et al. Effect of training and structured medication review on medication appropriateness in nursing home residents and on cooperation between health care professionals: the InTherAKT study protocol. BMC Geriatr. 2017;17(1):24. Published 2017 Jan 18. doi:10.1186/s12877-017-0418-3 |
|  | Mahlknecht A, Krisch L, Nestler N, et al. Impact of training and structured medication review on medication appropriateness and patient-related outcomes in nursing homes: results from the interventional study InTherAKT. BMC Geriatr. 2019;19(1):257. Published 2019 Sep 18. doi:10.1186/s12877-019-1263-3 |
| Olsson, 2009 | Olsson IN, Curman B, Engfeldt P. Patient focused drug surveillance of elderly patients in nursing homes. Pharmacoepidemiol Drug Saf. 2010;19(2):150-157. doi:10.1002/pds.1891 |
| Pitkälä, 2014 | Pitkala KH, Juola AL, Soini H, et al. Reducing inappropriate, anticholinergic and psychotropic drugs among older residents in assisted living facilities: study protocol for a randomized controlled trial. *Trials*. 2012;13:85. Published 2012 Jun 18. doi:10.1186/1745-6215-13-85 |
|  | Pitkälä KH, Juola AL, Kautiainen H, et al. Education to reduce potentially harmful medication use among residents of assisted living facilities: a randomized controlled trial. *J Am Med Dir Assoc*. 2014;15(12):892-898. doi:10.1016/j.jamda.2014.04.002 |
|  | Juola AL, Bjorkman MP, Pylkkanen S, et al. Feasibility and baseline findings of an educational intervention in a randomized trial to optimize drug treatment among residents in assisted living facilities. European Geriatric Medicine. 2014;5(3):195-199. doi:10.1016/j.eurger.2014.02.005 |
|  | Juola AL, Bjorkman MP, Pylkkanen S, et al. Nurse Education to Reduce Harmful Medication Use in Assisted Living Facilities: Effects of a Randomized Controlled Trial on Falls and Cognition. Drugs Aging. 2015;32(11):947-955. doi:10.1007/s40266-015-0311-8 |
| Pope, 2010 | Pope G, Wall N, Peters CM, et al. Specialist medication review does not benefit short-term outcomes and net costs in continuing-care patients. Age Ageing. 2011;40(3):307-312. doi:10.1093/ageing/afq095 |
| Potter, 2016 | Potter K, Flicker L, Page A, Etherton-Beer C. Deprescribing in Frail Older People: A Randomised Controlled Trial. PLoS One. 2016;11(3):e0149984. Published 2016 Mar 4. doi:10.1371/journal.pone.0149984 |
| Pruskowski, 2017 | Pruskowski J, Handler SM. The DE-PHARM Project: A Pharmacist-Driven Deprescribing Initiative in a Nursing Facility. Consult Pharm. 2017;32(8):468-478. doi:10.4140/TCP.n.2017.468 |
| Roberts, 2001 | Roberts MS, Stokes JA, King MA, et al. Outcomes of a randomized controlled trial of a clinical pharmacy intervention in 52 nursing homes. Br J Clin Pharmacol. 2001;51(3):257-265. doi:10.1046/j.1365-2125.2001.00347.x |
| Roughead, 2022 | Lim R, Bereznicki L, Corlis M, et al. Reducing medicine-induced deterioration and adverse reactions (ReMInDAR) trial: study protocol for a randomised controlled trial in residential aged-care facilities assessing frailty as the primary outcome. BMJ Open. 2020;10(4):e032851. Published 2020 Apr 22. doi:10.1136/bmjopen-2019-032851 |
|  | Roughead EE, Pratt NL, Parfitt G, et al. Effect of an ongoing pharmacist service to reduce medicine-induced deterioration and adverse reactions in aged-care facilities (nursing homes): a multicentre, randomised controlled trial (the ReMInDAR trial). Age Ageing. 2022;51(4):afac092. doi:10.1093/ageing/afac092 |
|  | Dorj G, Nair NP, Bereznicki L, et al. Risk factors predictive of adverse drug events and drug-related falls in aged care residents: secondary analysis from the ReMInDAR trial [published correction appears in Drugs Aging. 2023 Jan;40(1):89. doi: 10.1007/s40266-022-00996-1]. Drugs Aging. 2023;40(1):49-58. doi:10.1007/s40266-022-00983-6 |
|  | Kalisch Ellett LM, Dorj G, Andrade AQ, et al. Prevalence and Preventability of Adverse Medicine Events in a Sample of Australian Aged-Care Residents: A Secondary Analysis of Data from the ReMInDAR Trial. Drug Saf. 2023;46(5):493-500. doi:10.1007/s40264-023-01299-z |
| Sankaran, 2010 | Sankaran S, Kenealy T, Adair A, et al. A complex intervention to support 'rest home' care: a pilot study. N Z Med J. 2010;123(1308):41-53. Published 2010 Jan 29 |
| Sanz-Tamargo, 2019 | Sanz-Tamargo G, García-Cases S, Navarro A, Lumbreras B. Adaptation of a deprescription intervention to the medication management of older people living in long-term care facilities. Expert Opin Drug Saf. 2019;18(11):1091-1098. doi:10.1080/14740338.2019.1667330 |
| Schmidt, 1998 | Schmidt IK, Claesson CB, Westerholm B, Nilsson LG. Physician and staff assessments of drug interventions and outcomes in Swedish nursing homes. Ann Pharmacother. 1998;32(1):27-32. doi:10.1177/106002809803200102 |
|  | Claesson CB, Schmidt IK. Drug use in Swedish nursing homes. Clin Drug Investig. 1998;16(6):441-452. doi:10.2165/00044011-199816060-00004 |
| Sluggett, 2020 | Sluggett JK, Chen EYH, Ilomäki J, et al. SImpliﬁcation of Medications Prescribed  to Long-tErm care Residents (SIMPLER): Study protocol for a cluster random-  ized controlled trial. Trials 2018;19:37. |
|  | Sluggett JK, Chen EYH, Ilomäki J, et al. Reducing the Burden of Complex Medication Regimens: SImplification of Medications Prescribed to Long-tErm care Residents (SIMPLER) Cluster Randomized Controlled Trial. J Am Med Dir Assoc. 2020;21(8):1114-1120.e4. doi:10.1016/j.jamda.2020.02.003 |
|  | Sluggett JK, Hopkins RE, Chen EY, et al. Impact of Medication Regimen Simplification on Medication Administration Times and Health Outcomes in Residential Aged Care: 12 Month Follow Up of the SIMPLER Randomized Controlled Trial. J Clin Med. 2020;9(4):1053. Published 2020 Apr 8. doi:10.3390/jcm9041053 |
| Strauven, 2019 | Anrys P, Strauven G, Boland B, et al. Collaborative approach to Optimise MEdication use for Older people in Nursing homes (COME-ON): study protocol of a cluster controlled trial. Implement Sci. 2016;11:35. Published 2016 Mar 11. doi:10.1186/s13012-016-0394-6 |
|  | Strauven G, Anrys P, Vandael E, et al. Cluster-Controlled Trial of an Intervention to Improve Prescribing in Nursing Homes Study. J Am Med Dir Assoc. 2019;20(11):1404-1411. doi:10.1016/j.jamda.2019.06.006 |
|  | Anrys P, Strauven G, Roussel S, et al. Process evaluation of a complex intervention to optimize quality of prescribing in nursing homes (COME-ON study). Implement Sci. 2019;14(1):104. Published 2019 Dec 11. doi:10.1186/s13012-019-0945-8 |
|  | Fournier A, Anrys P, Beuscart JB, et al. Use and Deprescribing of Potentially Inappropriate Medications in Frail Nursing Home Residents. Drugs Aging. 2020;37(12):917-924. doi:10.1007/s40266-020-00805-7 |
| Zermansky, 2006 | Zermansky AG, Alldred DP, Petty DR, et al. Clinical medication review by a pharmacist of elderly people living in care homes--randomised controlled trial. *Age Ageing*. 2006;35(6):586-591. doi:10.1093/ageing/afl075 |
|  | Alldred DP, Zermansky AG, Petty DR, et al. Clinical medication review by a pharmacist of elderly people living in care homes: pharmacist interventions. Int J Pharm Pract. 2007;15(2):93-99. doi:10.1211/ijpp.15.2.0003. |

**Table A3**. Excluded reports after full-text assessment for eligibility.

| **Study** | **Reference** | **Reason(s) for exclusion** |
| --- | --- | --- |
| Al Aqqad, 2014 | Al Aqqad SM, Chen LL, Shafie AA, Hassali MA, Tangiisuran B. The use of potentially inappropriate medications and changes in quality of life among older nursing home residents. Clin Interv Aging. 2014;9:201-207. Published 2014 Jan 22. doi:10.2147/CIA.S52356 | No intervention was performed. |
| Alves, 2016 | Alves A, James DH, Green S. Deprescribing of medicines in care homes—Primary care pharmacists’ practices and perspectives. Int J Pharm Pract. 2016;24(Suppl S3):37–38 | Conference abstract. |
| Alves, 2019 | Alves A, Green S, James DH. Deprescribing of Medicines in Care Homes-A Five-Year Evaluation of Primary Care Pharmacist Practices. Pharmacy (Basel). 2019;7(3):105. Published 2019 Aug 3. doi:10.3390/pharmacy7030105 | No clinical outcomes were evaluated.  Wrong study design: retro-prospective study. |
| Attwood, 2023 | Attwood D, Vafidis J, Boorer J, Ellis W, Earley M, Denovan J, et al. 1365 PROACTIVE IT-assisted CGA in care homes improves adherence to preferred place of care and death, hospitalisation and mortality rates. Age Ageing. 2023;52(Suppl_1):afac322.084. doi:10.1093/ageing/afac322.084 | Conference abstract. |
| Baqir, 2012 | Baqir W et al. Reducing the ‘pill burden’ – complex multidisciplinary medication reviews. Int J Pharm Prac 2012; 20 (suppl 2) p31-101 | Conference abstract. |
| Beer, 2011 | Beer C, Loh PK, Peng YG, Potter K, Millar A. A pilot randomized controlled trial of deprescribing. Ther Adv Drug Saf. 2011;2(2):37-43. doi:10.1177/2042098611400332 | Wrong setting: community-dwelling participants were also included. |
| Bitter, 2017 | Bitter K, Jaehde U, Pehe C, Heuer G, Krüger M. Drug-related problems and symptom burden in nursing home residents. Int J Clin Pharm. 2017;39:220. | Conference abstract. |
| Bradburn, 2023 | Bradburn L, McNair S, Munang LA. 1580 Structured multidisciplinary reviews for care home residents reduce polypharmacy cost-effectively. Age Ageing. 2023;52(Suppl 2):afad104.021. Published July 21, 2023. doi:10.1093/ageing/afad104.021 | Conference abstract. |
| Cavalieri, 1993 | Cavalieri TA, Chopra A, Gray-Miceli D, Shreve S, Waxman H, Forman LJ. Geriatric assessment teams in nursing homes: do they work?. J Am Osteopath Assoc. 1993;93(12):1269-1272. | Intervention is a potential component of the geriatric assessment but is not explicitly defined. Additionally, targeted deprescribing is possible, as 75% of participants were admitted from an acute care facility, and the skilled nursing facility served as the primary teaching site for a University of Medicine and Dentistry. |
| Choi, 2024 | Choi JY, Kim H, Chun S, et al. Information technology-supported integrated health service for older adults in long-term care settings. BMC Med. 2024;22(1):212. Published 2024 May 29. doi:10.1186/s12916-024-03427-7 | Wrong setting: one university hospital and seven long‑term care hospitals were also included. |
| Crotty, 2004 | Crotty et al., An outreach geriatric medication advisory service in residential aged care: a randomized controlled trial of case conferencing, Age and Ageing 2004; 33: 612–617, doi:10.1093/ageing/afh213 | Targeted deprescribing: one of the two inclusion criteria was residents with difficult behavior about whom staff would like more advice and information. |
| Curtin, 2020 | Curtin D, Jennings E, Daunt R, et al. Deprescribing in Older People Approaching End of Life: A Randomized Controlled Trial Using STOPPFrail Criteria. J Am Geriatr Soc. 2020;68(4):762-769. doi:10.1111/jgs.16278 | Targeted deprescribing, i.e., patients with advanced frailty.  Wrong setting: hospitalized patients transferring to long-term nursing home care. |
| Davidsson, 2011 | Davidsson M, Vibe OE, Ruths S, Blix HS. A multidisciplinary approach to improve drug therapy in nursing homes. J Multidiscip Healthc. 2011;4:9-13. Published 2011 Jan 11. doi:10.2147/JMDH.S15773 | No clinical outcomes were evaluated. |
| Dellinger, 2020 | Dellinger JK, Pitzer S, Schaffler-Schaden D, et al. Improving medication appropriateness in nursing homes via structured interprofessional medication-review supported by health information technology: a non-randomized controlled study. BMC Geriatr. 2020;20(1):506. Published 2020 Nov 26. doi:10.1186/s12877-020-01895-z | No clinical outcomes were evaluated. |
| Doherty, 2020 | Doherty A, Miller R, Darcy C, Friel A, Mallett J, Shevlin M, Adamson G. Medicines optimisation in care homes via pharmacist case management: What is the impact on subsequent healthcare resource usage? Int J Pharm Pract. 2020;28(Suppl 1):73-74 | Conference abstract. |
| Dyer, 2004 | Dyer CA, Taylor GJ, Reed M, Dyer CA, Robertson DR, Harrington R. Falls prevention in residential care homes: a randomised controlled trial. Age Ageing. 2004;33(6):596-602. doi:10.1093/ageing/afh204 | Targeted deprescribing, i.e. focus on medications that increase the risk of falls. |
| Führling, 2022 | Führling C, Maas R. Extended Pharmacist Assessment of Medication Safety for Nursing Home Residents-A Cross-Sectional and Prospective Study. J Clin Med. 2022;11(21):6602. Published 2022 Nov 7. doi:10.3390/jcm11216602 | No clinical outcomes were evaluated. |
| Gorup, 2012 | Gorup EC. Effectiveness of a medication review in elderly nursing home residents. European General Practice Research Network Meeting, Ljubljana, Slovenia. 2012 | Conference abstract. |
| Gustafsson, 2015 | Gustafsson M, Sandman PO, Karlsson S, et al. Reduction in the use of potentially inappropriate drugs among old people living in geriatric care units between 2007 and 2013. Eur J Clin Pharmacol. 2015;71(4):507-515. doi:10.1007/s00228-015-1825-z | Wrong study design: retrospective cohort study. |
| Haider, 2022 | Haider I, Kosari S, Naunton M, et al. Quality Use of Medicines Indicators and Associated Factors in Residential Aged Care Facilities: Baseline Findings from the Pharmacists in RACF Study in Australia. J Clin Med. 2022;11(17):5189. Published 2022 Sep 1. doi:10.3390/jcm11175189 | Wrong study design: cross-sectional analysis of baseline characteristics of the participants in the Pharmacists in residential aged care facilities (PiRACF) study. |
| Haider, 2023 | Haider I, Kosari S, Naunton M, et al. Impact of on-site pharmacists in residential aged care facilities on the quality of medicines use: a cluster randomised controlled trial (PiRACF study). Sci Rep. 2023;13(1):15962. Published 2023 Sep 25. doi:10.1038/s41598-023-42894-5 | No clinical outcomes were evaluated. |
| Haider, 2023 | Haider I, Kosari S, Naunton M, et al. The role of on-site pharmacist in residential aged care facilities: findings from the PiRACF study. J Pharm Policy Pract. 2023;16(1):82. Published 2023 Jul 3. doi:10.1186/s40545-023-00587-4 | No clinical outcomes were evaluated. |
| Hashimoto, 2018 | Hashimoto R, Fujii K, Yoshida K, et al. [Outcomes of Pharmacists' Involvement with Residents of Special Nursing Homes for the Elderly]. Yakugaku Zasshi. 2018;138(9):1217-1225. doi:10.1248/yakushi.18-00065 | Article written in Japanese. |
| Hood, 1975 | Hood JC, Lemberger M, Steward RB. Promoting appropriate therapy in a long-term care facility. J Am Health Care Assoc. 1975;1(2):10-13. | No clinical outcomes were evaluated. |
| Howard, 2016 | Howard CJ, Bhattacharya S, Ray A, Howard H. Medication reduction in geriatric patients in a long-term care facility: A166. [Abstract]. J Am Geriatr Soc. 2016;64(Suppl 1):S75–S76. Presented at: Paper Abstracts: Poster Session A; May 19, 2016 | Conference abstract. |
| Hurley, 2022 | Hurley E, Foley T, Byrne S, Dalton K, Walsh E. Pharmacist-guided deprescribing for frail older adults in nursing homes using STOPPFRAIL: preliminary findings. Int J Clin Pharm. 2022;44(6):1469-1470 | Conference abstract. |
| Hurley, 2024 | Hurley E, Dalton K, Byrne S, Foley T, Walsh E. Pharmacist-Led Deprescribing Using STOPPFrail for Frail Older Adults in Nursing Homes. J Am Med Dir Assoc. 2024;25(9):105122. doi:10.1016/j.jamda.2024.105122 | Targeted deprescribing, i.e., terminally ill patients defined using the following criteria: patients with end-stage irreversible pathology, poor 1-year survival prognosis, severe functional and/or cognitive impairment, and symptom control is the priority as opposed to prevention of disease progression. |
| Juola, 2014 | Juola A-L, Bjorkman MP, Kautiainen H, Pylkkanen S, Finne-Soveri UH, Soini H, et al. Nursing staff education to reduce potentially harmful medication use among older people in assisted living facilities: effects of randomized controlled trial on cognition and falls. Eur Geriatr Med. 2014;5(Suppl 1):S178. doi:10.1016/S1878-7649(14)70100-7 | Conference abstract. |
| King, 2001 | King MA, Purdie DM, Roberts MS. Matching prescription claims with medication data for nursing home residents: implications for prescriber feedback, drug utilisation studies and selection of prescription claims database. J Clin Epidemiol. 2001;54(2):202-209. doi:10.1016/s0895-4356(00)00282-1 | Wrong study design: retrospective descriptive and comparative cohort study focused on the analysis of clinical and administrative data to evaluate the quality and reliability of medical records compared to pharmaceutical prescriptions. |
| King, 2023 | King S, Schwartz AW, Driver J, Ruopp M. The Age-Friendly Health System Initiative in Action: Caring for older veterans in skilled nursing facilities. J Am Geriatr Soc. 2023;71(Suppl 1):S117. Presented at: American Geriatrics Society Annual Scientific Meeting; May 3-6, 2023; California, USA. Abstract B78 | Conference abstract. |
| King, 2024 | King SE, Ruopp MD, Mac CT, et al. Early clinical and quality impacts of the Age-Friendly Health System in a Veterans Affairs skilled nursing facility. J Am Geriatr Soc. 2024;72(12):3865-3874. doi:10.1111/jgs.19083 | Wrong study design: ecological study, in particular quality improvement study. |
| Kosari, 2021 | Kosari S, Koerner J, Naunton M, et al. Integrating pharmacists into aged care facilities to improve the quality use of medicine (PiRACF Study): protocol for a cluster randomised controlled trial. Trials. 2021;22(1):390. Published 2021 Jun 11. doi:10.1186/s13063-021-05335-0 | Study protocol of Haider, 2022 (10.3390/jcm11175189), Haider, 2023 (10.1038/s41598-023-42894-5), and Haider, 2023 (10.1186/s40545-023-00587-4), that were not included. |
| Kretschmer, 2021 | Kretschmer E, Strasser D, Riedl R, Berghold A. PP008: GEMED: results of a project to improve pharmaceutical care for nursing home residents. In: 49th ESCP Virtual Symposium on Clinical Pharmacy: Clinical pharmacy, working collaboratively in mental health care. Int J Clin Pharm. 2021;43(6):1736-1801. [Published correction appears in Int J Clin Pharm. 2022;44(3):835-836.] doi:10.1007/s11096-021-01352-w | Conference abstract. |
| Lapane, 2007 | Lapane KL, Hughes CM, Quilliam BJ. Does incorporating medications in the surveyors' interpretive guidelines reduce the use of potentially inappropriate medications in nursing homes?. J Am Geriatr Soc. 2007;55(5):666-673. doi:10.1111/j.1532-5415.2007.01153.x | No clinical outcomes were evaluated. |
| Lee, 2017 | Lee JK, Alshehri S, Kurdi S, Amanti C, Mohler J. Interprofessional Team Care Program for Preventing Avoidable Admissions Among Assisted Living Elders (PA4LE). J Am Geriatr Soc 2017;65(suppl 1):S173-4 | Conference abstract. |
| Martin, 2017 | Martin C, Murphy Y. 86 The Integrated Community Ageing Team (ICAT) Pharmacy Service: Comprehensive medication reviews in the community. Age Ageing. 2017;46(Suppl 1):i1–i22. Published May 16, 2017. doi:10.1093/ageing/afx055.86 | Conference abstract. |
| Maruoka, 2023 | Maruoka H, Hamada S, Hattori Y, et al. Changes in chronic disease medications after admission to a Geriatric Health Services Facility: A multi-center prospective cohort study. Medicine (Baltimore). 2023;102(21):e33552. doi:10.1097/MD.0000000000033552 | Targeted deprescribing, i.e., only patients using medication for the treatment of hypertension, diabetes, and/or dyslipidemia. |
| Milos, 2013 | Milos V, Rekman E, Bondesson Å, et al. Improving the quality of pharmacotherapy in elderly primary care patients through medication reviews: a randomised controlled study. Drugs Aging. 2013;30(4):235-246. doi:10.1007/s40266-013-0057-0 | Wrong setting: community-dwelling patients were also included. |
| Milos Nymberg, 2021 | Milos Nymberg V, Lenander C, Borgström Bolmsjö B. The Impact of Medication Reviews Conducted in Primary Care on Hospital Admissions and Mortality: An Observational Follow-Up of a Randomized Controlled Trial. Drug Healthc Patient Saf. 2021;13:1-9. Published 2021 Jan 27. doi:10.2147/DHPS.S283708 | Wrong setting: community-dwelling patients were also included. |
| Nassaralla, 2014 | Nassaralla C, Nassaralla C, Khosla S, White H, Yanamadala M. Reducing medication use in a skilled nursing facility: A quality improvement project. J Am Med Dir Assoc. 2014;15(3):B22-B23. doi:10.1016/j.jamda.2013.12.060 | Conference abstract. |
| Nicholson, 2013 | Nicholson G, Nelson E, McNicholl S, McKee H, Cuthbertson J. Is an outreach service into nursing homes with a medication review leading to reduced medication burden and improved appropriateness of prescriptions? Age Ageing. 2013;42(Suppl_3):iii1–iii11. doi:10.1093/ageing/aft096 | Conference abstract. |
| Olearova, 2019 | Olearova A, Duban L. Identification of medication-related problems among the elderly nursing home residents. In: 47th ESCP Symposium on Clinical Pharmacy: Personalised pharmacy care. Int J Clin Pharm. 2019;41:289–383. Published 2018 Nov 29 | Conference abstract. |
| Olesen, 2023 | Olesen AE, Vaever TJ, Simonsen M, Simonsen PG, Høj K. Deprescribing in primary care without deterioration of health-related outcomes: A real-life, quality improvement project. Basic Clin Pharmacol Toxicol. 2024;134(1):72-82. doi:10.1111/bcpt.13925 | Wrong setting: 70 out of 105 (66.7%) included participants were community-dwelling patients. |
| Öztürk Bahat, 2015 | Öztürk Bahat G, Ozkaya H, Kılıç C, Muratli S, Ilhan B, Tufan A, et al. P-134: Efficacy and safety of training program concentrating on the Garfinkel method as a tool for reducing polypharmacy in nursing home residents. Eur Geriatr Med. 2015;6(Suppl 1):1-201 | Conference abstract. |
| Poudel, 2015 | Poudel A, Peel NM, Mitchell CA, Gray LC, Nissen LM, Hubbard RE. Geriatrician interventions on medication prescribing for frail older people in residential aged care facilities. Clin Interv Aging. 2015;10:1043-1051. Published 2015 Jun 25. doi:10.2147/CIA.S84402 | No clinical outcomes were evaluated. |
| Reidt, 2016 | Reidt SL, Holtan HS, Larson TA, et al. Interprofessional Collaboration to Improve Discharge from Skilled Nursing Facility to Home: Preliminary Data on Postdischarge Hospitalizations and Emergency Department Visits. J Am Geriatr Soc. 2016;64(9):1895-1899. doi:10.1111/jgs.14258 | Wrong setting: medication review was conducted in a skilled nursing facility at discharge with in-home or over-the-telephone follow-up. |
| Roberts, 1998 | Roberts MS, King M, Stokes JA, et al. Medication prescribing and administration in nursing homes. Age Ageing. 1998;27(3):385-392. doi:10.1093/ageing/27.3.385 | Wrong study design: cross-sectional study (survey) evaluating medications’ pattern of use. |
| Ruiz-Mendoza, 2020 | Ruiz-Mendoza E, Penart A, Obi I, Addison E, Clark H. 37 Enhance GP–Geriatrician Care Homes Multidisciplinary Team. Age Ageing. 2020;49(Suppl 1):i9–i10. Published February 6, 2020. doi:10.1093/ageing/afz184.04 | Conference abstract. |
| Samala, 2011 | Samala R, Loquias JE, Galindo D, Ciocon J. Reducing nursing home polypharmacy using systematic medication and assessment review and tracking: the SMART Program. J Am Geriatr Soc. 2011;59(9):1771-1773. doi:10.1111/j.1532-5415.2011.03547.x | Letter to the Editor. |
| Schaffler-Schaden, 2018 | Schaffler-Schaden D, Pitzer S, Schreier M, et al. Improving medication appropriateness in nursing home residents by enhancing interprofessional cooperation: A study protocol. J Interprof Care. 2018;32(4):517-520. doi:10.1080/13561820.2018.1448372 | Study protocol of Dellinger, 2020 (DOI: 10.1186/s12877-020-01895-z) and Schreier, 2022 (DOI: 10.1186/s12913-022-08562-6), both studies were not included. |
| Schreier, 2022 | Schreier MM, Pitzer S, Dellinger JK, Schaffler-Schaden D, Osterbrink J, Flamm M. Evaluation of an intervention to improve the safety of medication therapy via HIT-supported interprofessional cooperation in long-term care - a mixed method study. BMC Health Serv Res. 2022;22(1):1227. Published 2022 Oct 3. doi:10.1186/s12913-022-08562-6 | No clinical outcomes were evaluated. |
| Schmidt, 2012 | Schmidt IK, Fastbom J. Quality of drug use in Swedish nursing homes. Clin Drug Investig. 2000;20(6):433-446. doi:10.2165/00044011-200020060-00006 | No medication review/deprescribing intervention was performed. This study described, analyzed, and discussed the use of drugs among residents in nursing homes. |
| Stuhec, 2019 | Stuhec M, Bratović N, Mrhar A. Impact of clinical pharmacist's interventions on pharmacotherapy management in elderly patients on polypharmacy with mental health problems including quality of life: A prospective non-randomized study. Sci Rep. 2019;9(1):16856. Published 2019 Nov 14. doi:10.1038/s41598-019-53057-w | Targeted deprescribing, i.e., focus on patients with mental health problems, in therapy with at least one psychotropic drug. |
| Stuijt, 2012 | Stuijt CC, Franssen EJ, Egberts AC, Hudson SA. Appropriateness of prescribing among elderly patients in a Dutch residential home: observational study of outcomes after a pharmacist-led medication review. Drugs Aging. 2008;25(11):947-954. doi:10.2165/0002512-200825110-00005 | No clinical outcomes were evaluated. |
| Tommelein, 2018 | Tommelein E. Deprescribing in nursing homes is safe and should be pursued. Evid Based Nurs. 2018;21(2):53. doi:10.1136/eb-2018-102885 | Letter to the Editor. |
| Trygstad, 2009 | Trygstad TK, Christensen DB, Wegner SE, Sullivan R, Garmise JM. Analysis of the North Carolina long-term care polypharmacy initiative: a multiple-cohort approach using propensity-score matching for both evaluation and targeting. Clin Ther. 2009;31(9):2018-2037. doi:10.1016/j.clinthera.2009.09.006 | Targeted deprescribing, i.e., residents that had a hospitalization or an ED visit in the preintervention period were excluded, as well as residents that did not have a prescription filled in the first 35 days of the preintervention period or the last 35 days of the post-period. Additionally, medication review was conducted retrospectively based on claims data from Medicaid. |
| Wouters, 2014 | Wouters H, Quik EH, Boersma F, et al. Discontinuing inappropriate medication in nursing home residents (DIM-NHR Study): protocol of a cluster randomised controlled trial. BMJ Open. 2014;4(10):e006082. Published 2014 Oct 8. doi:10.1136/bmjopen-2014-006082 | Study protocol of Wouter, 2017 (DOI: 10.7326/M16-2729, that was not included. |
| Wouters, 2017 | Wouters H, Scheper J, Koning H, et al. Discontinuing Inappropriate Medication Use in Nursing Home Residents: A Cluster Randomized Controlled Trial. Ann Intern Med. 2017;167(9):609-617. doi:10.7326/M16-2729 | Targeted deprescribing for residents in dementia special care units or units providing care for individuals with disabling conditions in nursing homes. |
| Yeong, 2013 | Yeong K, Ralphson A, Lisk R. Prevention of falls in care homes – a model of integrated care. Presented at: 9th Congress of the EUGMS; October 2–4, 2013; Venice, Italy. Eur Geriatr Med. 2013;4(S1):S60–S61. doi:10.1016/j.eurger.2013.07.199 | Conference abstract. |

**Table A4**. Characteristics of the studies included in the systematic review.

| **Author, Year**  **Country** | **Participants, N.** | **Age in years, mean (SD) *** | **Enrolment criteria** | **Healthcare professionals involved** | **Tools (including educational programs) used for the intervention** | **Intervention timing and follow-up duration** | **Outcome measures** | **Study results** |
| --- | --- | --- | --- | --- | --- | --- | --- | --- |
| **Randomized controlled trials** | | | | | | | | |
| Schmidt, 1998  Sweden | Total: 1,854  IG: 626  CG: 1,228 | Overall: 83  IG:  ≤65 y: 5%  66-84 y: 45%  ≥85 y: 50%  CG:  ≤65 y: 3%  66-84 y: 47%  ≥85 y: 50% | No specific inclusion/exclusion criteria were reported | GP, a pharmacist, nurses, undernurses (similar to licensed practical nurses), and nurse’s aides | - The pharmacists were specially trained prior to and during the intervention period in gerontology/geriatrics, drug use in the elderly, and basic skills for working in small groups - SMPA guidelines for drug treatment in the elderly and the demented | Total study period: 19 months. The intervention was performed monthly for 1 year, and assessments were carried out 1 month after therapy change | Prescribed drugs, from baseline to end of the intervention period, mean | IG: 7.5 to 7.8  CG: 7.8 to 8.2 |
|  |  |  |  |  |  |  | Quality of life consequences (e.g., daytime sedation, sleep) of the 532 drug changes, proportion | Beneficial effect: 101 (19.0%)  No observable effect: 250 (47.0%)  Negative effect: 43 (8.1%)  Too difficult to evaluate: 138 (25.9%) |
| Furniss, 2000  United Kingdom | Total: 330  IG: 158  CG: 172 | IG: 83.5 (9.2)  CG: 78.9 (13.7) | Inclusion criteria (for the LTCFs involved):   - Each home in the matched pair, to be subsequently randomized, was selected from distinct geographical areas to avoid the potential conflict of a GP overseeing residents in both a control home and an intervention home | GPs, pharmacist, psychiatrist, home staff | - US OBRA guidelines (with a focus, but not limited to, on antipsychotics) | A 4-month observation phase was followed by a 4-month intervention | Number of drug from the beginning of the intervention phase to month 4 of follow-up, mean per patient | IG: 5.1 to 4.2  CG: 4.5 to 4.4  Mean adjusted difference (95% CI): 0.5 (-0.04, 1.0)  *p*= 0.07 |
|  |  |  |  |  |  |  | Cognitive function (MMSE), from the beginning of the intervention phase to month 4 of follow-up, mean per patient | IG: 13.5 to 12.5  CG: 15.5 to 17.1  Mean adjusted difference (95% CI): 1.6 (-0.1, 3.3)  *p*= 0.07 |
|  |  |  |  |  |  |  | Depression (GDS), from the beginning of the intervention phase to month 4 of follow-up, mean per patient | IG: 4.74 to 4.41  CG: 4.35 to 3.86  Mean adjusted difference (95% CI): -0.75 (-2.03, 0.52)  *p*= 0.25 |
|  |  |  |  |  |  |  | Depression (BASDEC), from the beginning of the intervention phase to month 4 of follow-up, mean per patient | IG: 4.72 to 3.77  CG: 3.83 to 3.26  Mean adjusted difference (95% CI): -0.18 (-1.45, 1.09)  *p*= 0.79 |
|  |  |  |  |  |  |  | Behavior (CRBRS), from the beginning of the intervention phase to month 4 of follow-up, mean per patient | IG: 18.8 to 19.4  CG: 15.1 to 14.5  Mean adjusted difference (95% CI): -2.2 (-4.1, -0.3)  *p*= 0.02 |
|  |  |  |  |  |  |  | Falls | No significant differences between IG and CG |
|  |  |  |  |  |  |  | Hospitalization days, observation phase vs. intervention phase, mean per patient | IG: 1.44 to 0.55  CG: 1.51 to 1.26 |
|  |  |  |  |  |  |  | Deaths from the beginning of the intervention phase to month 4 of follow-up, counts (percentage) | IG: 4 (2.9) vs. CG: 14 (8.9) |
| Roberts, 2001  Australia | Total: 3,230 IG: 905  CG: 2,325 | 98% and 97.4% over 60 years for IG and CG, respectively | Inclusion criteria (for the LTCFs involved):   - At least 20 residents - Within 3 h drive from the study center in Brisbane - Supply of drugs under the Australian government medication subsidy scheme (Pharmaceutical Benefits Scheme) - Central record of hospitalizations, adverse events, and deaths | Clinical pharmacists, nurses, GPs, geriatrician, research nurse​ | - Problem-based education sessions were provided to nurses. Sessions addressed basic geriatric pharmacology and some common problems in long-term care (depression, delirium, dementia, incontinence, falls, sleep disorders, constipation, and pain). Sessions were supported by wall charts, bulletins, telephone calls, and clinical pharmacy visits - No validated tool was used or described (e.g., software, criteria, etc.) | Intervention duration: 12 months.  Survival follow-up: 22 months after intervention started | Medication appropriateness (decrease in cumulative drug use at 12 months), % change IG vs. CG. | 14.8%  *p*= 0.073  *p*< 0.0005 (without considering the clustering effect of nursing homes) |
|  |  |  |  |  |  |  | Disability evaluation (RCI) from baseline to month 12 of follow-up, % change (95% CI) | IG: +5.52 (1.45, 9.59)  CG: +5.73 (4.44, 7.02)  *p*= 0.253 |
|  |  |  |  |  |  |  | Adverse events (3 months prior vs. last 3 months of follow-up), % mean change (95% CI) | IG: +54.02 (-44.51, 152.56)  CG: +49.07 (-21.17, 119.31)  *p*= 0.388 |
|  |  |  |  |  |  |  | Hospitalizations (12 months prior vs. 12-month follow-up), post-study mean percentage (95% CI) | IG: 15.86 (10.55-21.16) %  CG: 18.36 (15.07-21.65) % |
|  |  |  |  |  |  |  | Deaths at 12 months, proportion | IG: 216 (23.9%) vs. CG: 617 (26.5%) |
|  |  |  |  |  |  |  | Deaths at 22 months, proportion | IG: 323 (35.7%) vs. CG: 998 (42.9%)  Hazard ratio 0.85 (95% CI 0.68, 1.06), *p*= 0.13  Hazard ratio: 0.85 (95% CI 0.75, 0.96), *p*< 0.009 (without considering the clustering effect of nursing homes) |
| Zermansky, 2006  United Kingdom | Total: 661  IG: 331  CG: 330 | IG: 85.3 (IQR: 81-90)  CG: 84.9 (IQR: 80-90) | Inclusion criteria (for the LTCFs involved):   - 6 or more residents aged 65 years or older   Inclusion criteria:   - 65 years or older - One or more chronic medication   Exclusion criteria:   - Enrolled in another clinical trial - Terminally ill (life expectancy < 1 month) - Patients already receiving medication review by a pharmacist - Exclusion by GP’s request | Clinical pharmacist, GP, trained nurse​ | - Process algorithm based on that described by Lowe et al. (2000), but adapted to the care home setting - Local and national guidelines - Guidelines produced by the North West Drug Information Service | The intervention was performed within 28 days of randomization. Patients were followed for 6 months (±3 weeks) from randomization. | Number of drug changes at 6 months, mean per patient (SD) | IG: 3.1 (2.7) vs. CG: 2.4 (2.6)  Difference (relative risk 95% CI): 1.34 (1.21-1.48)  *p*< 0.0001 |
|  |  |  |  |  |  |  | Physical function (Barthel Index), mean change per patient at month 6 | IG: -0.3 vs. CG: -0.8  Mean difference (95% CI): 0.46 (-0.02, 0.94)  *p*= 0.06 |
|  |  |  |  |  |  |  | Cognitive function (standardized MMSE), mean change per patient at month 6 | IG: +0.1 vs. CG: +0.7  Mean difference (95% CI): -0.24 (-1.18, 0.70)  *p*= 0.62 |
|  |  |  |  |  |  |  | Falls (6 months prior vs. 6-month follow-up), mean per patient (SD) | IG: 1.0 (1.7) to 0.8 (1.7)  CG: 0.9 (1.7) to 1.3 (3.1)  Relative risk, adjusted (95% CI): 0.59 (0.49, 0.70), *p*< 0.0001 |
|  |  |  |  |  |  |  | Hospitalizations (6 months prior vs. 6-month follow-up), mean per patient (SD) | IG: 0.23 (0.52) to 0.20 (0.48)  CG: 0.23 (0.57) to 0.26 (0.61)  Relative risk, adjusted (95% CI): 0.75 (0.52, 1.07), *p*= 0.11 |
|  |  |  |  |  |  |  | Deaths at 6 months, proportion | IG: 51 (15.3%) vs. CG: 48 (14.5%)  Difference OR (95% CI): 0.89 (0.56,1.41)  *p*= 0.81 |
| Pope, 2010  United Kingdom | Total: 225  IG: 110  CG: 115 | IG: 83.3 (NR)  CG: 82.5 (NR) | All permanent patients on the nurse-managed continuing-care wards were included. No specific inclusion/exclusion criteria were reported | Consultant geriatricians, specialist registrars in geriatric medicine, registered hospital pharmacists, senior nurse practitioners, GPs | - American Geriatrics Society Beers criteria® 2003 version - IPET - The British National Formulary | Follow-up data were available on all study patients at 6 months. | Regular medications, mean/median from baseline to month 6 | IG: 6.11/6 to 5.55/5  CG: 5.91/6 to 5.83/6 |
|  |  |  |  |  |  |  | Cognitive function (AMTS), mean/median from baseline to month 6 | IG: 4.2/4 to 4.1/3  CG: 4.2/3 to 4.2/3 |
|  |  |  |  |  |  |  | Quality of Life (Barthel Index), mean/median from baseline to month 6 | IG: 5.95/4 to 5.94/4  CG: 6.75/4 to 6.62/3 |
|  |  |  |  |  |  |  | Admissions to acute hospital at month 6 of follow-up, N. (%) | IG: 11 (10.0) vs. CG: 6 (5.2)  *p*= 0.213 |
|  |  |  |  |  |  |  | Deaths at month 6 of follow-up, N. (%) | IG: 17 (15.5) vs. CG: 11 (9.6)  *p*= 0.226 |
| Frankenthal, 2014  Israel | Total: 359  IG: 183  CG: 176 | Overall: 82.7 (8.7)  IG:  65-74 y: 15.8%  75-84 y: 34.4%  ≥85 y: 49.7%  CG:  ≤65 y: 20.5%  66-84 y: 35.8%  ≥85 y: 43.8% | Inclusion criteria:   - 65 years or older - At least 1 daily drug   Exclusion criteria:   - Terminally ill residents - Patients whose stay in the facility was shorter than 3 months | Study pharmacist, facility physicians, nurses | - STOPP/START criteria 2008 version | The intervention was performed at the study opening and 6 and 12 months later. Assessments were conducted at 12 and 24 months. | PIPs from baseline to month 12 of follow-up, proportion | IG: 70.5% to 22.5% vs. CG: 64.7% to 54.1%  *p*< 0.001 |
|  |  |  |  |  |  |  | PIPs at 24 months, proportion | IG: 33.3% vs. CG: 48.4%  *p*= 0.02 |
|  |  |  |  |  |  |  | PPOs from baseline to month 12 of follow-up, proportion | IG: 35.5% to 6.3% vs. CG: 32.4% to 21.9%  *p*< 0.001 |
|  |  |  |  |  |  |  | PPOs at 24 months, proportion | IG: 26.2% vs. CG: 34.1%  *p*= 0.21 |
|  |  |  |  |  |  |  | Number of medications from baseline to month 12 of follow-up, mean (SD) | IG: 8.8 (3.4) to 7.3 (2.7)  CG: 8.2 (3.0) to 8.9 (3.2)  *p*< 0.001 |
|  |  |  |  |  |  |  | Functioning (FIM) from baseline to month 12 of follow-up, mean per patient | IG: 58.4 to 54.3 vs. CG: 58.9 to 55.4  *p*= 0.14 |
|  |  |  |  |  |  |  | Falls from baseline to month 12 of follow-up, mean per patient (SD) | IG: 1.3 (2.4) to 0.8 (1.3)  CG: 1.4 (2.5) to 1.3 (2.4)  *p*= 0.28 |
|  |  |  |  |  |  |  | Falls at 24 months, mean per patient (SD) | IG: 0.9 (1.4) vs. CG: 0.7 (1.4)  *p*= 0.40 |
|  |  |  |  |  |  |  | Hospitalizations from baseline to month 12 of follow-up, mean per patient (SD) | IG: 0.6 (1.0) to 0.5 (1.0)  CG: 0.4 (0.8) to 0.5 (0.9)  *p*= 0.10 |
|  |  |  |  |  |  |  | Hospitalizations at 24 months, mean per patient (SD) | IG: 0.6 (1.1) vs. CG: 0.4 (0.6)  *p*= 0.50 |
|  |  |  |  |  |  |  | Quality of life (SF-12) at 12 months | Physical component: no differences between groups (*p*= 0.09)  Mental component: no differences between groups (*p*= 0.70) |
|  |  |  |  |  |  |  | Deaths at 12 months, proportion | IG: 15 (8.2%) vs. 17 (9.7%)  *p*= 0.90 |
|  |  |  |  |  |  |  | Deaths at 24 months, proportion | IG: 34 (18.6%) vs. 30 (17.0%)  *p*= 0.90 |
| García-Gollarte, 2014  Spain | Total: 1,018  IG: 516  CG: 502 | Overall: 84.4 (12.7)  IG: 84.24 (14.6)  CG: 84.5 (10.4) | Inclusion criteria:   - 65 years or older - Patients whose stay in the facility was at least 3 months and expected to stay in it for the length of the study - Clinically stable (no changes in prescription in the last 2 months)   Exclusion criteria:   - Palliative care - Cared from primary care providers outside the nursing home | Nursing home physicians, registered nurses, physiotherapists, psychologists, occupational therapist​ | - Educational intervention delivered to physicians - Educator (i.e., a nursing home physician expert in drug use in older people) on-demand advice - STOPP/START criteria 2008 version | Intervention duration: 6 months, evaluations were conducted in the 3-month period immediately after | STOPP criteria per patient from baseline to end of the follow-up, mean (SD) | IG: 1.22 (1.24) to 0.81 (1.13), *p=* 0.000  CG: 1.10 (1.15) to 1.29 (1.56), *p*= 0.000 |
|  |  |  |  |  |  |  | START criteria per patient from baseline to end of the follow-up, mean (SD) | IG: 0.91 (1.19) to 0.13 (0.44), *p=* 0.000  CG: 0.76 (0.92) to 0.85 (1.08), *p*= 0.101 |
|  |  |  |  |  |  |  | Number of medications from baseline to month 6 of follow-up, mean (SD) | IG: 8.25 (3.39) to 3.64 (4.05), *p*= 0.000  CG: 7.89 (3.27) to 4.48 (4.97), *p*= 0.001 |
|  |  |  |  |  |  |  | Number of falls per patient (3 months prior vs. 3-month follow-up), mean (SD) | IG: 0.40 (0.90) to 0.32 (0.68), *p*= 0.251  CG: 0.34 (1.04) to 0.43 (0.86), *p*= 0.003 |
|  |  |  |  |  |  |  | Falls, proportion of patients with at least 1 fall | IG: 82/344 (23.8%) vs. CG: 104/372 (27.8%)  *p*= 0.251 |
|  |  |  |  |  |  |  | Number of delirium per patient (3 months prior vs. 3-month follow-up), mean (SD) | IG: 0.08 (0.32) to 0.03 (0.20), *p*= 0.035  CG: 0.04 (0.24) to 0.14 (0.58), *p*= 0.001 |
|  |  |  |  |  |  |  | Number of ED visits per patient (3 months prior vs. 3-month follow-up), mean (SD) | IG: 0.12 (0.38) to 0.15 (0.43), *p*= 0.179  CG: 0.12 (0.43) to 0.24 (0.93), *p*= 0.022 |
|  |  |  |  |  |  |  | Number of days spent in hospital per patient (3 months prior vs. 3-month follow-up), mean (SD) | IG: 0.44 (1.81) to 0.45 (1.83), *p*= 0.822  CG: 0.3 (1.81) to 0.68 (2.76), *p*= 0.011 |
|  |  |  |  |  |  |  | Hospitalizations (3 months prior vs. 3-month follow-up), proportion | IG: 31 (9.01%) to 30 (8.72%), *p*= 0.822  CG: 20 (5.38%) to 35 (9.41%), *p*= 0.011 |
|  |  |  |  |  |  |  | Deaths at the end of the follow-up, proportion | IG: 56 (10.9%) vs. CG: 45 (9.0%)  *p*= 0.561 |
| Pitkälä, 2014  Finland | Total: 227  IG: 118  CG: 109 | Overall: 83 (NR)  IG: 82.9 (7.5)  CG: 83.5 (6.9) | Inclusion criteria:   - 65 years or older - Living permanently in the facility - Finnish speaking - Using at least 1 medication - Having an estimated life expectancy of >6 months | Nursing staff, physicians (consultants), study nurses, research nurses | - Educational sessions delivered to nurses - American Geriatrics Society Beers criteria® 2003 version - Anticholinergic Risk Scale - Swedish list of medications with anticholinergic properties | Intervention duration: 12 months. Assessments were performed at 6 and 12 months | Harmful drug use, mean change (95% CI) per patient from baseline to month 12 of follow-up | IG: -0.43 (-0.15, -0.71), *p*= 0.0024  CG: +0.11 (-0.09, 0.31), *p*= 0.27  *p* adjusted= 0.0035 |
|  |  |  |  |  |  |  | Quality of life (15D instrument of health-related quality of life), mean change (95% CI) per patient at 12 months | IG: -0.038 (-0.054, -0.022)  CG: -0.072 (-0.089, -0.055)  *p* adjusted= 0.005 |
|  |  |  |  |  |  |  | Falls/person year (95% CI) | IG: 2.25 (1.93, 2.62)  CG: 3.25 (2.87, 3.67)  Incidence rate ratio, adjusted (95% CI): 0.72 (0.59, 0.88), *p*< 0.001 |
|  |  |  |  |  |  |  | Hospitalization days, mean (95% CI) per patient from baseline to month 12 of follow-up | IG: 1.4 (1.2, 1.6)  CG: 2.3 (2.1, 2.7)  Incidence rate ratio, adjusted (95% CI): 0.60 (0.49, 0.75), *p*< 0.001 |
|  |  |  |  |  |  |  | Deaths at 6 months, proportion | IG: 25 (21.2%) vs. CG: 13 (11.9%) |
|  |  |  |  |  |  |  | Deaths at 12 months, proportion | IG: 39 (33.1%) vs. CG: 24 (22.0%)  Hazard ratio, adjusted (95% CI): 1.04 (0.79, 1.36), *p*= 0.79 |
| Potter, 2016  Australia | Total: 95  IG: 47  CG: 48 | Overall: 84.3 (6.9)  IG: 84 (6)  CG: 84 (8) | Inclusion criteria:   - 65 years or older   Exclusion criteria:   - Taking no regular medicine - Terminal stages of an illness - Exclusion by GP’s or nurse manager request | GP, geriatrician, clinical pharmacologist, pharmacists, registered nurse​ | - List of PIMs - Deprescribing algorithm | Assessments were performed at 12 months post-randomization | Number of unique regular medicines, mean change per patient at 12 months (SD) | IG: -1.9 (4.1) vs. CG: 0.1 (3.5)  Estimated difference (95% CI): 2.0 (0.08, 3.8)  *p*= 0.04 |
|  |  |  |  |  |  |  | Quality of life (EQ-5D), mean change per patient at 12 months (SD) | IG: -11 (17) vs. CG: 7 (15)  *p* adjusted= 0.35 |
|  |  |  |  |  |  |  | Quality of life (QoL-AD), mean change per patient at 12 months (SD) | IG: -1.0 (4.3) vs. CG: -1.0 (4.7)  *p* adjusted= 0.91 |
|  |  |  |  |  |  |  | Cognitive function (MMSE), mean change per patient at 12 months (SD) | IG: -3 (5) vs. CG: -2 (4)  *p* adjusted= 0.60 |
|  |  |  |  |  |  |  | Physical function (Modified Barthel Index), mean change per patient at 12 months (SD) | IG: -10 (17) vs. CG: -11 (15)  *p* adjusted= 0.76 |
|  |  |  |  |  |  |  | Falls from baseline to month 12 of follow-up, proportion (95% CI) | IG: 0.56 (0.42, 0.69) vs. CG: 0.65 (0.50, 0.77)  *p*= 0.40 |
|  |  |  |  |  |  |  | Hospitalizations from baseline to month 12 of follow-up, proportion (95% CI) | IG: 0.51 (0.37, 0.61) vs. CG: 0.50 (0.36, 0.63)  *p*= 0.99 |
|  |  |  |  |  |  |  | Deaths at 12 months, proportion | IG: 12 (26%) vs. CG: 19 (40%)  Hazard ratio (95% CI): 0.60 (0.30, 1.22)  *p*= 0.16 |
| Husebo, 2019  Norway | Total: 723  IG: 394  CG: 329 | Overall: 86.7 (7.5)  IG: 86.5 (7.7)  CG: 87.0 (7.2) | Inclusion criteria:  -NH patients with and without dementia  -65 years or older  Exclusion criteria:  -Life expectancy less than 6 months  -Patients with diagnoses of schizophrenia | Nursing staff, physicians, nursing home managers, registered nurses, researchers | -Education seminar  -STOPP/START criteria 2015 version  -Anticholinergic list  -www.interaksjoner.no | The intervention lasted for 4 months. Data was collected at baseline, month 4, and month 9. | Difference in regular drugs from baseline to month 9, number (95% CI) | IG: -1.1 (-1.5, -0.7) vs. CG: -0.4 (0.9, 0.0)  Intervention effect: -0.6 (-1.2, -0.1), *p*<0.05 |
|  |  |  |  |  |  |  | Physical function (ADL), mean difference from baseline to month 9, number (95% CI) | IG: 0.1 ( 0.8, 1.0) vs. CG: 1.6 (0.6, 2.5)  Intervention effect: 1.4 ( 2.7, 0.1), *p*<0.05 |
|  |  |  |  |  |  |  | Quality of life (QUALID), mean difference from baseline to month 9, number (95% CI) | IG: 0.5 (-0.7, 1.7) vs. CG: -0.1 (-1.4, 1.2)  Intervention effect: 0.6 (-1.1, 2.4) |
|  |  |  |  |  |  |  | Quality of life (EQ-VAS), mean difference from baseline to month 9, number (95% CI) | IG: -1.6 (-5.5, 2.3) vs. CG: -4.9 (-9.1, -0.7)  Intervention effect: 3.3 (-2.4, 9.0) |
|  |  |  |  |  |  |  | Quality of life (QUALIDEM total score), mean difference from baseline to month 9, number (95% CI) | IG: -1.3 (-2.6, 0.0) vs. CG: -1.0 (-2.5, 0.4)  Intervention effect: -0.3 (-2.2, 1.6) |
|  |  |  |  |  |  |  | Deaths at 4 months, proportion | IG: 28 (7.1%) vs. CG: 33 (10.0%) |
|  |  |  |  |  |  |  | Deaths at 9 months, proportion | IG: 66 (16.7%) vs. CG: 47 (14.3%) |
| Strauven, 2019  Belgium | Total: 1,804 IG: 847  CG: 957 | IG, median (IQR): 87 (82-92)  CG, median (IQR): 88 (83-92) | Inclusion criteria (for the LTCFs involved):   - 35 residents or more   Inclusion criteria:   - 65 years or older - Under the care of a participating GP   Exclusion criteria:   - Patients receiving palliative care - Patients in subacute care/rehabilitation | GPs, pharmacists, nurses | - Educational sessions: e-learning and (interdisciplinary) face-to-face workshops - No validated tool was used or described (e.g., software, criteria, etc.) | Study duration: 15 months. The intervention was conducted every 4 months. Follow-up data was collected at months 1,8, and 15 | At least one PIM or PPO that was present at baseline had been solved at the end of the study + when there was no new PIM or PPO at the end of the study compared with baseline | Odds ratio (95% CI): 1.479 (1.062, 2.059)  *p*= 0.21 |
|  |  |  |  |  |  |  | ED visits, proportion of patients with at least one visit | IG: 51 (10.7%) vs. CG: 71 (13.9%)  Odds ratio (95% CI): 0.742 (0.397, 1.386)  *p*= 0.3482 |
|  |  |  |  |  |  |  | Hospitalizations from baseline to month 15 of follow-up, proportion (95% CI) | IG: 70 (14.7%) vs. CG: 88 (17.3%)  Odds ratio (95% CI): 0.934 (0.561, 1.556)  *p*= 0.7934 |
|  |  |  |  |  |  |  | Hospitalizations, median days per hospitalization (IQR) | IG: 7.0 (0-11) vs. CG: 8.0 (3-15.8)  Ratio of geometric means (95% CI): 0.578 (0.366, 0.913), *p*= 0.0203 |
|  |  |  |  |  |  |  | Deaths at 15 months, proportion | IG: 226 (26.7%) vs. CG: 207 (21.6%)  *p*= 0.0153 |
| Balsom, 2020  Canada | Total: 45  IG: 22  CG: 23 | IG: 84.3 (range: 76-97)  CG: 84.5 (range: 67-99) | Inclusion criteria:   - 65 years or older   Exclusion criteria:   - Not taking regular scheduled medications - Patients receiving palliative care | Pharmacists, pharmacy students, attending physicians, nursing staff | - Educational session about deprescribing and polypharmacy provided by pharmacy students to nursing and support staff - Deprescribing algorithm | Follow-up data was collected at months 3 and 6. Participants were reviewed weekly | Medication, mean change (95% CI) from baseline to month 6 | IG -2.88 (-4.284, -1.071) than the CG |
|  |  |  |  |  |  |  | Quality of life (RAI), changes from baseline to month 6 | No differences between IG and CG |
|  |  |  |  |  |  |  | Deaths at 6 months, proportion | IG: 4 (18.2%) vs. CG: 3 (13.0%) |
| Desborough, 2020  United Kingdom | Total: 826  IG: 381  CG: 445 | IG: 88.4 (6.5)  CG: 86.0 (8.5) | Inclusion criteria (for the LTCFs involved):   - 65 years or older - Consent sought from the GPs   Exclusion criteria (for the LTCFs involved):   - Already received a medication review service from the primary care organization in the last 6 months - Receiving ongoing medication services from a community geriatrician - Subject to investigation of the safeguarding of vulnerable adults   Exclusion criteria (for the patients):   - Self-medicating - Respite care | GPs, clinical pharmacists, care home staff, pharmacy technicians​ | - No validated tool was used or described (e.g., software, criteria, etc.) | Follow-up was determined as being 12 months from the date of ﬁrst medication review in intervention homes and 12 months from an equivalent period (to intervention homes) after allocation in control homes. Interventions were performed at the beginning and after 6 months | PIM reduction | Rate ratio months 1-6, adjusted (95% CI): 0.85 (0.71, 1.01), *p*= 0.060  Rate ratio months 6-12, adjusted (95% CI): 0.82 (0.67, 1.00), *p*= 0.046 |
|  |  |  |  |  |  |  | Falls from baseline to month 12 of follow-up, mean per patient (SD) | IG: 3.35 (8.30) vs. CG: 3.00 (5.49)  Rate ratio, adjusted (95% CI): 1.02 (0.74, 1.39)  *p*= 0.910 |
|  |  |  |  |  |  |  | ED visits from baseline to month 12 of follow-up, mean per patient (SD) | IG: 0.88 (2.01) vs. CG: 0.72 (2.09)  Rate ratio, adjusted (95% CI): 1.19 (0.86, 1.64)  *p*= 0.286 |
|  |  |  |  |  |  |  | Deaths at 6 months, proportion | IG: 94 (24.7%) vs. CG: 98 (22.0%) |
|  |  |  |  |  |  |  | Deaths at 12 months, proportion | IG: 125 (32.8%) vs. CG: 153 (34.4%)  Hazard ratio, adjusted (95% CI): 0.98 (0.72, 1.31), *p*= 0.868 |
| Kua, 2020  Singapore | Total: 295  IG: 153  CG: 142 | IG: 80.57 (9.42)  CG: 80.02 (9.58) | Inclusion criteria:  -65 years or older  -Taking 5 or more medications  Exclusion criteria:  -Life expectancy less than 6 months  -Respite care | Pharmacists, physicians, nurses | - Deprescribing guide consisting of the American Geriatrics Society Beers criteria® 2015 version, STOPP criteria 2015 version, as well as drug interaction checking | Medication review services were provided weekly or fortnightly. Data was collected at baseline, month 3, month 6, and month 12 | Pill burden, mean number of regular medications at end of study roll-out (SD) | IG: 9.48 (4.78) vs. CG: 9.68 (4.48)  Difference (95% CI): -0.20 (-1.69, 0.43)  *p*= 0.24 |
|  |  |  |  |  |  |  | Fall rates (n. of fallers within the past 3 months), n/total (%) | IG: 23/415 (55.4) vs. CG: 18/437 (41.1)  Odds ratio (95% CI) 1.37 (0.73, 2.57)  *p*= 0.33 |
|  |  |  |  |  |  |  | Hospitalizations, n/total (%) | IG: 56/415 (15.4) vs. CG: 97/437 (22.7)  Hazard ratio (95% CI): 0.16 (0.10, 0.26)  *p*< 0.001 |
|  |  |  |  |  |  |  | Deaths, n/total (%) | IG: 10/415 (2.4) vs. CG: 23/437 (5.3)  Hazard ratio (95% CI): 0.16 (0.07, 0.41)  *p*< 0.001 |
| Sluggett, 2020  Australia | Total: 242  IG: 99  CG: 143 | IG: 85.7 (7.8)  CG: 86.2 (8.3) | Inclusion criteria:   - 1 or more regular drugs - Permanent residents   Exclusion criteria:   - Estimated to have less than 3 months to live - Deemed to be medically unstable | Clinical pharmacists, GPs, registered nurses, enrolled nurses, residential services managers | - 5-item Medication Regimen Simpliﬁcation Guide for Residential Aged CarE (MRS GRACE) | Intervention: within 2 weeks of baseline data collection. Follow-up data collection: at months 4 and 12 | Number of charted medication administration times over a 24-hour period from baseline to month 12 of the follow-up, mean per patient (SD) | IG: 3.9 (1.5) to 3.6 (1.4)  CG: 4.0 (1.8) to 4.0 (1.7)  Mean difference (95% CI): -0.36 (-0.63, -0.09)  *p*= 0.010 |
|  |  |  |  |  |  |  | Medication incidents from baseline to month 4 of follow-up | Incident rate ratio, adjusted (95% CI): 1.42 (0.75, 2.66), *p*= 0.27 |
|  |  |  |  |  |  |  | Physical function (ADL) from baseline to month 12 of follow-up, median (IQR) | IG: 1 (1-3) to 2 (1-4)  CG: 1 (1-3) to 2 (1-4) |
|  |  |  |  |  |  |  | Quality of life (QoL-AD) from baseline to month 4 of follow-up, proportion | IG: 33.7 (8.1%) to 33.9 (7.6%)  CG: 34.3 (9.0%) to 34.1 (10.6%)  Incident rate ratio, adjusted (95% CI): -0.03 (-1.90, 1.83), *p*= 0.97 |
|  |  |  |  |  |  |  | Falls from baseline to month 4 of follow-up | Incident rate ratio, adjusted (95% CI): 1.80 (0.94, 2.66), *p*= 0.08 |
|  |  |  |  |  |  |  | Falls from baseline to month 12 of follow-up, proportion | IG: 70/98 (71.4%) vs. 70/143 (48.9%)  Incident rate ratio, adjusted (95% CI): 2.61 (1.73, 3.93), *p*< 0.001 |
|  |  |  |  |  |  |  | Hospitalizations from baseline to month 4 of follow-up | Incident rate ratio, adjusted (95% CI): 2.05 (0.83, 5.15), *p*= 0.12 |
|  |  |  |  |  |  |  | Hospitalizations from baseline to month 12 of follow-up, proportion | IG: 29/98 (29.5%) vs. 36/143 (25.1%)  Incident rate ratio, adjusted (95% CI): 1.84 (0.78, 4.39), *p*= 0.16 |
|  |  |  |  |  |  |  | Deaths at 4 months, proportion | IG: 10 (10.1%) vs. CG: 15 (10.5%)  Incident rate ratio, adjusted (95% CI): 0.90 (0.40, 1.98), *p*= 0.78 |
|  |  |  |  |  |  |  | Deaths at 12 months, proportion | IG: 28/98 (28.5%) vs. CG: 50/143 (35.0%)  Relative risk, adjusted (95% CI): 0.76 (0.50, 1.15), *p*= 0.20 |
| Cateau, 2021  Switzerland | Total: 62  IG: 32  CG: 30 | IG, median (IQR): 87 (80-91)  CG, median (IQR): 84 (78-88) | Inclusion criteria (for the LTCFs involved):   - Caring for a mainly geriatric population - Entered the integrated pharmacy service at least one year before recruitment   Inclusion criteria:   - Resident living in a volunteer nursing home for at least four months - 65 years or older - 5 or more medications prescribed   Exclusion criteria:   - Staff could choose not to offer participation to a specific resident if discussing the possibility of taking part in the trial would cause undue distress to them or their relatives | Pharmacists, nurses, physicians​ | - Education session for the pharmacists performing the intervention (postgraduate course organized by the Institute of Pharmaceutical Sciences of Western Switzerland, University of Geneva) - STOPP/START criteria 2015 version | Baseline data collection for both groups occurred after the validation of the deprescribing plan to the participants of the IG. Follow-up data was collected at month 4. | N. of PIMs, incidence rate ratio (95% CI) | IG vs. CG: 0.972 (0.830, 1.138) |
|  |  |  |  |  |  |  | Quality of life (EQ-5D-5L), mean difference (95% CI) | IG vs. CG: -0.096 (-0.202, 0.010) |
|  |  |  |  |  |  |  | Falls from baseline to follow-up, number of patients (%) | IG: 9 (30%) vs. CG: 9 (33%)  *p=* 0.781 |
|  |  |  |  |  |  |  | Hospitalizations, number of patients (%) | IG: 3 (10%) vs. CG: 1 (4%)  *p=* 0.615 |
|  |  |  |  |  |  |  | Days spent in hospital, mean (SD) | IG: 3.6 (15.8) vs. CG: 0.6 (2.9) |
|  |  |  |  |  |  |  | Deaths at 4 months, proportion | IG: 4 (13%) vs. CG: 2 (7%)  *p=* 0.675 |
| Liou, 2021  Taiwan | Total: 100  IG: 50  CG: 50 | IG: 86.7 (5.6)  CG: 85.7 (3.6) | Inclusion criteria:   - 65 years or older - 5 or more oral medicines daily - 2 or more chronic diseases - complete comprehensive geriatric assessment (CGA) - prescription drugs more than 4 times/day, excluding stat order   Exclusion criteria:   - Previously been included in an intensive medication review intervention led by a pharmacist | Pharmacists, physicians, nurses, nurse assistants​ | - The pharmacists were trained to implement a unified intervention approach which included completing the medication administration record, assessing prescribing appropriateness, surveying the utilization of healthcare resources, and identifying DRPs - No validated tool was used or described (e.g., software, criteria, etc.) | In the IG, medication review visits were offered at months 1, 3, 7, 13, 18 | Number of drugs prescribed, mean change (SD) per patient from baseline to month 18 of follow-up | IG: -1.0 (3.6) vs. CG: -0.6 (3.8)  *p*= 0.700 |
|  |  |  |  |  |  |  | PIMs, mean change (SD) per patient from baseline to month 18 of follow-up | IG: -0.2 (1.0) vs. CG: 0.0 (1.0)  *p*= 0.481 |
|  |  |  |  |  |  |  | DRPs, mean change (SD) per patient from baseline to month 18 of follow-up | 1.6 (1.4) to 0.3 (0.5)  *p*< 0.01 |
|  |  |  |  |  |  |  | Medical problems, mean change (SD) per patient from baseline to month 18 of follow-up | IG: 3.0 (4.0) vs. CG: 0.9 (2.7)  *p*= 0.035 |
|  |  |  |  |  |  |  | Physical function (ADL), mean change (SD) per patient from baseline to month 18 of follow-up | IG: -5.0 (8.3) vs. CG: 3.8 (29.4)  *p*= 0.186 |
|  |  |  |  |  |  |  | Physical function (IADL), mean change (SD) per patient from baseline to month 18 of follow-up | IG: -1.3 (1.5) vs. CG: -1.1 (1.9)  *p*= 0.894 |
|  |  |  |  |  |  |  | Cognitive function (MMSE), mean change (SD) per patient from baseline to month 18 of follow-up | IG: -2.0 (6.3) vs. CG: -2.3 (8.2)  *p*= 0.928 |
|  |  |  |  |  |  |  | Quality of life (EQ-VAS), mean change (SD) per patient from baseline to month 18 of follow-up | IG: -7.5 (24.6) vs. CG: 1.2 (24.4)  *p*= 0.182 |
|  |  |  |  |  |  |  | Hospitalization, mean change (SD) per patient from baseline to month 18 of follow-up | IG: 0.0 (0.2) vs. CG: -0.1 (0.8)  *p*= 0.931 |
|  |  |  |  |  |  |  | ED admission, mean change (SD) per patient from baseline to month 18 of follow-up | IG: 0.0 (0.5) vs. CG: -0.1 (0.7)  *p*= 0.334 |
|  |  |  |  |  |  |  | Deaths at 18 months, proportion | IG: 2 (4%) vs. CG: 7 (14%) |
| Junius-Walker, 2021  Germany | Total: 787  IG: 402  CG: 385 | Overall: 84.3 (7.7)  IG: 84.7 (7.7)  CG: 83.9 (8.1) | Inclusion criteria:  -65 years or older  Exclusion criteria:  -Short-term care  -Life expectancy less than 6 months | Pharmacists, GPs, nursing staff​ | - ATHINA training for pharmacists: two-day training session on IT-based medication review and a one-day training session in geriatric pharmacotherapy - Training for all participating professional groups on medication management - Training sessions for GPs and nurses on polypharmacy, PIMs antipsychotics, medication management, and adverse drug reactions - PRISCUS list 2010 version - ATHINA medication review | Study duration: 13 months. Data was collected at baseline and at 6 months of follow-up | Use of PIMs and neuroleptic drugs, number of residents (%) | IG: 162 (40.6%) vs. CG: 151 (40.4%)  Odds Ratio, adjusted (95%): 0.90 (0.55, 1.46), *p*= 0.762 |
|  |  |  |  |  |  |  | Quality of life (EQ-5D-3L), mean (SD) | IG: 0.54 (0.30) vs. CG: 0.53 (0.31)  *p*= 0.979 |
|  |  |  |  |  |  |  | Falls per resident, mean (SD) | IG: 0.7 (2.1) vs. CG: 0.5 (1.6)  Odds Ratio, adjusted (95% CI): 0.92 (0.45, 1.88), *p*= 0.811  Proportion: IG 39% vs. CG: 30% |
|  |  |  |  |  |  |  | ED admission, number of residents (%) | IG: 77 (24.3) vs. CG: 63 (19.2)  Odds Ratio, adjusted (95% CI): 1.37 (0.84, 2.27), *p*= 0.206 |
|  |  |  |  |  |  |  | Hospitalizations per resident, mean (SD) | IG: 0.4 (0.7) vs. CG: 0.3 (0.6)  Odds Ratio, adjusted (95% CI): 1.39 (0.92, 2.10), *p*= 0.115) |
|  |  |  |  |  |  |  | Deaths, proportion | IG: 58 (14.4%) vs. CG: 48 (12.5%) |
| Lexow, 2022  Germany | Total: 211  IG: 107  CG:104 | IG, median (IQR): 86 (81-90)  CG, median (IQR): 86 (78-90) | Inclusion criteria:   - 65 years or older - 3 or more long-term/chronic medicines (without counting PRN) - 3 or more comorbidities   Exclusion criteria:   - Life expectancy less than 6 months | Pharmacists, physicians (GPs and specialists), nursing staff, community pharmacists​ | - SPCs - Drug-drug interaction database used in public pharmacies (ABDA Database, ABDATA, Eschborn, Germany) - Clinical therapy guidelines - The PCNE Classification V 6.2 - American Geriatrics Society Beers criteria® 2015 version - The PRISCUS list | Data was collected at three different time points: t0 (baseline), t1 (ranging from 6 weeks to 3 months from t0), and t2 (3 months from t1).  A one-time, pharmacist-led medication review per patient was carried out in the IG between t0 and t1.  No intervention was performed between t1 and t2. | Total medication changes from t0 to t2, N. (N. of patients, %) | IG: 385 (87, 90%) vs. CG: 336 (81, 85%)  *p=* 0.980 |
|  |  |  |  |  |  |  | Number of medications from baseline to end of the follow-up, median (IQR) | IG: 7 (5-10) to 7 (5-10)  CG: 8 (6-10) to 9 (7-11)  *p*= 0.005 |
|  |  |  |  |  |  |  | Falls from t0 to t2, N. (N. of patients, %) | IG: 59 (39, 41%) vs. CG: 59 (33, 35%)  *p=* 0.376 |
|  |  |  |  |  |  |  | Hospitalizations from t0 to t2, N. (N. of patients, %) | IG: 38 (31, 32%) vs. CG: 36 (29, 31%)  *p=* 0.783 |
|  |  |  |  |  |  |  | Deaths at t2, proportion | IG: 10 (10%) vs. CG: 8 (8%)  *p=* 0.668 |
| Roughead, 2022  Australia | Total: 282  IG: 136  CG: 146 | Total: 86 (8)  IG: NR  CG: NR | Inclusion criteria:  -4 or more medicines or 1 or more medicines with anticholinergic or sedative properties  Exclusion criteria:  -≥0.40 on the Frailty Index  -Psychogeriatric Assessment Scales <12/21 or MoCA ≤17/30  -Receiving palliative or respite care | Pharmacists, GPs, care home staff, research assistants | - Educational sessions using a standardized training program - No validated tool was used or described (e.g., software, criteria, etc.) | Intervention occurred every 8 weeks for 12 months | Physical function (Frailty Index) observed change from baseline to month 12, mean (SD) | IG: 0.08 (0.076) vs. CG: 0.089 (0.082)  Modeled estimate = intervention – control (95% CI): -0.009 (-0.028, 0.009) *p*= 0.320 |
|  |  |  |  |  |  |  | Cognitive function (MoCA) observed change from baseline to month 12, mean (SD) | IG: -1.89 (4.87) vs. CG: -3.16 (5.88)  Modeled estimate = intervention – control (95% CI): 1.36 (0.01, 2.72) *p*= 0.048 |
|  |  |  |  |  |  |  | Quality of life (EQ-5D) observed change from baseline to month 12, mean (SD) | IG: -0.199 (0.339) vs. CG: -0.159 (0.329)  Modeled estimate = intervention – control (95% CI): -0.023 (-0.110, 0.050) *p*= 0.566 |
|  |  |  |  |  |  |  | Deaths at month 6, proportion | IG: 15 (12.5%) vs. CG: 9 (7.0%) |
|  |  |  |  |  |  |  | Deaths at month 12, proportion | IG: 23 (19.2%) vs. CG: 17 (13.3%) |
| Etherton-Beer, 2023  Australia | Total: 303  IG (blinded): 102  IG (open): 101  CG: 100 | IG (blinded): 85.8 (7.1)  IG (open): 84.8 (7.7)  CG: 85.0 (7.2) | Inclusion criteria:   - 65 years or older - One or more regular medication   Exclusion criteria:   - Moribund or in the terminal phase of illness with a short life expectancy - Exclusion by GP’s or LTCF manager request | Research pharmacists, GPs, care home staff | - List of PIMs - Deprescribing algorithm | Intervention was planned before randomization. Participants were followed for 12 months post-randomization  or until death | Number of unique regular medicines, mean change (95% CI) per patient from baseline to month 12 of follow-up | IG (blinded): -2.4 (-3.2, -1.6), *p*< 0.0001  IG (open): -1.9 (-2.7, 1.0), *p*< 0.0001  CG: 0.0 (-0.7, 0.8), *p*= 0.9116 |
|  |  |  |  |  |  |  | Adverse drug events from baseline to month 12 of follow-up | No differences except for severe adverse events in CG vs. IG groups (*p*= 0.004). The report of an AE was signiﬁcantly associated with a consistently greater number of regular medicines across all three groups. |
|  |  |  |  |  |  |  | Quality of life (EQ-5D-5L), mean (SD) per patient at month 12 of follow-up | IG (blinded): 0.51 (0.32), *p* (vs. CG) = 0.167  IG (open): 0.61 (0.29), *p* (vs. CG)= 0.57  CG: 0.58 (0.29) |
|  |  |  |  |  |  |  | Physical function (Modified Barthel Index), mean (SD) per patient at month 12 of follow-up | IG (blinded): 34 (33), *p* (vs. CG)= 0.12  IG (open): 47 (34), *p* (vs. CG)= 0.49  CG: 43 (33) |
|  |  |  |  |  |  |  | Cognitive function (MMSE), mean (SD) per patient at month 12 of follow-up | IG (blinded): 9.8 (10.2), *p* (vs. CG)= 0.01  IG (open): 14.8 (10.5), *p* (vs. CG)= 0.75  CG: 14.2 (10.5) |
|  |  |  |  |  |  |  | Physical function (Frailty Index), mean (SD) per patient at month 12 of follow-up | IG (blinded): 0.33 (0.10), *p* (vs. CG)= 0.52  IG (open): 0.33 (0.07), *p* (vs. CG)= 0.74  CG: 0.32 (0.10) |
|  |  |  |  |  |  |  | Falls, median per patient (IQR) at 12 months (data provided by the authors) | IG (blinded): 1 (2)  IG (open): 1 (2)  CG: 1 (2) |
|  |  |  |  |  |  |  | Hospitalizations, proportion (data provided by the authors) | IG (blinded): 40 (39.2%)  IG (open): 39 (38.6%)  CG: 33 (33%) |
|  |  |  |  |  |  |  | Deaths at 3 months, proportion | IG (blinded): 5 (4.9%)  IG (open): 10 (9.9%)  CG: 6 (6.0%) |
|  |  |  |  |  |  |  | Deaths at 12 months, proportion | IG (blinded): 20 (20%)  Hazard ratio, adjusted (95% CI): 0.88 (0.47, 1,64), *p* (vs. CG)= 0.693  IG (open): 28 (28%)  Hazard ratio, adjusted (95% CI): 1.78 (0.98, 3.20), *p* (vs. CG)= 0.056  CG: 20 (20%) |
| Holland, 2023  United Kingdom | Total: 882  IG: 454  CG: 428 | Overall: 85.3 (7.7)  IG: 85.1 (7.7)  CG: 85.4 (7.6) | Inclusion criteria (for the LTCFs involved):  -Primarily caring for residents aged over 65 years  Exclusion criteria (for the LTCFs involved):  -Care homes which receive regular (e.g., a monthly visit or more frequently), from a pharmacist, providing other intensive medication-focused services  -Care homes which receive regular (e.g., a monthly visit or more frequently), from another healthcare professional, providing other intensive medication-focused services  -Care homes that are participating in any other study likely to affect the outcome of the trial (e.g., falls intervention study, rehydration study, etc.)  Inclusion criteria:  -65 years or older  -One or more regular medications  -Under the care of the participating GP practice  -Permanently resident in a participating care home  Exclusion criteria:  -Receiving end-of-life care  -Participating in another study | Pharmacist independent prescribers, GPs, care home staff​ | - Training sessions (on managing medicines for older people with complex needs, a personal development framework, and mentorship) for the pharmacist-independent prescriber - STOPP/START criteria 2015 version | Baseline: 6 weeks from randomization. Follow-up: 6 months | Drug Burden Index from baseline to month 6, mean (SD) | IG: 0.72 (0.75) to 0.66 (0.74)  CG: 0.70 (0.69) to 0.73 (0.69)  Rate ratio, adjusted (95% CI): 0.83 (0.74-0.92), *p*< 0.001 |
|  |  |  |  |  |  |  | Quality of life (Barthel Index) from baseline to month 6, mean (SD) | IG: 8.34 (5.78) to 8.12 (5.84)  CG: 7.07 (5.77) to 6.46 (5.66)  Rate ratio, adjusted (95% CI): 1.20 (0.96-1.49), *p*= 0.11 |
|  |  |  |  |  |  |  | Quality of life (EQ-5D-5L) from baseline to month 6, mean (SD) | IG: 0.31 (0.35) to 0.26 (0.35)  CG: 0.29 (0.37) to 0.21 (0.33)  Absolute difference (95% CI): 0.042 (-0.043, 0.052), *p*= 0.86 |
|  |  |  |  |  |  |  | Crude fall rate/year (median [IQR]), proportion (data provided by the authors) | IG: 3.19 (0 [0-2]) vs. CG: 2.56 (0 [0-1])  Rate ratio, adjusted (95% CI): 0.91 (0.66-1.26), *p*= 0.58 |
|  |  |  |  |  |  |  | Hospitalizations per patient at month 6 | Mean (SD): IG: 0.19 (0.50) vs. CG: 0.18 (0.47)  Rate ratio, adjusted (95% CI): 0.90 (0.61-1.32), *p*= 0.57 |
|  |  |  |  |  |  |  | Deaths at 6 months, proportion | IG: 66 (14.7%) vs. CG: 71 (16.6%) |
| **Non-randomized studies** | | | | | | | | |
| King, 2001  Australia | Total: 245  IG: 75  CG: 170 | Overall, mean (SD): 79.8 (NR)  IG:  Mean (SD): 78.9 (NR)  Median (IQR): 80.7 (NR)  CG:  Mean (SD): 80.2 (NR)  Median (IQR): 83.1 (NR) | No specific inclusion/exclusion criteria were reported | GPs, clinical pharmacist, senior nursing staff, physiotherapist, GP project officer | - No validated tool was used or described (e.g., software, criteria, etc.) | Medication review was held weekly for approximately 8 months. Follow-up period: 1 month | Number of unique regular medications, mean change per patient at 1 month (SD) | IG: -0.35 (2.56) vs. CG: -0.03 (1.90)  *p*= 0.37 |
|  |  |  |  |  |  |  | Number of administered medications, mean change at 1 month (SD) | IG: -0.44 (2.45) vs. CG: 0.12 (1.84)  *p*= 0.16 |
|  |  |  |  |  |  |  | Health status/quality of life in the IG due to intervention at 1 month, proportion | Beneficial: 37 (40%)  No change: 50 (54%)  Detrimental: 4 (4%)  Unknown: 1 (1%) |
|  |  |  |  |  |  |  | Adjusted mortality at 1 month, proportion | IG: 7 (6%) vs. CG: 50 (15%)  *p*= 0.07 |
| Lapane, 2006  United States | Total: NR  IG: 4,272  CG: NR | IG:  <65 y: 6.8%  65-74 y: 16.6%  75-84 y: 40.6%  ≥85 y: 36.0%  CG:  <65 y: 6.3%  65-74 y: 15.0%  75-84 y: 35.5%  ≥85 y: 43.3% | No specific inclusion/exclusion criteria were reported | Dispensing pharmacists, consultant pharmacists, prescribers, registered nurses, licensed practical nurses, nurses aides | - Educational sessions for the staff - Structured approach (Fleetwood Model) - PIM alert included medications as deﬁned from the Surveyors’ Guidelines, which was adapted from the American Geriatrics Society Beers criteria® 1997 version, and propoxyphene | Total study period was 17 months with a pre-post design: a) baseline (6 months), b) phased intervention (9 months), and c) full intervention phase (2 months) | PIM reduction from baseline to the end of the intervention period | Hazard rate, adjusted, IG vs. CG (95% CI): 0.86 (0.65, 1.12) |
|  |  |  |  |  |  |  | Hospitalizations, adjusted percentage change from the end of the intervention period compared to the year preceding the study | IG: 9.7% increase  CG: 23.2% increase  Non-statistically significant |
|  |  |  |  |  |  |  | Hospitalizations due to potential adverse drug events, adjusted percentage change from the end of the intervention period compared to the year preceding the study | IG: 10.0% decrease  CG: 24.0% increase  Hazard ratio, adjusted, IG vs. CG (95% CI): 1.01 (0.84, 1.21) |
|  |  |  |  |  |  |  | Deaths, adjusted percentage change from the end of the intervention period compared to the year preceding the study | IG: 19.0% increase  CG: 0.3% decrease  Non-statistically significant |
| Garfinkel, 2007  Israel | Total: 190  IG: 119  CG: 71 | IG: 81.2 (8.3)  CG: 82.0 (8.7) | No specific inclusion/exclusion criteria were reported | Geriatrician (physician-researcher), department physicians, nursing staff | - Algorithm to improve drug therapy in disabled/frail elderly patients | Follow-up period: 12 months from intervention | Medication appropriateness | A total of 332 drugs were discontinued in the IG (CG were participants in which no drug was discontinued). The failure rate was 18% of all patients (N.= 21) and 10% (N.= 33) of all drugs |
|  |  |  |  |  |  |  | ED visits and/or hospitalizations at 12 months, proportion | IG: 11.8% vs. CG: 30%  *p*< 0.002 |
|  |  |  |  |  |  |  | Death at 12 months | IG: 45% vs. CG: 21%  *p*< 0.001 |
| Olsson, 2009  Sweden | Total: 302  IG: 135  CG: 167 | IG: 86 (range: 55-102)  CG: 85 (range: 61-102) | Exclusion criteria:   - Moving to another facility | GPs, nurses | - No validated tool was used or described (e.g., software, criteria, etc.) | Intervention period: 6 months. Follow-up period: 6 months | Number of regular medicines, mean per patient from baseline to month 6 | IG: 6.94 to 6.48, *p*< 0.05  CG: 7.46 to 7.75, *p*< 0.01 |
|  |  |  |  |  |  |  | Medical consultation from baseline to end of the 6-month follow-up period, mean per patient | IG: 4.33 to 5.51  CG: 5.79 to 4.63  *p*< 0.05 |
|  |  |  |  |  |  |  | Hospitalizations from baseline to end of the 6-month follow-up period, mean per patient | IG: 0.08 to 0.11, *p*> 0.05  CG: 0.22 to 0.14, *p*> 0.05  Proportion: IG 10.4% vs. CG 10.9% |
|  |  |  |  |  |  |  | Deaths during the intervention phase and the follow-up period, proportion | IG: 34 (25.2%) vs. CG: 46 (27.5%)  *p*< 0.05 |
| Sankaran, 2010  New Zealand | Total (one group only, single-arm study): 64 | 56 aged over 85  8 aged 50-85 | Inclusion criteria:   - 85 years or older or younger residents on 9 or more medications | Geriatrician, GPs, nurse manager, clinical nurse specialists, community pharmacist, registered nurses, enrolled nurses, caregivers, social worker | - American Geriatrics Society Beers criteria® 2003 version - Two telephone “hotlines”: one for nurses to have advice from a clinical nurse specialist, one for GPs to speak with the geriatrician - Educational sessions to staff performing the review: web and CD course “Assessment Treatment and Rehabilitation Advanced Core Training (ATRACT)” to nurses; training on “Advance Care Planning” to nurses and GPs | The intervention was delivered in an intensive phase that lasted 6 months, followed by a maintenance phase of 6 months. The review was held weekly during the intensive phase and monthly during the maintenance phase | Medications from baseline to end of the medication reviews, total number (proportion) | 466 to 366 (21% reduction) |
|  |  |  |  |  |  |  | Hospitalizations, number (patients) | During the 6 months before the intervention: 34 (26)  During the intensive phase: 25 (21)  During the maintenance phase: 33 (29) |
|  |  |  |  |  |  |  | Unnecessary hospitalizations, number | During the 6 months before the intervention: 4  During the intensive phase: 2  During the maintenance: Not assessed |
|  |  |  |  |  |  |  | Patient days in hospital, median (IQR) | During the 6 months before the intervention: 4 (1-9)  During the intensive phase: 3 (1-8)  During the maintenance phase: 5 (2-9)  *p*= 0.81 before vs. intensive; *p*= 0.61 intensive vs. maintenance |
| Baqir, 2014  United Kingdom | Total (one group only, single-arm study): 422 | NR | No specific inclusion/exclusion criteria were reported | Clinical pharmacists, GPs, care home nurses, consultant from psychiatry of old age service | - Deprescribing algorithm: medication review 4-question framework | Total study period was 12 months, intervention was delivered in an iterative process | Medication appropriateness measures | 90.5% of the patients required intervention. Of them, 70.6% required the stop of medications (mean of 1.7 drugs stopped per patient) |
|  |  |  |  |  |  |  | Adverse events related to the intervention (all reversible), proportion of patients | 9 (2.1%) |
| Jodar-Sanchez, 2014  Spain | Total: 332  IG: 210  CG: 122 | Overall: 81.6 (NR)  IG: 82.2 (6.9)  CG: 80.5 (7.2) | Inclusion criteria:   - Aged 65 years or older - Cognitively intact   Exclusion criteria:  Nonresident individuals in daycare | Pharmacists, GPs | - No validated tool was used or described (e.g., software, criteria, etc.) | The intervention involves conducting multiple interviews with selected patients. Follow-up period from baseline: 12 months | Prescribed medication from baseline to month 12, mean (SD) | IG: 6.4 (3.1) to 5.9 (3.0)  Mean difference: -0.52, *p*<0.001  CG: 4.8 (2.9) to 5.9 (3.2)  Mean difference: +1.03, *p*<0.001 |
|  |  |  |  |  |  |  | Quality of life (EQ-5D) from baseline to month 12, mean (SD) | IG: 0.595 (0.30) to 0.538 (0.33)  Mean difference: -0.058, *p*= 0.002  CG: 0.621 (0.29) to 0.521 (0.32)  Mean difference: -0.100, *p*= 0.003 |
|  |  |  |  |  |  |  | Quality of life (NHP) from baseline to month 12 | At baseline: both groups had similar scores on the six dimensions, except for the pain dimension (IG: 24.7 vs. CG: 29.7, *p*= 0.16). At 12 months: the IG improved only in the sleep dimension (-1.68 points, *p*= 0.43) and the emotional reactions dimension (-1.59 points, *p*= 0.37), whereas CG scores did not improve in any dimension |
|  |  |  |  |  |  |  | Deaths, proportion | IG: 17 (8%) vs. CG: 10 (8%) |
| Pruskowski, 2017  United States | Total (one group only, single-arm study): 47 | 87.5 (NR) | Inclusion criteria:  -Custodial residents defined as someone classified as long-term care (i.e., residing in the nursing facility for more than 100 days) with a documented comfort-focused treatment plan as denoted “Do Not Resuscitate/Intubate” and either “Comfort Measures Only” or “Limited Additional Interventions” via the Pennsylvania Orders for Life Sustaining Treatment (POLST) form | Clinical pharmacist, primary team (attending physician, nurse practitioners, physician assistants)​ | - The model for “Rational prescribing for patients with a reduced life expectancy” was used to identify appropriate medications for deprescribing | Study period: 6 months. Intervention was delivered once at the beginning of the study | Medication appropriateness measures | Thirty-nine recommendations for 23 residents were made by the clinical pharmacist (mean per resident 0.82, range 0-5). Of them, only 10 (26%) were accepted, 1 (3%) was modified, 3 (8%) were rejected, and 25 (64%) had no response within the 120-day response period |
|  |  |  |  |  |  |  | Hospitalizations at 6 months, proportion | 1 (2.1%) |
|  |  |  |  |  |  |  | Deaths at 6 months, proportion | 2 (4.3%) |
| Gaubert-Dahan, 2019  France | Total (one group only, single-arm study): 52 | 84 (9) | Exclusion criteria:  Palliative care residents | Expert geriatrician, physicians in charge, pharmacist in charge, resident’s nurse​ | - STOPP/START criteria 2015 version | Total study period: 5 months. The intervention was conducted at baseline and evaluations at 3 months | STOPP criteria, mean per patient (SD) | 2.0 (1.4), 101 drugs (98.1% of the STOPP-identified drugs) were discontinued |
|  |  |  |  |  |  |  | START criteria, mean per patient (SD) | 0.7 (0.6), 35 drugs (100% of the START-identified drugs) were started |
|  |  |  |  |  |  |  | Changes not associated with STOPP/START criteria | 16 changes (drug switches, medication schedule adjustments, or dosage changes) |
|  |  |  |  |  |  |  | Adverse drug events at 3 months, number | 1 |
|  |  |  |  |  |  |  | Hospitalizations at 3 months, proportion | 2 (3.8%) |
|  |  |  |  |  |  |  | Deaths in 3 months, proportion | 5 (9.6%) |
| Hashimoto, 2019  Japan | Total: 68  IG: 32  CG: 36 | IG: 86.8 (7.1)  CG: 84.9 (7.4) | Inclusion criteria:   - 5 or more medications   Exclusion criteria:   - Receiving palliative care - History of psychiatric disorders - Patients whose prescriptions had already been reviewed - Patients whose outcomes could not be ascertained at the time of assessment | Pharmacists, family physicians, nurses, care workers | - Guidelines for Medical Treatment and its Safety in the Elderly 2015 - STOPP-J 2016 version - Prescription optimization flowchart | First 4 months: enrollment. Follow-up period: 6 months | PIM, mean per patient (SD) from baseline to month 6 | IG: 2.64 (1.31) to 2.39 (1.32)  CG: 2.74 (1.35) to 2.89 (1.70)  *p*= 0.230 |
|  |  |  |  |  |  |  | Number of medications from baseline to month 6 of follow-up, mean (SD) | IG: 7.71 (2.48) to 7.46 (2.62)  CG: 7.41 (2.17) to 7.37 (2.22)  *p*= 0.887 |
|  |  |  |  |  |  |  | Physical function (ADL), mean per patient (SD) from baseline to month 6 | IG: 17.93 (8.52) to 17.82 (8.50)  CG: 17.00 (7.36) to 16.26 (7.35)  *p*= 0.470 |
|  |  |  |  |  |  |  | Quality of life (SF-12), mean per patient (SD) from baseline to month 6 | IG: 449.64 (84.26) to 423.48 (119.48)  CG: 363.37 (81.59) to 362.78 (86.53)  *p*= 0.036 |
|  |  |  |  |  |  |  | Falls, proportion of patients | IG: 1 (3.6%) vs. CG: 6 (22.2%)  *p*= 0.043 |
|  |  |  |  |  |  |  | Hospitalizations, mean per patient (SD) (total/patient) | IG: 0.18 (0.39) vs. CG: 0.15 (0.36)  *p*= 0.766 |
|  |  |  |  |  |  |  | Hospitalizations, proportion of patients | IG: 5 (17.9%) vs. CG: 4 (14.8%)  *p*= 0.766 |
| Mahlknecht, 2019  Germany | Total (one group only, single-arm study): 120 | 85.2 (7.0) | Inclusion criteria:   - 65 years or older - 1 or more prescriptions   Exclusion criteria:   - Acute life-threatening situation - Isolation due to acute infections or multiresistant micro-organisms - Insufficient cognitive performance and no legal representative | GPs, nurses, study assistants, clinical pharmacists | - 3-step educational training addressing medication safety in older adults - InTherAKT-online Platform | Total study period: 3 years. The intervention is divided into two phases: phase 1, with the supervision of the project team, and phase 2 with the unassisted intervention period. Data was collected at t0, before starting the intervention, t1 after the first intervention period (month 8) and t2, after the second intervention period (month 14) | Medication Appropriateness (MAI), mean (SD) from t0 to t2 | 1.9 (1.2) to 1.3 (0.8)  Mean difference, MAI change (CI 95%): -0.6 (-0.8, -0.4), *p*= 0.000 |
|  |  |  |  |  |  |  | Regular prescriptions from t0 to t2, mean (SD) per patient | 8.2 (4.2) to 8.5 (4.1) |
|  |  |  |  |  |  |  | Cognitive function (MMSE) from t0 to t2, mean (SD) per patient | 17.4 (9.3) to 16.6 (9.6) |
|  |  |  |  |  |  |  | Cognitive function (DSS) from t0 to t2, mean (SD) | 4.7 (4.3) to 5.6 (4.8) |
|  |  |  |  |  |  |  | Deaths, proportion | 27 (22.5%) |
| Sanz-Tamargo, 2019  Spain | Total: 234  IG: 112  CG: 122 | IG, median (IQR): 81.5 (65-96)  CG, median (IQR): 81 (65-95) | Inclusion criteria:   - 65 years or older - Patients who have been resident in the center for at least 6 months   Exclusion criteria:   - Life expectancy of less than 3 months | Pharmacists, family physicians, psychologist, nursing supervisor | - Deprescribing algorithm: one drug was discontinued per patient in each intervention, with the withdrawal staggered for furosemide, antidepressants, proton pump inhibitors, benzodiazepines, anti-dementia drugs, and antipsychotics | Weekly visits to each patient until the end of the follow-up period.  Follow-up: 1-12 months, depending on the drug class deprescribed | Medication appropriateness | In the IG, 20.8% of the total medications were identified as PIMs. Of them, 92.5% were successfully deprescribed |
|  |  |  |  |  |  |  | Clinical outcomes | No serious adverse effects or  hospitalizations related to deprescription were reported |
| Garland, 2020  Canada | Total: 691  IG: 409  CG: 282 | IG: 84.1 (8.62)  CG: 85.8 (7.84) | Inclusion criteria:   - 65 years or older   Exclusion criteria:   - Patients with a discharge date within the 3 ﬁrst months of the study | Pharmacists, nurses, physicians, clinical coaches | - Educational intervention delivered to nurses, pharmacists, and physicians, separately - Process algorithm (PEPS care model) - American Geriatrics Society Beers criteria® 2015 version | Total study period: 16 months. Medication review was repeated at 0, 3, 6, 9, and 12 months from baseline | PIM, mean per patient from baseline to end of the follow-up, mean (95% CI) | IG: 1.3 (1.1, 1.6) to 1.1 (0.9, 1.3)  CG: 1.6 (1.3, 1.9) to 1.5 (1.3, 1.8)  Difference in differences test: 0.2, *p*<0.001 |
|  |  |  |  |  |  |  | Number of medications from baseline to month 12 of follow-up, mean (SD) | IG: 10.6 (9.2-12.1) to 8.9 (7.7-10.1)  CG: 13.0 (11.4-14.8) to 12.5 (10.9-14.3)  *p*< 0.001 |
|  |  |  |  |  |  |  | ED visits at 12 months, proportion | IG: 54 (13.2%) vs. CG: 22 (7.8%) |
|  |  |  |  |  |  |  | Hospitalizations at 3 months, proportion | IG: 5/284 (1.8%) vs. CG: 2/209 (1.0%) |
|  |  |  |  |  |  |  | Hospitalizations at 12 months, proportion | IG: 18 (4.4%) vs. CG: 5 (1.8%) |
|  |  |  |  |  |  |  | Deaths at 3 months, proportion | IG: 1/284 (0.4%) vs. CG: 1/209 (0.5%) |
|  |  |  |  |  |  |  | Deaths at 12 months, proportion | IG: 84 (20.5%) vs. CG: 79 (28.0%) |
| Dalin, 2022  Denmark | Total (pre-post single group study): 135 | 82.3 (7.8) | Inclusion criteria:   - 65 years or older - 5 or more regular drugs, including calcium tablets and vitamins | Clinical pharmacologists, GPs | - List of first-choice medications - Danish Deprescribing List - List of anticholinergic medicines - National Database on Drug Interactions | Follow up: 3 months | Medication appropriateness measures | Recommended changes were made to 491 medications (31% of all medications) evaluated. Among these, deprescribing was the most frequent recommendation (78%) |
|  |  |  |  |  |  |  | Symptoms evaluation (ESAS-r), changes pre-intervention vs. post-follow-up | No significant differences: pain (-0.34), tiredness (0.10), drowsiness (0.03), nausea (0.14), loss of appetite (0.42), shortness of breath (-0.03), depression (0.30), and anxiety (-0.02) |
|  |  |  |  |  |  |  | Quality of life (EQ-5D-5L), mean change per patient pre-intervention vs. post-follow-up (CI 95%) | -0.002 (-0.036, 0.176), non-significant |
|  |  |  |  |  |  |  | Deaths at month 3, proportion | 10 (7.9%) |
| Attwood, 2024  United Kingdom | Total: 296  IG: 196  CG: 100 | Overall: 88.7 (6.97)  IG: 89.4 (6.9)  CG: 87.3 (6.9) | No specific inclusion/exclusion criteria were reported | GPs, district nurses, community therapists, acute response practitioners, social care professionals | - IT-assisted Comprehensive Geriatric Assessment, which included two activities: a) holistic medication review (patient goals; long-term conditions review and optimization; medication deprescribing; advance care planning) and b) assessment and optimization of function (including diverse social and clinical endpoints) - Path-fields Tool to screen for and diagnose frailty clinically | Total study period: 2 years and 2 months. Follow-up period: 1 year after intervention | Unplanned hospital admissions, mean change (SD) per patient-years alive compared with the year prior | IG: 0.04 (0.34) vs. CG: 1.01 (0.88)  Treatment difference: -0.97 (0.94)  *p*= 0.16 |
|  |  |  |  |  |  |  | Unplanned bed days, mean change (SD) per patient-years alive compared with the year prior | IG: 1.7 (4.1) CG: 8.4 (8.0)  Treatment difference: -6.8 (9.0)  *p*= 0.45 |
|  |  |  |  |  |  |  | Deaths at 12 months, proportion | IG: 94 (48%) vs. CG: 57 (57%)  *p*= 0.1 |
| Chan, 2024  Australia | Total (one group only, single-arm study): 175 | 84 (9.5) | No specific inclusion/exclusion criteria were reported | Pharmacists, GPs, medical officers, nurses, nursing staff | - STOPP/START criteria 2015 version - Drug Burden Index | Study period: 12 months, including a follow-up period of 1 month | PIM from baseline to month 1, median (IQR) | 2 (1-3) to 1 (0-2)  Change: 0 (-1,0), *p*<0.001 |
|  |  |  |  |  |  |  | Drug Burden Index from baseline to month 1, mean (SD) | 1.54 (0.99) to 1.37 (0.95)  Mean paired difference: -0.17 (-0.24, -0.10), *p*<0.001 |
|  |  |  |  |  |  |  | Deaths at 1 month, proportion | 20 (11%) |

***** If mean (SD) was not available, alternative information provided by the authors was reported (e.g., median (IQR), stratification by age group).

**Abbreviations**: ADL = Activity of Daily Living; AMTS = Abbreviated Mental Test Score; ATHINA = *Arzneimitteltherapiesicherheit in Apotheken*, i.e. German acronym for “Medication therapy safety in pharmacies”; BASDEC = Brief Assessment Schedule Depression Cards; CG = Control group; CI = Confidence interval; CRBRS = Crichton-Royal Behaviour Rating Scale; DRPs = Drug-related problems; DSS = Dementia Screening Scale; ED = Emergency department; EQ-VAS = European quality of life - visual analog scale; ESAS-r = Edmonton Symptom Assessment System-revised; FIM = Functional Independence Measure; GDS = Geriatric Depression Scale; GPs = General practitioners; IADL = Instrumental Activity of Daily Living; IG = Intervention group; InTherAKT = *Initiative zur Therapiesicherheit in der Altenhilfe durch Kooperation und Teamwork*, German acronym for “Initiative for medication safety in long-term care via cooperation and teamwork”; IPET = Inappropriate Prescribing in the Elderly Tool; IQR = Interquartile range; IT = Information technology; LTCFs = Long-term care facilities; MMSE = Mini Mental Status Examination; MNA-SF = Mini Nutritional Assessment – short form; MoCA = Montreal Cognitive Assessment; NHP = Nottingham Health Profile; NR = Not reported; OBRA = Ombudsman Reconciliation Act; PCNE = Pharmaceutical Care Network Europe; PIMs = Potentially inappropriate medications; PIPs = Potentially inappropriate prescriptions; PPO = Potential prescription omission; PRN = *Pro re nata*; QUALID = Quality of life in late-stage dementia; QUALIDEM = Quality of life in dementia; QoL-AD = Quality of Life in Alzheimer’s Disease; RAI = Resident Assessment Instrument; RCI = Resident Classification Instrument; SAPS = Short Assessment of Patient Satisfaction; SD = Standard deviation; SF-12 = Medical outcomes study 12-item Short-Form health survey; SMPA = Swedish Medical Product Agency; SPCs = Summary of Product Characteristics; START = Screening Tool to Alert to Right Treatment; STOPP = Screening Tool of Older Persons' Prescriptions; STOPP-J = STOPP for Japanese.

**Table A5.** Tools used to conduct medication review interventions.

| **Algorithms** |
| --- |
| 5-item Medication Regimen Simpliﬁcation Guide for Residential Aged CarE (MRS GRACE) |
| Algorithm to improve drug therapy in disabled/frail elderly patients |
| ATHINA medication review |
| Deprescribing algorithm (Balson, 2020) |
| Deprescribing algorithm (Potter, 2016; Etherton-Beer, 2023) |
| Deprescribing algorithm: medication review 4-question framework |
| Deprescribing algorithm: one drug was discontinued per patient in each intervention, with the withdrawal staggered for furosemide, antidepressants, proton pump inhibitors, benzodiazepines, anti-dementia drugs, and antipsychotics |
| Prescription optimization flowchart |
| **Anticholinergic scales** |
| Anticholinergic list |
| Anticholinergic Risk Scale |
| Drug Burden Index |
| List of anticholinergic medicines |
| **Criteria/checklists** |
| American Geriatrics Society Beers criteria® 2003 version |
| American Geriatrics Society Beers criteria® 2015 version |
| Danish Deprescribing List |
| IPET |
| List of first-choice medications |
| List of PIMs |
| PCNE Classification V 6.2 |
| Rational prescribing for patients with a reduced life expectancy |
| STOPP-J 2016 version |
| STOPP/START criteria 2008 version |
| STOPP/START criteria 2015 version |
| Surveyors’ interpretive Guidelines |
| **Guidelines** |
| Guidelines for Medical Treatment and its Safety in the Elderly 2015 |
| Guidelines produced by the North West Drug Information Service |
| Local and national guidelines (not specified) |
| SMPA guidelines for drug treatment in the elderly and the demented |
| Swedish list of medications with anticholinergic properties |
| US OBRA guidelines |
| **Interaction checkers** |
| Drug-drug interaction database used in public pharmacies (ABDA Database, ABDATA, Eschborn, Germany) |
| National Database on Drug Interactions |
| PRISCUS list 2010 version |
| www.interaksjoner.no |
| **Multi-approach deprescribing tools** |
| Deprescribing guide consisting of the American Geriatrics Society Beers criteria® 2015 version, STOPP criteria 2015 version, as well as drug interaction checking |
| InTherAKT-online Platform |
| IT-assisted Comprehensive Geriatric Assessment |
| **Pharmacological reference resources** |
| British National Formulary |
| SPCs |

**Abbreviations**: ATHINA = *Arzneimitteltherapiesicherheit in Apotheken*, i.e. German acronym for “Medication therapy safety in pharmacies”; InTherAKT = *Initiative zur Therapiesicherheit in der Altenhilfe durch Kooperation und Teamwork*, German acronym for “Initiative for medication safety in long-term care via cooperation and teamwork”; IPET = Inappropriate Prescribing in the Elderly Tool; IT = Information technology; OBRA = Ombudsman Reconciliation Act; PCNE = Pharmaceutical Care Network Europe; PIMs = Potentially inappropriate medications; SMPA = Swedish Medical Product Agency; SPCs = Summary of Product Characteristics; START = Screening Tool to Alert to Right Treatment; STOPP = Screening Tool of Older Persons' Prescriptions; STOPP-J = STOPP for Japanese.

**Table A6.** Results from meta-regression and assessment of residual heterogeneity for mortality risk at 9-24 months.

|  | **Meta-regression analysis** | | | | **Heterogeneity assessment** | |
| --- | --- | --- | --- | --- | --- | --- |
| Covariate | *p** | Cochran’s Q (df) | P (Q test) | Pooled estimate | τ² ^#^ | R^2^ (%)^†^ |
| None (random-effects MA) | -- | 34.47 (df=14) | *p* = 0.002 | 0.99 [0.86, 1.13] | 0.0331 | -- |
| Age at enrollment | *p* = 0.330 | 30.51 (df=11) | *p* = 0.001 | 1.00 [0.84, 1.17] | 0.0475 | -43.5 |
| Study design^^^ | *p* = 0.930 | 32.31 (df=13) | *p* = 0.002 | 0.99 [0.86, 1.14] | 0.0395 | -19.4 |
| Geographical region° | *p* = 0.054 | 20.85 (df=11) | *p* = 0.035 | 1.01 [0.90, 1.14] | 0.0145 | 56.2 |

°The following 4 geographical regions were considered: America (Canada), Europe (UK, Finland, Norway, Sweden, Spain, Belgium, Switzerland, and Germany), Asia (Israel, Taiwan, and Japan), Oceania (Australia);

^It consists of randomized clinical trials (RCTs) or non-randomized studies;

**p*-value from omnibus Wald-type test of parameters (i.e., study-level covariates included in the model);

#Between-study variance estimated before (total) and after (residual) inclusion of each study-level covariate in the model;

†R^2^ is the proportion of the total between-study variance that is reduced by the effect of the study-level covariate included in the model.

**Abbreviations**: df: degrees of freedom referred to the Cochran’s Q test; MA: meta-analysis.

**Figure A1**. Forest plot of the estimated mean differences (intervention minus control group) of falls per patient at 6 months.


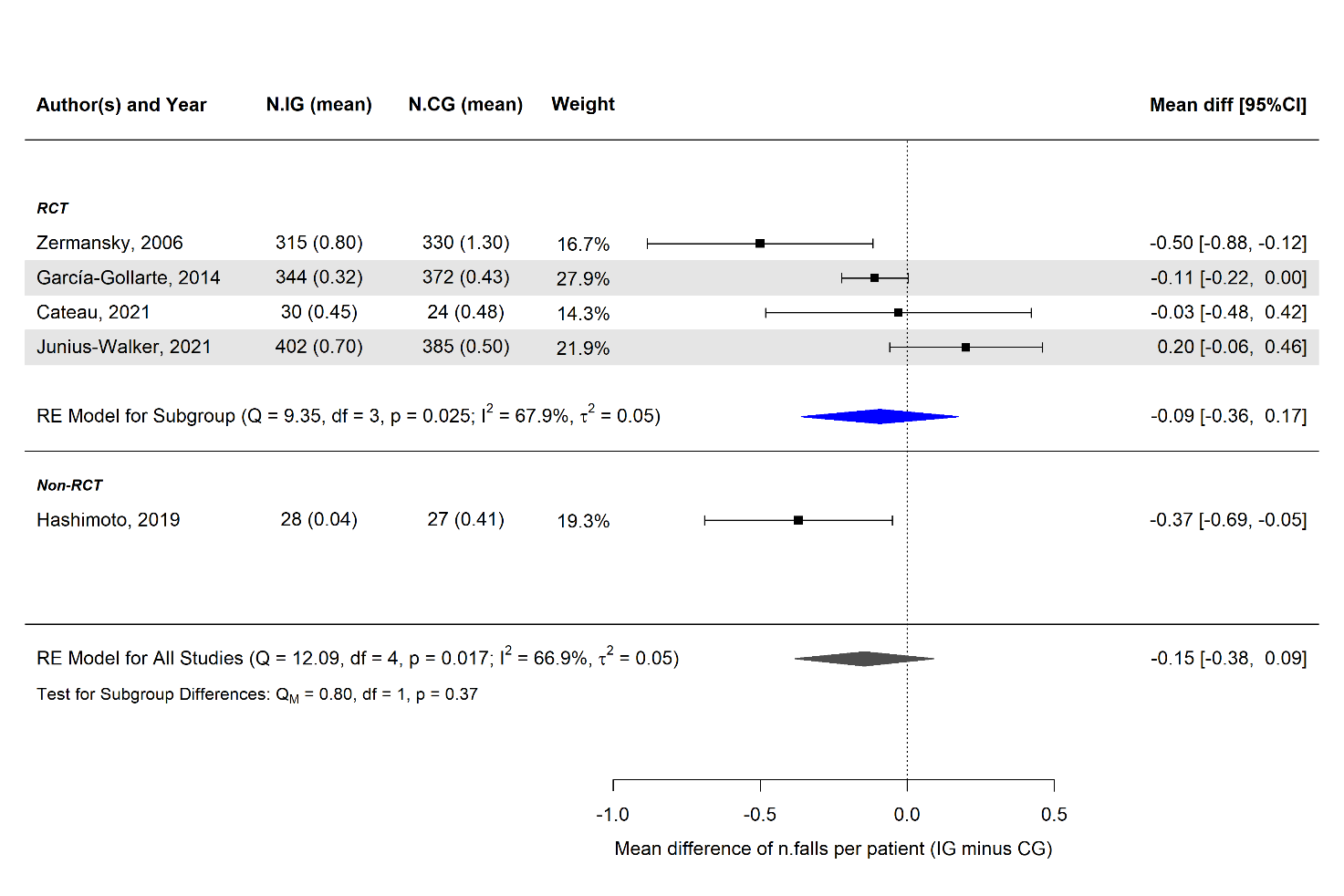


All included studies involved technology- or guideline-based interventions.

Estimates are also stratified according to the study type (subgroup). The summary polygon below each subgroup (filled in blue) shows the results of a fixed or random-effects model for just the studies within that group. The summary polygon at the bottom of the plot (filled in dark grey) shows the results of the model when all studies are analyzed. The test for subgroup difference (QM) reflects the statistical significance of the subgroup covariate when included in a meta-regression model.

**Abbreviations**: N.IG: number of valid patients in the intervention group (IG); N.CG: number of valid patients in the control group (CG); RCT: Randomized Clinical Trials; FE: fixed-effects; RE: random-effects; Q: Cochran’s Q statistic, along with degrees of freedom (df) and its *p*-value; I²: inconsistency measure; τ²: between-study variance.

**Figure A2**. Forest plot of the estimated mean differences (intervention minus control group) of falls per patient at 12 months.


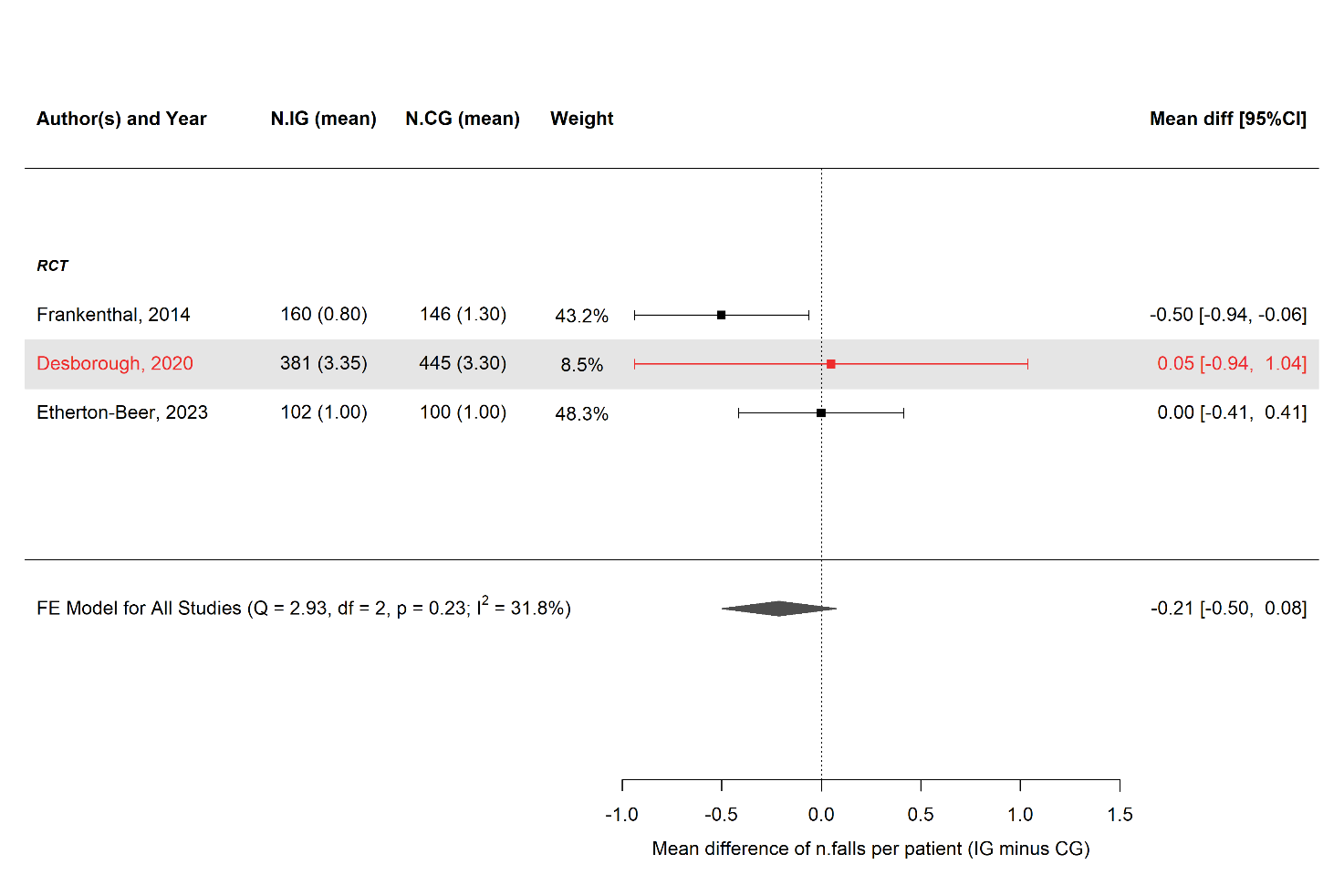


The colors used for the horizontal lines indicate the type of intervention applied within each study: black for technology- or guideline-based interventions and red for no specified intervention tools.

All included studies were randomized clinical trials.

**Abbreviations**: N.IG: number of valid patients in the intervention group (IG); N.CG: number of valid patients in the control group (CG); RCT: Randomized Clinical Trials; FE: fixed-effects; Q: Cochran’s Q statistic, along with degrees of freedom (df) and its *p*-value; I²: inconsistency measure.

**Figure A3**. Forest plot of the estimated mean differences (intervention minus control group) of hospitalizations per patient at 6 or 12 months.


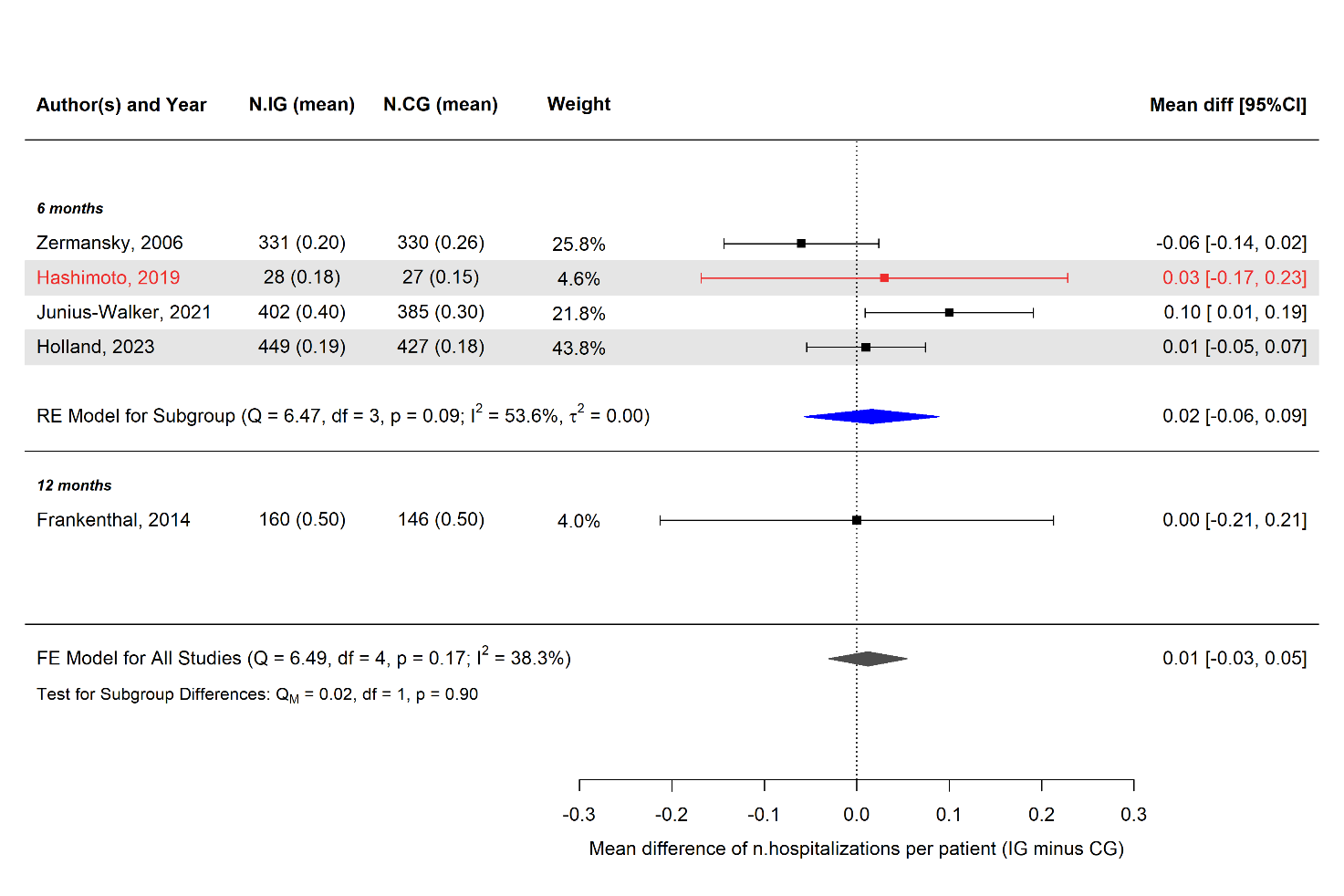


All included studies involved technology- or guideline-based interventions.

The colors used for the horizontal lines indicate the study type: black for randomized clinical trials and red for non-randomized clinical trials.

Estimates are also stratified according to the follow-up duration (subgroup). The summary polygon below each subgroup (filled in blue) shows the results of a fixed or random-effects model for just the studies within that group. The summary polygon at the bottom of the plot (filled in dark grey) shows the results of the model when all studies are analysed. The test for subgroup difference (Q_M_) reflects the statistical significance of the subgroup covariate when included in a meta-regression model.

**Abbreviations**: N.IG: number of valid patients in the intervention group (IG); N.CG: number of valid patients in the control group (CG); RCT: Randomized Clinical Trials; FE: fixed-effects; RE: random-effects; Q: Cochran’s Q statistic, along with degrees of freedom (df) and its *p*-value; I²: inconsistency measure; τ²: between-study variance.

**Figure A4**. Forest plot of the estimated risk ratio (intervention vs. control group) of death evaluated at the last available follow-up.


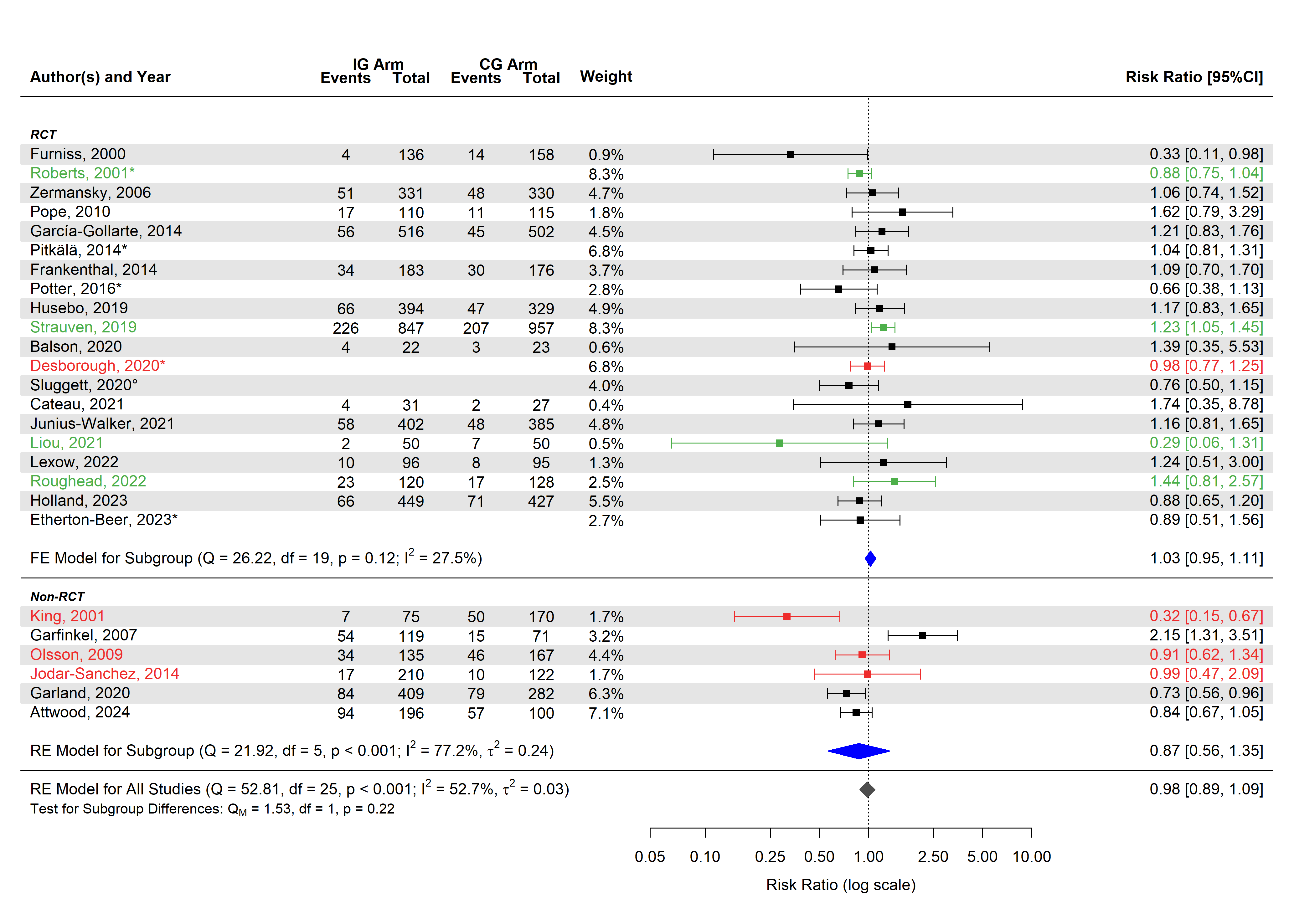


*Risk ratio was estimated indirectly from the published hazard ratio, using established conversion formulae, and its variance was calculated using the delta method; ° Risk ratio was directly reported by the authors.

The colors used for the horizontal lines indicate the type of intervention applied within each study: black for technology- or guideline-based interventions; green for training-based interventions; and red for no specified intervention tools.

Estimates are also stratified according to the study type (subgroup). The summary polygon below each subgroup (filled in blue) shows the results of a fixed or random-effects model for just the studies within that group. The summary polygon at the bottom of the plot (filled in dark grey) shows the results of the model when all studies are analyzed. The test for subgroup difference (Q_M_) reflects the statistical significance of the subgroup covariate when included in a meta-regression model.

**Abbreviations**: IG: intervention group; CG: control group; RCT: Randomized Clinical Trials; FE: fixed-effects; RE: random-effects; Q: Cochran’s Q statistic, along with degrees of freedom (df) and its *p*-value; I²: inconsistency measure; τ²: between-study variance.

**Figure A5**. Funnel plot of risk ratio of death at 9-24 months with estimated Kendall's tau coefficient to test for asymmetry.


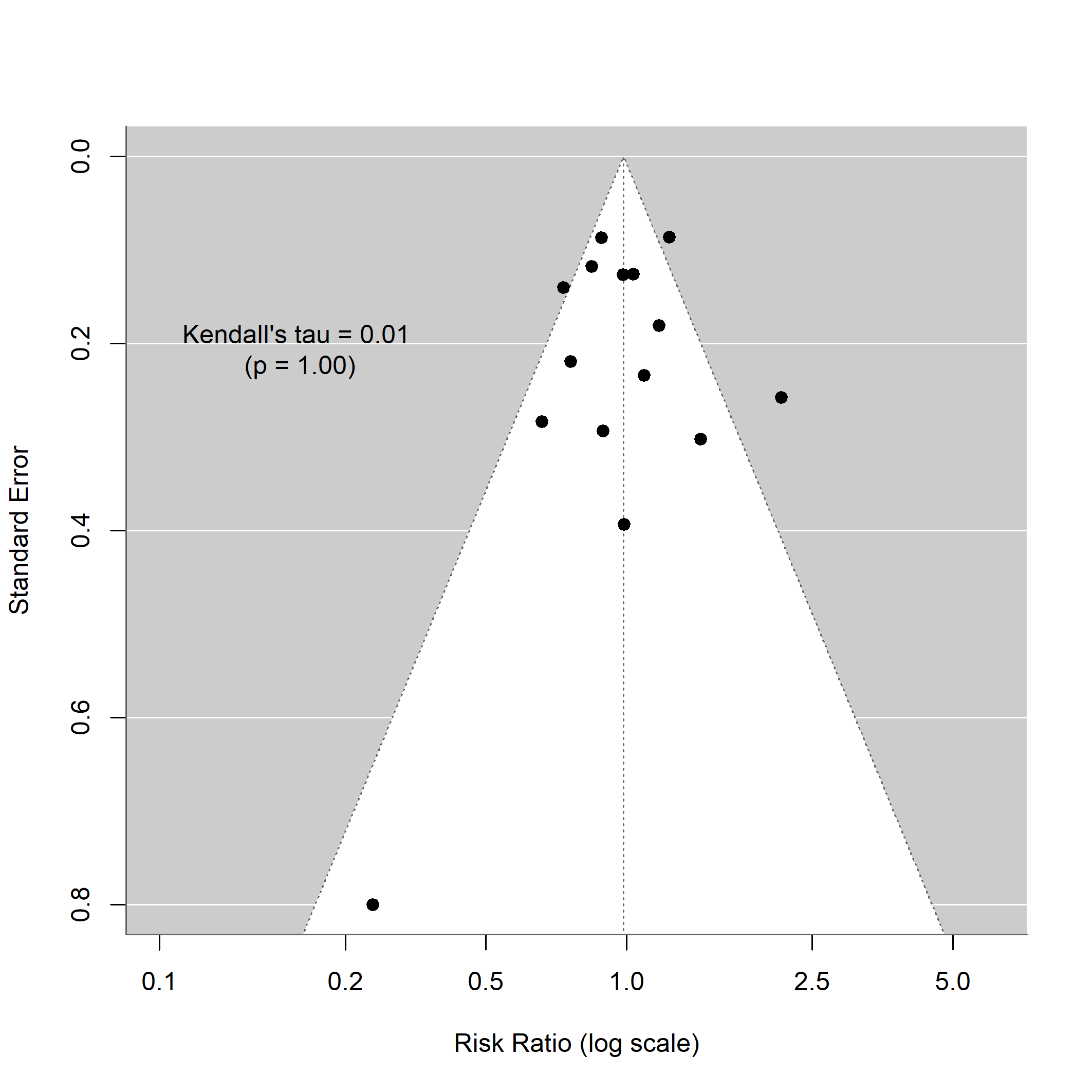


**Figure A6.** Risk of bias of the randomized controlled trials included in the systematic review.


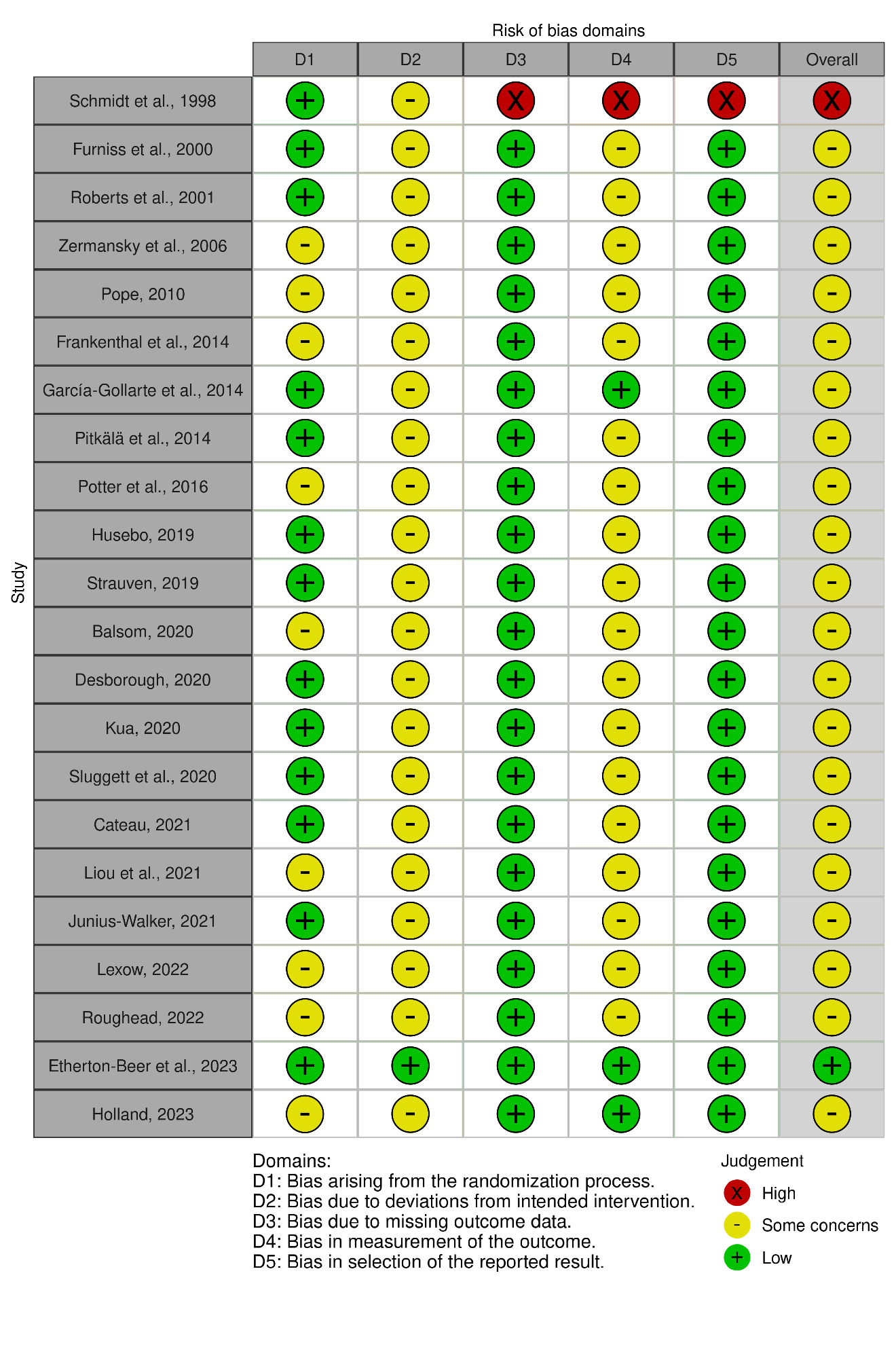


**Figure A7**. Summary plot of the risk of bias of the randomized controlled trials included in the systematic review.


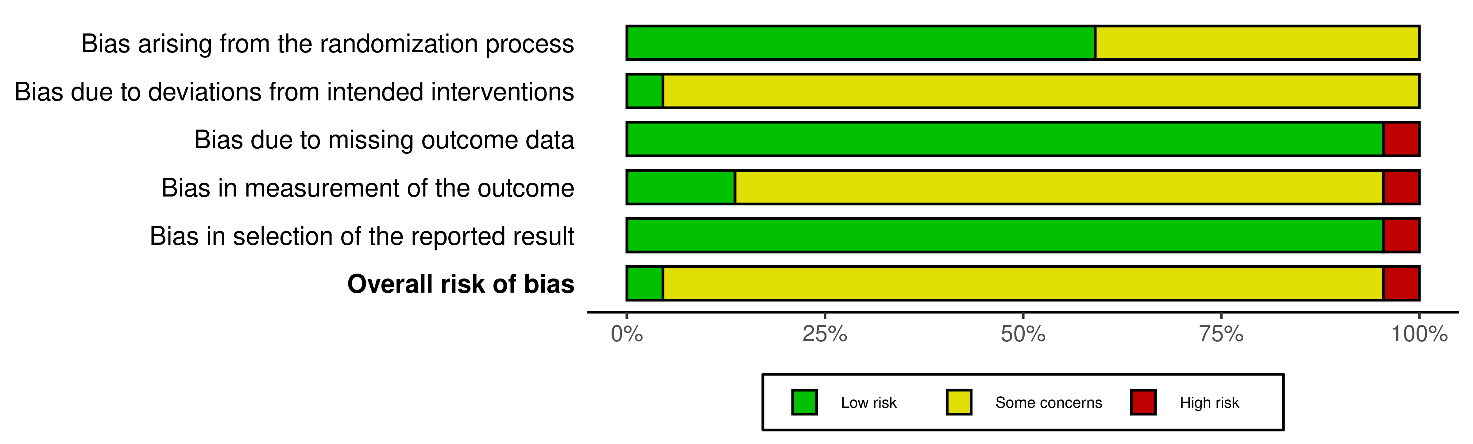


**Figure A8.** Risk of bias of the non-randomized studies included in the systematic review.


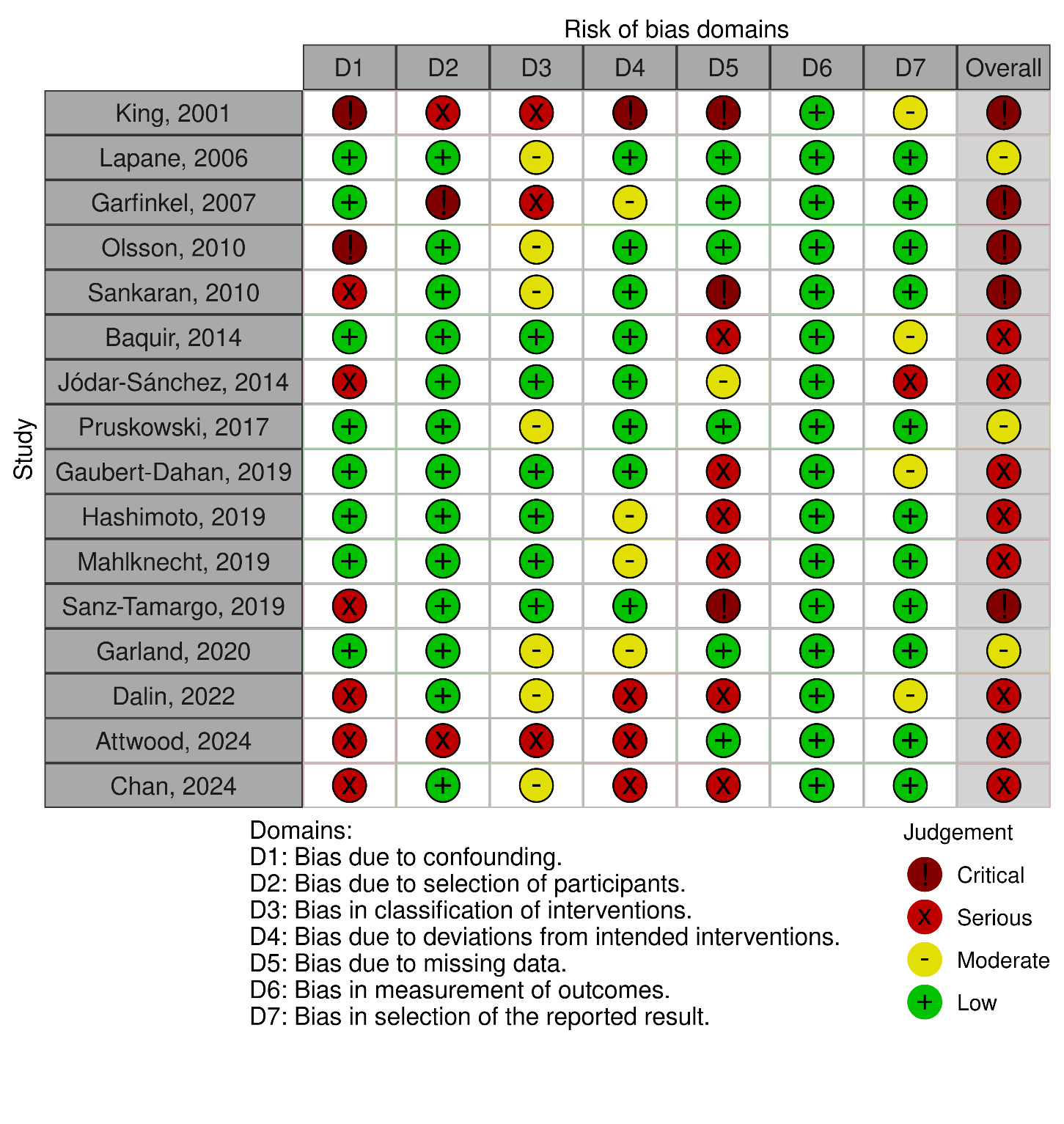


**Figure A9**. Summary plot of the risk of bias of the non-randomized studies included in the systematic review.


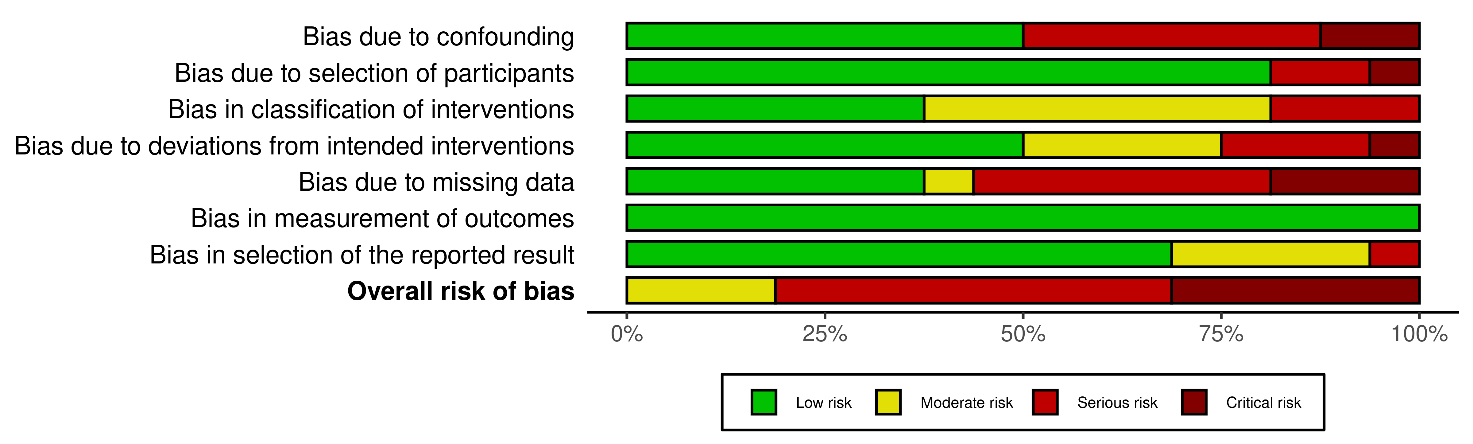


**Statistical Methods**

In this section we show how to obtain an estimate of a risk ratio (RR), and its variance, by knowing the reported hazard ratio (together with its variance or 95% confidence interval) and the proportion of events in the reference group.

Under the following assumptions that:

- The event incidence rate is constant over time within each group
- The cohort is fixed (i.e., non-dynamic enrolment)
- There is no loss to follow-up
- The cohort does not experience events of a nature different from the event of interest

Then it can be shown that the size of the risk cohort at time zero (N_0_) is reduced over time (t > 0) by the occurrence of the events of interest, following a negative exponential function of the form:

$$N_{t}=N_{0}{\cdot e}^{-(T\cdot\Delta t)}$$

Where N_t_ is the cohort size at time t, T is the incidence rate, and Δ_t_ is the time interval between time zero and t. This relationship is well-established in the literature.

Using basic algebra, the RR of an event can be related to its incidence rate:

$$\text{R}=1-\frac{N_{t}}{N_{0}}=1-e^{-\left( T\cdot\Delta t \right)}$$

Thus, the incidence rate can be expressed in terms of the risk (or, more specifically, cohort numbers):

$$ln\left( \frac{N_{t}}{N_{0}} \right)=-\left( T\cdot\Delta t \right)$$

$$T=\frac{ln\left( N_{0} \right)-ln\left( N_{t} \right)}{\Delta t}$$

Therefore, the incidence rate ratio (i.e., the hazard ratio – HR) between the two groups (e.g., group A and group B – the latter is considered as the reference group) is calculated as:

$HR=\frac{T_{A}}{T_{B}}=\frac{-T_{A}}{{-T}_{B}}=\frac{-\left( ln\left( N_{0,A} \right)-ln\left( N_{t,A} \right) \right)}{-\left( ln\left( N_{0,B} \right)-ln\left( N_{t,B} \right) \right)}=\frac{ln\left( N_{t,A} \right)-ln\left( N_{0,A} \right)}{ln\left( N_{t,B} \right)-ln\left( N_{0,B} \right)}=\frac{ln\left( \frac{N_{t,A}}{N_{0,A}} \right)}{ln\left( \frac{N_{t,B}}{N_{0,B}} \right)}=\frac{ln\left( 1-1+\frac{N_{t,A}}{N_{0,A}} \right)}{ln\left( 1-1+\frac{N_{t,B}}{N_{0,B}} \right)}=\frac{ln\left( 1-\left( 1-\frac{N_{t,A}}{N_{0,A}} \right) \right)}{ln\left( 1-\left( 1-\frac{N_{t,B}}{N_{0,B}} \right) \right)}=\frac{ln\left( 1-\text{R}_{A} \right)}{ln\left( 1-\text{R}_{B} \right)}$

Finally, by defining the risk ratio (RR) as the ratio of R_A_ to R_B_ it can be shown that:

$$HR=\frac{ln\left( 1-\text{R}_{A} \right)}{ln\left( 1-\text{R}_{B} \right)}=\frac{ln\left( 1-\text{R}_{B}\cdot RR \right)}{ln\left( 1-\text{R}_{B} \right)}$$

From which it follows, by inverse formula, that:

$$RR=\frac{1-e^{HR\cdot ln(1-\text{R}_{B})}}{\text{R}_{B}}$$

Now, as the natural logarithm of HR (i.e. $ln\left( HR \right)$) is asymptotic normal with zero mean and estimated variance approximately equal to $\hat{s}^{2}$: $ln\left( HR \right)\sim N(0,\hat{s}^{2})$

where $\hat{s}^{2}$is the sum of the reciprocal number of events observed in the two groups being compared:

$$\hat{s}^{2}=\frac{1}{{n.events}_{A}}+\frac{1}{{n.events}_{B}}$$

it is easy to see that $ln\left( RR \right)$ is also a function of the random variable $ln\left( HR \right)$, which is also asymptotically normal.

Note that usually $\hat{s}^{2}$ can directly be computed from reported HR’s 95% confidence interval, p-value or standard error.

*The use of delta method*

To derive the approximate distribution of a function of an asymptotically normal random variable, the delta method can be used.

For simplicity of notation, from now on, we define the $ln\left( HR \right)$ as *X* and R_B_ as r.

Therefore, the $ln\left( RR \right)$ can be represented as a function $g\left( . \right)$ of $ln\left( HR \right)$, as follows:

$ln\left( RR \right)=g\left( X \right)=ln\left( \frac{1-e^{(e^{X})\cdot ln(1-r)}}{\text{r}} \right)=ln\left( -\frac{\left( 1-r \right)^{e^{X}}-1}{\text{r}} \right)$ where $X=ln\left( HR \right)$ and $r=\text{R}_{B}$

To use the delta method, we must first compute the first derivative for the function $g\left( X \right)$:

$$g'\left( X \right)=\frac{d}{dX}g\left( X \right)=\frac{d}{dX}ln\left( -\frac{\left( 1-r \right)^{e^{X}}-1}{\text{r}} \right)=\frac{e^{X}\cdot\left( 1-r \right)^{e^{X}}\cdot ln(1-r)}{\left( 1-r \right)^{e^{X}}-1}$$

Thus an approximation of the expectation and variance of $ln\left( HR \right)$ can be obtained as follows:

$$E\left[ ln\left( RR \right) \right]=E\left[ g(X) \right]\approx g(E\left[ X \right])$$

Since X has zero mean, $E\left[ X \right]=0$, we obtain:

$$E\left[ ln\left( RR \right) \right]\approx g\left( 0 \right)= ln\left( -\frac{\left( 1-r \right)^{e^{0}}-1}{\text{r}} \right)=ln\left( -\frac{1-r-1}{\text{r}} \right)=ln(1)=0$$

To calculate its variance, using the delta method, the following approximation is used:

$$Var\left[ ln\left( RR \right) \right]=Var\left[ g(X) \right]\approx\left( g^{'}\left( E\left[ X \right] \right) \right)^{2}\cdot Var\left[ X \right]=\left( g^{'}\left( 0 \right) \right)^{2}\cdot\hat{s}^{2}$$

We start the calculation of $g^{'}(0)$ as follows:

$$g^{'}(0)=\frac{e^{0}\cdot\left( 1-r \right)^{e^{0}}\cdot\ln\left( 1-r \right)}{\left( 1-r \right)^{e^{0}}-1}=\frac{\left( 1-r \right)\cdot ln\left( 1-r \right)}{-r}$$

This term can be plugged into the previous equation, to obtain:

$$Var\left[ ln\left( RR \right) \right]\approx\left( \frac{\left( 1-r \right)\cdot ln\left( 1-r \right)}{r} \right)^{2}\cdot\hat{s}^{2}$$

In summary, the natural logarithm of RR is also asymptotically normal with zero mean and an estimated variance proportional to that calculated for $ln\left( HR \right)$:

$$ln\left( RR \right) \sim N\left( \mu=0,\sigma^{2}=\left( \frac{\left( 1-r \right)\cdot ln\left( 1-r \right)}{r} \right)^{2}\cdot\hat{s}^{2} \right)$$
